# Community movement and COVID-19: a global study using Google's Community Mobility Reports

**DOI:** 10.1017/S0950268820002757

**Published:** 2020-11-13

**Authors:** M. Sulyok, M. Walker

**Affiliations:** 1 Institute of Tropical Medicine, Eberhard Karls University, University Clinics Tübingen, Wilhelmstr. 27, 72074, Tübingen, Germany; 2 Department of Pathology, Institute of Pathology and Neuropathology, Eberhard Karls University, University Clinics Tübingen, Liebermeisterstr. 8, 72076, Tübingen, Germany; 3 Department of the Natural and Built Environment, Sheffield Hallam University, Howard Street, S1 1WB, Sheffield, UK

**Keywords:** COVID-19, Google, movement, social distancing

## Abstract

Google's ‘Community Mobility Reports’ (CMR) detail changes in activity and mobility occurring in response to COVID-19. They thus offer the unique opportunity to examine the relationship between mobility and disease incidence. The objective was to examine whether an association between COVID-19-confirmed case numbers and levels of mobility was apparent, and if so then to examine whether such data enhance disease modelling and prediction. CMR data for countries worldwide were cross-correlated with corresponding COVID-19-confirmed case numbers. Models were fitted to explain case numbers of each country's epidemic. Models using numerical date, contemporaneous and distributed lag CMR data were contrasted using Bayesian Information Criteria. Noticeable were negative correlations between CMR data and case incidence for prominent industrialised countries of Western Europe and the North Americas. Continent-wide examination found a negative correlation for all continents with the exception of South America. When modelling, CMR-expanded models proved superior to the model without CMR. The predictions made with the distributed lag model significantly outperformed all other models. The observed relationship between CMR data and case incidence, and its ability to enhance model quality and prediction suggests data related to community mobility could prove of use in future COVID-19 modelling.

## Introduction

COVID-19 is a highly infectious viral infection, and the main route of transmission is thought to be through respiratory droplets [1, 2]. The level of COVID-19 transmissibility is greater than for other closely related conditions, such as the SARS virus [3]. Those affected are infectious prior to exhibiting symptoms of illness, or remain unaware of infection because they experience only mild symptoms or are asymptomatic; factors which promote further transmission of the disease [2, 4].

Given the highly infectious nature of COVID-19, reducing levels of social interaction and community movement have been seen as key in reducing the rates of COVID-19 transmission [5, 6]. Recommendations have included the practising of social distancing, self-isolation or quarantine, and increasing levels of personal hygiene [7–9]. Such recommendations were followed by more formal, more stringent and often legally imposed governmental restrictions on personal movement which have included ‘stay at home’ orders, closure of non-essential retail units and schools, and banning of sports and entertainment gatherings [10].

The implementation of such measures in response to infectious disease outbreaks is not new; methods aiming to reduce social contact and limit mobility being used for centuries [11–13]. More recently, measures restricting social interaction and movement have been used in response to the SARS and MERS epidemics which occurred in the last decades [11, 14–16]. That movement affects disease transmission and incidence has been shown in numerous studies [e.g. 17, 18]. However, although the connection between mobility and disease has been known for centuries, the detailed quantitative study of this relationship has been difficult. Measuring and quantifying the levels of social interaction and mobility, over large geographical areas, and for large populations is often not feasible.

However, over the last 20 years, technological progress has meant that potential new sources of data providing information on population-wide movement patterns have become available. During this time, mobile phone and Internet usage has become almost ubiquitous. Recording of user behaviour, often also including locational information, has provided detailed new sources of data relating to mobility [19]. Epidemiologists have been eager to utilise such datasets for disease monitoring and surveillance [20]. Notable studies using such data sources include Wesolowski *et al.* [21], who modelled the spread of malaria in Kenya using the records from 15 million mobile phone users. Finger *et al*. were able to use mobile phone records to monitor the effect mass gatherings had on cholera outbreaks [22]. Other diseases similarly studied include cholera [23] and dengue fever [24].

In response to the COVID-19 outbreak, Google released data collated from those accessing its applications using mobile and handheld devices. These Google ‘Community Mobility Reports’ (CMR) [25] show changes in activity and mobility at different location types, compared to before the spread of COVID-19 globally. These datasets are a useful, and global, measure of social activity and movement. Uniquely, they allow comparison between countries. These reports provide the opportunity to study the relationship between social activity and mobility and COVID-19 incidence. In the absence of other global sources of data for these factors, Google's CMR data provide a good indication of the effect health recommendations and governmental restrictions have had on social activity and movement.

The main aim of this study was thus to examine the relationship between mobility and confirmed case numbers for COVID-19 globally, and to ascertain whether cross-country patterns in this relationship were apparent. Such patterns could reflect the range of movement restrictions implemented [10], but could also be due to other cultural or socio-economic differences [26, 27]. Another aim was to integrate CMR data into disease models, to assess whether it could enhance model quality and prediction. The experimental hypothesis is that as COVID-19 case occurrence increases, related reductions in mobility will occur; such patterns are expected internationally due to the increasingly globalised nature of communication channels.

## Methods

### Google Community Mobility Reports

These were accessed on 23 June 2020 and data for 135 countries downloaded, spanning the period from 15 February 2020 until 19 June 2020 [25]. Google's CMR collates data from those accessing Google applications with smartphones or handheld devices who allow recording of ‘location history’ [28]. Individual user presence and time spent at specific location categories is collated to indicate activity. Data are categorised into six discrete categories, which can be summarised as ‘retail and recreation’, ‘parks’, ‘groceries and pharmacies’, ‘workplaces’, transport ‘transit’ hubs and ‘residential’ areas. Increases in the categories ‘parks’ and ‘residential’ are indicative of decreased mobility, as they suggest increased activity in locations around the home environment. The other four categories are more indicative of general mobility as they are related to activity around workplaces, retail outlets and use of public transportation.

CMR provides the percentage change in activity at each location category compared to that on baseline days before the advent of COVID-19 (a 5-week period running from 3 January 2020 to 6 February 2020). Daily activity changes are compared to the corresponding baseline figure day, with for example, data on a Monday being compared to corresponding data from the baseline series for a Monday. Baseline day figures are calculated for each day of the week for each country, and are calculated as the median value [25]. The values thus represent the relative change in percentages compared to baseline days, not absolute number of visitors. Missing values were returned if activity was too low upon a specific day and thus failed to achieve the anonymity threshold set by Google.

### COVID-19-confirmed cases

Corresponding data on the daily number of confirmed COVID-19 cases were downloaded on 13 July 2020 from the John Hopkins COVID-19 data repository situated on github [29].

### Cross-correlation analyses

Correlation analyses were performed using Kendall's *τ* due to the non-parametric nature of the data. Kendall's *τ* correlations were performed using a ±28-day lag. The *τ* value representing the strongest negative or positive correlation, and the corresponding lag in days were tabulated and illustrated as heat-map coded world maps for each CMR category.

Expected was a negative correlation between case numbers and activity in those categories indicative of mobility (‘retail and recreation’, ‘grocery and pharmacy’, ‘transit’, ‘workplace’), and a positive correlation for those two categories (‘parks’, ‘residential’) indicative of sedentary behaviour. A ±28-day lag was chosen in order to encompass the incubation period for COVID-19, some studies reporting that it can extend to 15 days [30]. Examining data using such a lag also takes into account that testing for infection often only occurs some time post-symptom onset, and also the delays occurring between testing, confirmation of infection and updating of official figures.

Results were summarised and tabulated on the country (Supplementary material S1) and on the continent-wide level (Tables 1 and 2). Multiple group comparisons were done with the Kruskal–Wallis test, pairwise comparisons with the Dunn test (with Holm correction to multiple testing) on the continent-level data. Model-based clustering was performed on the scaled data excluding missing variables. We used the mclust package [31] to select the optimal model based on Bayesian Information Criteria (BIC) for EM algorithm initialised by hierarchical clustering for parameterised Gaussian mixture models.

**Table 1.** Continent-level summaries of maximum Kendal's *τ* correlations

	Africa	Asia	Europe	Oceania	South America	North America	Adjusted *P*-value
Retail and recreation	−0.35 (−0.47 to −0.24)	−0.46 (−0.59 to −0.33)	−0.63 (−0.73 to −0.55)	−0.51 (−0.66 to −0.33)	0.33 (−0.43 to 0.37)	−0.38 (−0.52 to −0.25)	0.012
Grocery and pharmacy	−0.31 (−0.42 to −0.16)	−0.40 (−0.51 to 0.24)	−0.51 (−0.56 to −0.38)	−0.46 (−0.57 to −0.32)	0.29 (−0.40 to 0.36)	−0.38 (−0.50 to −0.26)	0.084
Parks	−0.39 (−0.48 to −0.23)	−0.49 (−0.63 to 0.30)	−0.34 (−0.48 to 0.33)	−0.40 (−0.59 to −0.07)	−0.02 (−0.52 to 0.33)	−0.38 (−0.46 to −0.33)	0.698
Transit stations	−0.35 (−0.46 to −0.23)	−0.42 (−0.62 to 0.28)	−0.65 (−0.72 to −0.59)	−0.51 (−0.68 to −0.32)	0.37 (−0.22 to 0.45)	−0.37 (−0.51 to −0.28)	0.012
Workplaces	−0.32 (−0.44 to −0.18)	−0.47 (−0.63 to −0.16)	−0.58 (−0.66 to −0.50)	−0.50 (−0.67 to −0.31)	0.34 (0.31–0.47)	−0.37 (−0.47 to −0.23)	0.012
Residential	0.40 (0.26–0.49)	0.53 (−0.30 to 0.66)	0.57 (0.54–0.66)	0.42 (0.06–0.65)	−0.31 (−0.42 to 0.35)	0.36 (0.28–0.48)	0.014

Continent-level aggregate summaries of Kendall's *τ* analyses between confirmed case numbers and Google CMR data. Results show the *τ*-values corresponding to the strongest correlations. Median values. IQR (in brackets).

**Table 2.** Continent-level summaries of cross-correlations

	Africa	Asia	Europe	Oceania	South America	North America	Adjusted *P*-value
Number of countries	24	32	36	4	10	16	
Retail and recreation	−22.50 (−27.25 to 1.00)	−2.00 (−24.25 to 12.00)	2.00 (−4.00 to 4.75)	8.50 (6.75–11.25)	27.00 (−4.25 to 28.00)	1.50 (−11.25 to 16.25)	0.078
Grocery and pharmacy	−19.00 (−26.00 to 1.00)	−1.00 (−24.25 to 13.25)	3.00 (−3.25 to 4.00)	9.00 (2.50–12.00)	27.00 (−2.00 to 28.00)	−3.00 (−14.75 to 11.50)	0.195
Parks	−1.50 (−24.25 to 12.00)	−12.00 (−26.50 to 7.00)	−0.50 (−15.50 to 23.50)	9.00 (−2.50 to 12.50)	14.00 (−20.25 to 27.00)	−7.00 (−19.25 to 11.25)	0.698
Transit stations	−4.00 (−25.25 to 2.50)	2.50 (−19.00 to 15.50)	1.50 (−3.00 to 5.00)	8.00 (5.75–11.50)	27.50 (8.25–28.00)	−5.50 (−27.25 to 10.75)	0.078
Workplaces	−16.50 (−24.25 to 1.75)	−2.00 (−23.25 to 14.00)	3.50 (−2.00 to 6.00)	9.00 (6.50–12.50)	28.00 (27.25–28.00)	−3.00 (−12.50 to 14.50)	0.012
Residential	−15.00 (−27.25 to 1.75)	−4.00 (−24.25 to 12.50)	−0.50 (−2.25 to 6.00)	5.00 (−4.00 to 8.75)	26.50 (0.25–28.00)	1.00 (−17.25 to 16.25)	0.012

Continent-level aggregate summaries of Kendall's *τ* cross-correlation analyses between confirmed case numbers and Google CMR data. Results show the number of days case numbers were lagged which resulted in the strongest correlations. Median values. IQR (in brackets).

### Modelling

CMR data were integrated into models based upon each country's case incidence.

Mixed-effects random intercept generalised additive models were fitted to the data using incident case numbers as the explained variable to all subsets of data using a Tweedie distribution type. Countries were added as random intercepts. This modelling technique was chosen because of the data structure, with their being repeated measurement values, and a high number of grouping factor levels. Similar mixed-effects approaches were used by other authors including Kraemer *et al*. and Chan *et al*. [32–34]. However, in contrast to these studies, here, we used smoothed variables and distributed lag models with a multilevel generalised additive modelling approach.

Three models were established. First, the smoothed numerical date was used as the explanatory variable. Second, smoothed data for five of the CMR location categories were additionally added as explanatory variables. Change in ‘parks’ mobility was omitted from modelling; increases in ‘park’ activity was expected for northern hemisphere countries during this spring period. Third, the smoothed numerical date was applied as the explanatory variable, but instead of adding data for the five CMR location categories, a spline-described lag of ±14 days for each category was created and used in the distributed lag model.

### Model comparison and validation

Model performance was compared using the BIC. Then each model was compared to each other (Supplementary material S2). To validate the models, we made predictions of the incident case numbers for the time interval from 19 June 2020 to 08 September 2020. Data were obtained on 13 September 2020 as described previously. To compare the predictive performance of the models, we calculated root-mean-square-errors (RMSE) of the different predictions by comparing the predicted values with the reported ones. Comparisons of predictions were also made by using the two-sided Diebold–Mariano test.

Observations with missing values were omitted from calculations. All calculations were performed in R version 3.6.3 using the dlnm, mgcv and lme4 packages. The statistical code together with the data is provided in Supplementary material S1.

## Results

### Cross-correlation analyses

**Fig. 1.**
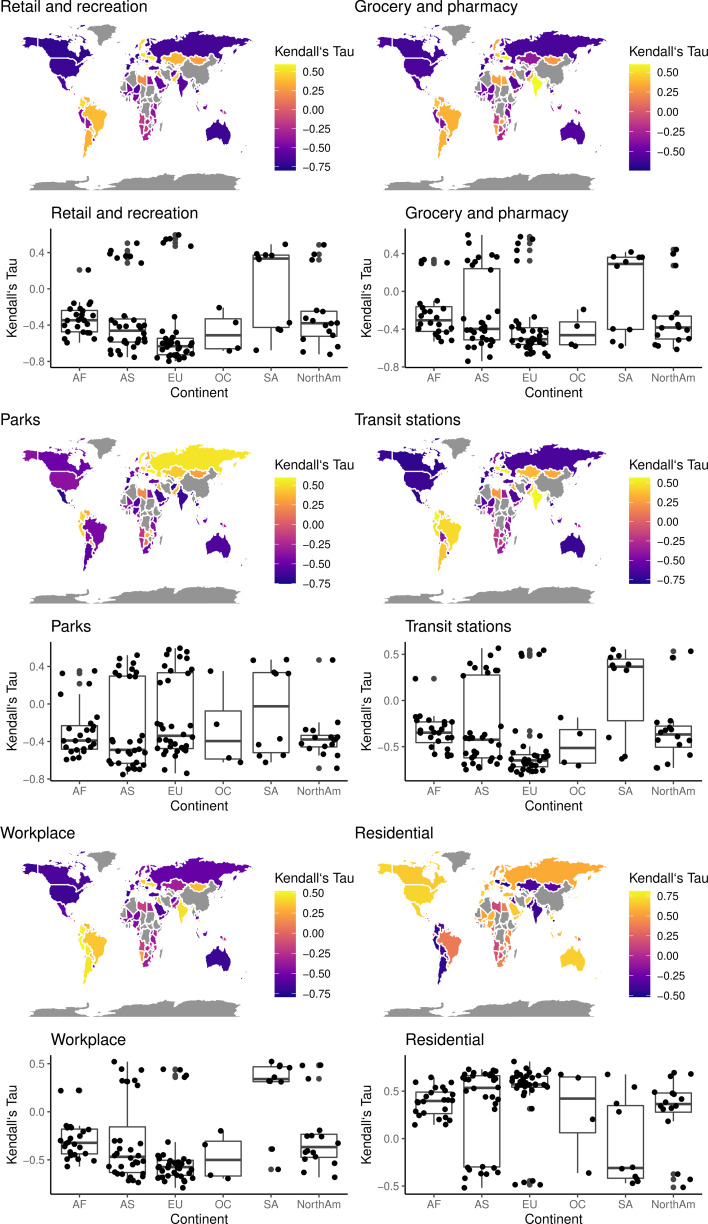
Maximum absolute *τ* values. Results of Kendall's *τ* cross-correlation between COVID-19-confirmed case number and measures of community activity. Strong continent-wide regional patterns are apparent. Generally for the four categories indicative of mobility (‘retail and recreation’, ‘grocery and pharmacy’, ‘workplace’ and ‘transit’) strong negative correlations were observed across countries of North America, Russia, Australia, India and Western Europe. Positive relationships are seen in the South Americas, Eastern Europe, India and Southern Africa. For ‘residential’ activity, which is indicative of increased sedentary behaviour, the opposite was generally observed. For ‘parks’ the picture was mixed, possibly reflecting the difference nature of legal restrictions on a country by country basis; some countries implemented lockdown while others did not, some permitted outdoors exercise, others not [10].

Figure 1 shows the strongest level of correlation, as Kendall's *τ*, that was found between the COVID-19 case incidence for each country and daily percentage change in activity for each location category. A positive correlation indicates that as COVID-19 case numbers increased, there was an increase in activity indicated by data on that location category. A negative correlation indicates that while COVID-19 case numbers rose, there was a decline in activity in that location category or vica versa.

Particularly strong negative correlations are apparent for prominent countries of Western Europe, across the North Americas, Russia and Australia, for location categories ‘retail and recreation’, ‘grocery and pharmacy’, ‘workplace’ and ‘transit’ activity. This indicates that as disease incidence rose, activity levels declined. However, weaker, or even positive correlations, can be observed for these categories for countries across South America, in Eastern Europe and for India. The reverse pattern was observed for ‘parks’ and ‘residential’ categories; at these locations, an increase in activity would be expected if there was an increase in time spent close to habitation.

Distinct geographical patterns are noticeable from the map, with broad trends being apparent across large geographical areas. Thus, as well as on a country-wide basis, results were collated and examined on a continent-wide basis, as provided in Tables 1 and 2. As illustrated in the accompanying boxplots, when aggregating data this way, negative correlations between ‘retail and recreation’, ‘grocery and pharmacy’, ‘workplace’ and ‘transit’ activity with disease incidence occurred across all continents, except South America. Again, the opposite was observed for ‘parks’ and ‘residential’ activity.

**Fig. 2.**
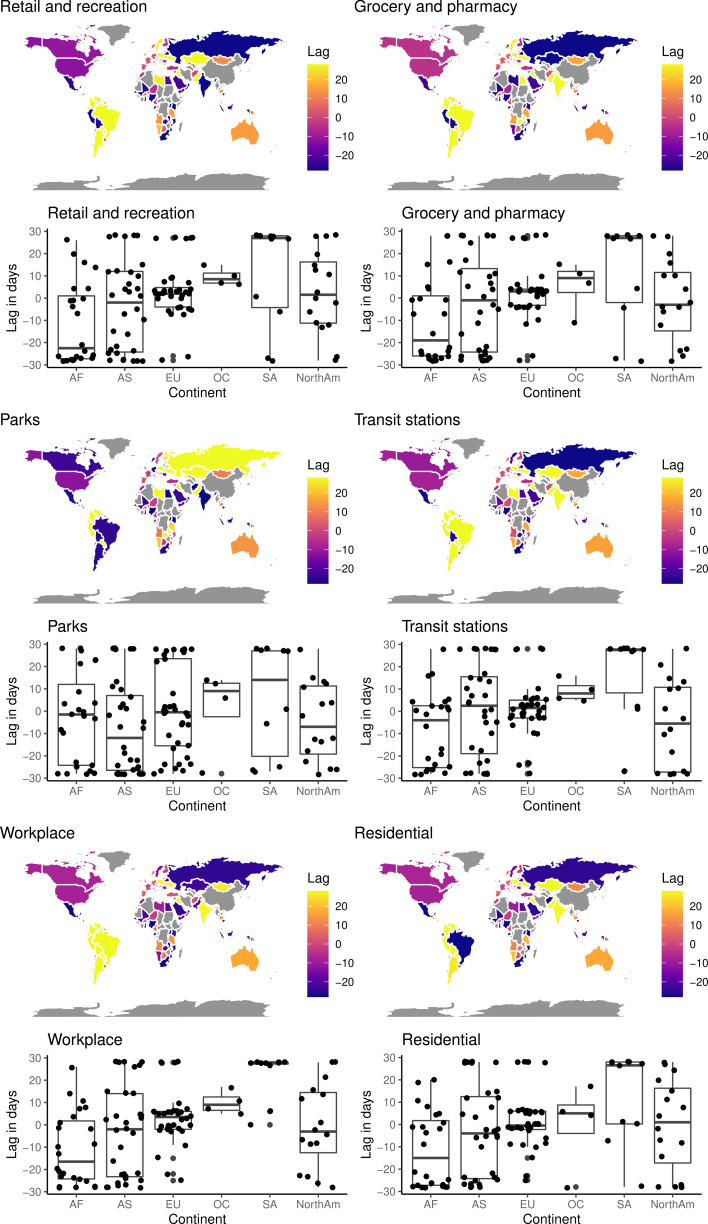
Lags to maximum correlations. Amount of time lagging in days resulting in the maximum Kendall's *τ* between COVID-19 for confirmed case number and measures of community activity (colour online only). Interesting are that the strongest correlations were when case numbers were negatively lagged by amounts of −20 days or greater for large areas across North America, Western Europe, Central Asia and Russia for the four categories indicative of mobility. This suggests that reductions in mobility in such areas occurred substantially prior to corresponding increases in COVID-19 case numbers. This is thus likely to have been substantially prior to formal legislation imposing movement restrictions coming into place. This indicated that personal behavioural choices and perceived risk perception may have played a greater role in driving movement patterns than legal restrictions.

Figure 2 shows the number of days of lagging, which resulted in the strongest correlation for each location category, for each individual country. Considerable negative time lags result in the strongest correlations for prominent countries across all categories, including the USA, Canada and Russia, and countries of Western Europe. The exception to this pattern is for ‘parks’, where a positive lag results in the strongest correlation for Russia.

Conversely, the strongest correlations are obtained with positive time lags for countries of South America and for Australia across all categories. The exception is ‘parks’ activity for countries of South America where negative lagging results in the strongest correlations; this is apparent from the accompanying boxplots which aggregate data continent wise. Significant differences were found for all six parameters (the lags in days producing the strongest correlation, and the *τ* value representing the correlation for all six CMR categories (Table 1 and 2)).

Clustering analysis was used to group countries on the basis of maximum absolute *τ* values and the corresponding lags. This identified four groups; the first where low lags resulted in greatest correlation (mainly Asian and Eastern European; e.g. India, Pakistan, Afghanistan, Russia, Belarus; but Brazil and Sweden also belong to this group). These countries showed weak levels of correlation, obtained with lags of around 0 or only weakly positive (median lags 3–10 days).

The second cluster contained mainly industrialised westernised countries such as the UK, Germany, Italy, Spain, the USA, Canada and Australia. This cluster had strong negative *τ* values, obtained using lags of only a few days (median −2 to 3.5). Group three (typically African and Asian countries; e.g. South Africa, Nigeria, Saudi Arabia, but also Mexico) had strong negative correlations obtained with strong negative lags (median −27 to −28). The final group (Eastern European and South American countries, such as Ukraine, Poland, Venezuela, Colombia) had strong positive correlations, and a high number of positive lags (27–28 days median). Detailed country-level values and cluster-level summary are provided in Supplementary material S2 and S3.

Pairwise comparisons with multiple testing correction found significant differences between Africa and South America in the lag producing the maximum correlation for ‘workplace’ data (*Z*: −4.74; adjusted *P*-value: <0.001) and between the Kendall's *τ* value for ‘retail and recreation’ between Africa and EU (*Z*: 4.049, adjusted *P*-value: 0.0045), the Kendall's *τ* value for ‘transit’ stations between EU and South America (*Z*:−4.253, adjusted *P*-value: 0.0019), and the Kendall's *τ* value for ‘residential’ data between the EU and South America (*Z*: 3.55, adjusted *P*-value: <0.001).

*Modelling:* The contemporaneous CMR-expanded model proved superior (BIC: 79096.29) to the numerical date-only model (BIC: 79802.47) and expanded model with distributed lag CMR (BIC: 80286.14). In the contemporaneous CMR-expanded model, the significant independent fixed-effects covariates were the numerical date and ‘retail and recreation’ (negative estimate) change compared with baseline mobility data. Summaries of the contemporaneous CMR-expanded model are shown in Table 3.

**Table 3.** Model summaries

Smoothed fixed-effect predictors	Estimate	*Standard error*	*P*-value
Date	14.88	0.67	<0.001
Retail and recreation percent change from baseline	−1.34	0.59	0.025
Grocery and pharmacy percent change from baseline	0.33	0.36	0.358
Transit stations percent change from baseline	−0.33	0.36	0.368
Workplaces percent change from baseline	−0.42	0.51	0.415
Standard deviation of the random intercept term	2.151		

Model summary statistics using contemporaneous CMR data.

When we validated the models, the results showed a somewhat different hierarchy of the models than based on the previous quality measures: the best performing model was the one with all variables with distributed lags (RMSE:6690.44). This was followed by the model without distributed lags (RMSE:6794.99). The worst predictive performance was identified with the model without the mobility data (RMSE:6840.16). Difference in predictive performance was significant between all models (pairwise two-sided Diebold–Mariano test, in all comparisons *P* < 0.001).

## Discussion

Here, clear correlations between COVID-19 case incidence and levels of mobility, as represented by Google's CMR data, were found. Distinct patterns were discernible over broad geographical areas, with clear negative relationships between disease occurrence and mobility being particularly apparent across North America, Western Europe, Russia and Australia. Reductions in those CMR categories indicating levels of social activity and mobility, ‘retail and recreation’, ‘grocery and pharmacy’, ‘workplace’ and ‘transit’ were apparent in these areas as COVID-19 case incidence rose. ‘Parks’ and ‘residential’ activity increased in line with COVID-19 incidence, suggesting increased time spent in a location close to home as case numbers rose. Thus, as COVID-19 epidemics developed, levels of mobility and movement declined.

That the relationship is clear for these areas may be related to the progression of the COVID-19 epidemic globally. COVID-19 was first identified in China, before spreading to Western European and North America; it is in these areas where the expected negative correlations are strongest and most apparent [35]. The results from clustering analysis, with the identification of distinct groups of countries, illustrate that similar patterns are observed by countries close together, or situated on the same continent. This lends support to the idea that patterns in disease progression occurred on a continent-wide level. The broad similarities apparent over continents also support this idea.

Another explanation for these patterns is that country-specific socio-economic or cultural factors may be influencing mobility levels in response to the disease outbreak. Although a generalisation, those countries where strongly negative correlations were most apparent, are those often categorised as being ‘developed’ nations; they possess well-established communication and media outlets, effective governmental control and host populations possibly more compliant with governmental restrictions. Knowledge and understanding about COVID-19 may have been more widely disseminated and accessible in these countries, influencing personal perceptions of risk, and thus personal movement decisions.

The results from the cross-correlation analyses suggest that mobility reductions occurred sometime prior to corresponding increases in COVID-19 cases in many countries where negative correlations were observed. This may be because of delays in disease identification and reporting which meant that data on confirmed cases did not reflect actual disease incidence. It may be related to the spread of COVID-19 globally, and similar patterns may become more apparent for other areas of the globe once the full progression of the COVID-19 epidemic is understood; further examination at a later date may prove productive. However, these patterns in lagging could also be related to population attributes of those countries. Other studies have found that behavioural characteristics shared by those in nation states influence attitude to risk and subsequent behaviour [34]. Those populations at where the strongest negative correlations are observed may be more risk averse.

Although legally enforced restrictions on mobility are the most obvious factor causing a reduction in population mobility, potentially more important are personal behavioural choices made in response to the threat posed by infectious disease [36, 37]. For example, surveying of Americans found they avoided public gatherings, such as sporting events, malls and public transport in response to fears of H1N1 [38]. Following the SARS epidemic of 2002, large reductions in travel into and out of Hong Kong are reported [15]. Surveys found a reluctance to travel and engage in social activities in communities where there was a perceived risk from SARS infection [36].

Studies examining personal risk perception and its relationship with movement during the COVID-19 outbreak show that personal behavioural and psychological traits may be important in influencing the levels of mobility. Chan *et al*. studied nationwide personality traits along with Google CMR data, finding that countries with agreeable and conscientious personality traits showed greater levels of mobility reduction than countries exhibiting more openness [34]. Another study examined survey data on nationwide risk-taking attitudes, finding that this affected mobility; it also reported a clear effect on the mobility of the WHO pandemic announcement [33]. In another work, the importance of freedom of assembly and association was identified as the most important predictor of COVID-19 doubling time among cultural norms [26]. The factors affecting personal movement decisions are complex and inter-related; including the personal perceived risk of infection, socio-economic factors and peer group behaviour [27].

The examination of mobility and disease occurrence, and its use in disease modelling has been examined for recent disease epidemics. The 2009 H1N1 influenza pandemic provides a good recent parallel for the current COVID-19 pandemic. Much of the H1N1 infection was spread globally through international air travel [39, 40]. Modelling studies have shown how human movement and mobility patterns influenced the geographic spread and timing of this epidemic [41, 42]. Bajardi *et al*. modelled disease spread using travel data, concluding that restrictions on international travel played little role in controlling the spread of this epidemic globally [43].

The potential use of mobility data, and its potential use in COVID-19 modelling, was identified early [44]. Oliver *et al*. review the potential use of mobile phone data in COVID-19 modelling [45]. Studies using such data are rapidly appearing. Notable studies include Jia *et al*. who used data collated from mobile phone records showing population outflows from Wuhan, to assess the impact quarantining had on mobility, and to predict the frequency and distribution of COVID-19 infections across China [46]. Another recent study modelled the spread of COVID-19 using mobile phone data, modelling the effect of different movement control measures on COVID-19 incidence [47]. Kraemer *et al*. used travel-related data from the website Baidu to show patterns in COVID-19 establishment; a relationship between the frequency of travel out of Wuhan accounted for patterns in disease across China [32]. Non-Chinese-based studies include that of Badr *et al*., which modelled the relationship between mobility and confirmed case numbers for individual US counties, and related these to state-wide restrictions on movement [48]. In Italy, Bonaccorsi *et al*. examined the economic effect of mobility restrictions finding that the mobility effects were greater in areas with greater fiscal capacity. In contrast to other studies [49], Chinazzi *et al*., who studied the travel restrictions in China, concluded that they played little effect in halting the spread of the infection [50]. Many of these studies examine mobility patterns in individual countries; however, Google's CMR is also being used to model COVID-19 across broader geographical ranges. Zhu *et al*. have used CMR to gauge future case numbers and the reproductive number of COVID-19 across South American countries [51].

Epidemiologists are constantly seeking new sources of data sources with the potential to enhance existing disease forecasting and modelling. The initial attempts made here to use data from Google's CMR in disease modelling are promising. Integrating contemporaneous mobility data into models of case incidence resulted in models providing better quality measures than those utilising lagged-distributed data. Other similar datasets, such as Apple's Mobility Trends data [52], could be examined and compared to see if similar relationships as found here are apparent. Particularly interesting is that Google's CMR could also offer the potential to examine movement at the local scale rather, meaning more precise and locally based understanding of disease dynamics could be gained.

A disadvantage of using Google's CMR is that the data do not directly equate to some COVID-19 control measures. For example, ‘social distancing’ has been widely promoted as a measure to reduce the transmission of COVID-19 [53]. Valenti *et al*. [54] used CMR data as an estimate of social distancing in the modelling of deaths in Brazil. However, CMR data indicate only general activity at specific location types, and provide no direct indication of adherence to such rules. Another disadvantage is that users of Google technology may not be representative of a country's population as a whole. Demographic groups particularly affected by COVID-19, such as the elderly, who may be more cautious, may be underrepresented in such data. Age, economic and sexual differences in the make-up of Google users may occur between countries, and thus affect country comparisons. One of the disadvantages of this study is that such country-specific reasons are not considered; such research requires further study and consideration of other socio-economic, behavioural and psychological factors. The method used here, examining associations between mobility and case number, provides no insight into the causative factors driving such patterns.

A strength of this study was that it examined broader global trends using CMR. Distinct patterns were observed by examining data on a continent-level scale; most studies examine mobility data only at a national scale, meaning such patterns may be missed. Despite political wish thinking, the spread of infectious disease occurs regardless of notional national boundaries, meaning such examination is pertinent. The format of data provided by CMR means easy cross-country comparison is possible. Another strength of the study was that the modelling method used extended that of previous work; smoothing of variables was applied with GAM modelling to deal with non-linearities. Lagging was made by applying spline-described values to achieve a distributed lag model instead of adding each and every lagged value to reduce complexity and to avoid overfitting.

As already highlighted, a limitation here is that the reasons underlying the patterns observed in mobility were not examined. No attempt to examine the sociological issues affecting mobility was made. Chan *et al*. [33] note that reductions in mobility coincided with the WHO announcement of a global pandemic in January 2020; further modelling could integrate this into future work. Individual country correlations were not compared directly with each other. Further work could also examine the timing of disease progression in each country, relating it directly to actual changes in activity data. We speculated that economic development status may account for patterns observed here; examination of GDP data, which reflects such status, and its relationship with mobility may be of interest.

In summary, a relationship between levels of social activity and community movement with disease incidence was found for countries globally using Google's CMR. As COVID-19 became established globally, levels of mobility declined, this may be either because of government recommendations and imposed legal restrictions, or through personal behavioural changes resulting from fear of disease. Google's CMR illustrates the effect these measures had on community movement. Interesting is that reductions in mobility appeared to occur substantially prior to the implementation of legal restrictions on movement in many countries, suggesting the importance of personal perceived risk of infection and personal behavioural modifications rather than government edict. Further study to ascertain how countries differ in their adherence to such measures, and whether this is apparent in CMR, would be most interesting. An understanding of the cultural, social and economic factors possibly accounting for some of the differences observed between countries could be productive.

In conclusion, the observed relationship between CMR data and case incidence, and its ability to enhance model quality and prediction suggests data related to community mobility could prove of use in future COVID-19 modelling.

## Data Availability

Publicly available sources of data were used. Google Community Mobility Reports: https://www.google.com/covid19/mobility/. Data on case incidence: John Hopkins University, Center for Systems Science and Engineering (CSSE), accessed via github: https://github.com/datasets/covid-19. Data are also available under the following link: https://github.com/msulyok/GoogleMobilityDataCOVID

## Supplementary material

For supplementary material accompanying this paper visit https://doi.org/10.1017/S0950268820002757. click here to view supplementary material
