# Supplementary material for: Community movement and COVID-19: a global study using Google's Community Mobility Reports
Source: Epidemiol Infect. 2020 Nov 13;148:e284. doi: 10.1017/S0950268820002757 (PMC7729173; doi:10.1017/S0950268820002757)
Supplement: Supplementary file 1 [file S0950268820002757sup001.docx]

Epidemiology and Infection

Community Movement and COVID-19: A global study using Google's Community Mobility Reports

M. SULYOK, M. WALKER

Supplementary Material

**S1: Results of Kendall’s τ correlations, time lag (days) resulting in strongest correlation for individual countries and results of clustering.**

|  | country | Continent | Retail and recreation | Grocery and pharmacy | Parks | Transit stations | Workplaces | Residential | Retail and recreation | | Grocery and pharmacy | Parks | Transit stations | Workplaces | Residential | Cluster | |
| --- | --- | --- | --- | --- | --- | --- | --- | --- | --- | --- | --- | --- | --- | --- | --- | --- | --- |
|  |  |  | Lag in days | | | | | | | Tau value at maximal correlation | | | | | | |  |
| 1 | Afghanistan | AS | 1 | 4 | -28 | 1 | 1 | -28 | -0.553 | | -0.514 | -0.697 | -0.573 | -0.513 | 0.630 | 1 | |
| 2 | Angola | AF | 14 | 15 | 9 | 2 | 11 | 11 | -0.158 | | -0.129 | -0.141 | -0.177 | -0.165 | 0.148 | 2 | |
| 3 | Antigua and Barbuda | NorthAm | 11 | 10 | 11 | 10 | 21 | 24 | -0.250 | | -0.273 | -0.282 | -0.238 | -0.253 | 0.343 | 2 | |
| 4 | Argentina | SA | 28 | 28 | -26 | 28 | 28 | 26 | 0.343 | | 0.360 | -0.547 | 0.392 | 0.459 | -0.450 | 1 | |
| 5 | Australia | OC | 15 | 15 | 14 | 16 | 17 | 17 | -0.684 | | -0.560 | -0.575 | -0.706 | -0.694 | 0.676 | 2 | |
| 6 | Austria | EU | 3 | 3 | -1 | 3 | 5 | 5 | -0.730 | | -0.583 | -0.538 | -0.765 | -0.691 | 0.696 | 2 | |
| 7 | Bahrain | AS | -28 | -28 | -28 | -28 | -28 | -28 | -0.431 | | -0.399 | -0.393 | -0.412 | -0.456 | 0.659 | 3 | |
| 8 | Bangladesh | AS | -28 | -27 | -28 | -28 | -22 | -28 | -0.417 | | -0.409 | -0.623 | -0.404 | -0.384 | 0.439 | 3 | |
| 9 | Barbados | NorthAm | 15 | 16 | 12 | 15 | 12 | 11 | -0.467 | | -0.463 | -0.452 | -0.478 | -0.468 | 0.478 | 2 | |
| 10 | Belarus | EU | -26 | -26 | 25 | -24 | -22 | -23 | -0.717 | | -0.557 | 0.405 | -0.749 | -0.690 | 0.556 | 1 | |
| 11 | Belgium | EU | -6 | 1 | -6 | -2 | -3 | -2 | -0.750 | | -0.605 | -0.384 | -0.773 | -0.671 | 0.725 | 2 | |
| 12 | Belize | NorthAm | 13 | -24 | 13 | 13 | -26 | 15 | -0.376 | | 0.397 | -0.357 | -0.391 | 0.343 | 0.345 | 1 | |
| 13 | Benin | AF | -26 | -22 | 23 | -25 | -25 | -27 | -0.171 | | -0.183 | -0.207 | -0.216 | -0.145 | 0.201 | 1 | |
| 14 | Bolivia | SA | -27 | -27 | -27 | -27 | 28 | 28 | -0.373 | | -0.397 | -0.372 | -0.401 | 0.471 | -0.399 | 1 | |
| 15 | Bosnia and Herzegovina | EU | -4 | -3 | 28 | -3 | -1 | -8 | -0.468 | | -0.375 | 0.369 | -0.496 | -0.490 | 0.487 | 1 | |
| 16 | Botswana | AF | 4 | 5 | -3 | 5 | 8 | 4 | -0.146 | | -0.099 | 0.104 | -0.169 | -0.155 | 0.193 | 2 | |
| 17 | Brazil | SA | 28 | 28 | -25 | 28 | 28 | -28 | 0.386 | | 0.366 | -0.434 | 0.466 | 0.360 | 0.370 | 1 | |
| 18 | Bulgaria | EU | 27 | 27 | 26 | 28 | 28 | 28 | 0.547 | | 0.509 | 0.504 | 0.481 | 0.378 | -0.445 | 4 | |
| 19 | Burkina Faso | AF | 4 | 4 | 4 | 4 | 1 | 1 | -0.552 | | -0.519 | -0.490 | -0.603 | -0.447 | 0.591 | 2 | |
| 20 | Cambodia | AS | -24 | -24 | -28 | -28 | -27 | -25 | 0.504 | | 0.512 | 0.484 | 0.489 | 0.521 | -0.518 | 1 | |
| 21 | Cameroon | AF | -1 | -1 | -1 | -1 | 7 | -1 | -0.218 | | -0.215 | -0.278 | -0.212 | -0.207 | 0.244 | 2 | |
| 22 | Canada | NorthAm | -11 | -4 | -23 | -10 | -7 | -7 | -0.722 | | -0.614 | -0.475 | -0.730 | -0.631 | 0.656 | 2 | |
| 23 | Chile | SA | -6 | -4 | -6 | 2 | 0 | 0 | -0.484 | | -0.541 | -0.586 | -0.613 | -0.400 | 0.556 | 2 | |
| 24 | Colombia | SA | 28 | 28 | 28 | 28 | 28 | 28 | 0.512 | | 0.439 | 0.488 | 0.578 | 0.491 | -0.429 | 4 | |
| 25 | Costa Rica | NorthAm | 3 | 10 | 3 | 10 | 14 | 8 | -0.381 | | -0.385 | -0.423 | -0.433 | -0.407 | 0.417 | 2 | |
| 26 | Croatia | EU | 2 | 4 | 2 | 3 | 2 | 0 | -0.687 | | -0.553 | -0.504 | -0.709 | -0.659 | 0.657 | 2 | |
| 27 | Czechia | EU | 1 | 3 | 22 | -3 | 0 | -1 | -0.596 | | -0.418 | 0.250 | -0.596 | -0.571 | 0.574 | 1 | |
| 28 | Denmark | EU | 2 | -12 | -24 | 2 | 6 | -1 | -0.479 | | -0.419 | -0.264 | -0.636 | -0.509 | 0.549 | 2 | |
| 29 | Dominican Republic | NorthAm | 28 | 28 | 28 | 28 | 28 | 28 | 0.485 | | 0.443 | 0.469 | 0.463 | 0.485 | -0.429 | 4 | |
| 30 | Ecuador | SA | 28 | 28 | 28 | 28 | 28 | -7 | 0.325 | | 0.267 | 0.325 | 0.349 | 0.320 | 0.283 | 1 | |
| 31 | Egypt | AF | -21 | 28 | -28 | -21 | -20 | -21 | -0.417 | | 0.336 | -0.393 | -0.350 | -0.330 | 0.391 | 1 | |
| 32 | El Salvador | NorthAm | -28 | -28 | -28 | -28 | -23 | -28 | -0.512 | | -0.501 | -0.535 | -0.590 | -0.423 | 0.489 | 3 | |
| 33 | Estonia | EU | 1 | 3 | -24 | -1 | 6 | -1 | -0.602 | | -0.499 | -0.240 | -0.614 | -0.534 | 0.566 | 2 | |
| 34 | Fiji | OC | 6 | 7 | -28 | 6 | 11 | -28 | -0.371 | | -0.367 | 0.350 | -0.359 | -0.342 | -0.362 | 1 | |
| 35 | Finland | EU | -4 | -4 | 27 | -1 | -2 | -3 | -0.651 | | -0.471 | 0.327 | -0.662 | -0.455 | 0.569 | 1 | |
| 36 | France | EU | 2 | 4 | -2 | 3 | 0 | 0 | -0.601 | | -0.524 | -0.466 | -0.598 | -0.488 | 0.533 | 2 | |
| 37 | Gabon | AF | -24 | -28 | -10 | -19 | -28 | -28 | -0.344 | | -0.351 | -0.334 | -0.322 | -0.493 | 0.464 | 3 | |
| 38 | Georgia | AS | -7 | -6 | -7 | 4 | 4 | -6 | -0.556 | | -0.513 | -0.512 | -0.568 | -0.529 | 0.502 | 2 | |
| 39 | Germany | EU | 2 | 6 | -26 | 2 | 6 | 6 | -0.731 | | -0.411 | -0.325 | -0.756 | -0.590 | 0.664 | 2 | |
| 40 | Ghana | AF | -26 | -26 | -23 | -26 | -23 | -23 | -0.222 | | -0.213 | -0.239 | -0.231 | -0.185 | 0.270 | 3 | |
| 41 | Greece | EU | 9 | 10 | -6 | 10 | 10 | 6 | -0.548 | | -0.346 | -0.353 | -0.565 | -0.520 | 0.544 | 2 | |
| 42 | Guatemala | NorthAm | 28 | -26 | -26 | -28 | -23 | -28 | 0.320 | | -0.380 | -0.383 | -0.314 | -0.327 | 0.384 | 1 | |
| 43 | Haiti | NorthAm | -13 | -12 | -13 | -28 | -9 | 4 | -0.324 | | -0.278 | -0.352 | -0.290 | -0.258 | 0.311 | 1 | |
| 44 | Honduras | NorthAm | 28 | 28 | -18 | -18 | 28 | 27 | 0.381 | | 0.273 | -0.273 | -0.322 | 0.482 | -0.374 | 1 | |
| 45 | Hungary | EU | -5 | 2 | -17 | -2 | -2 | -9 | -0.670 | | -0.518 | -0.483 | -0.661 | -0.616 | 0.606 | 2 | |
| 46 | India | AS | -28 | 28 | -28 | 28 | 28 | 28 | -0.583 | | 0.598 | -0.652 | 0.566 | 0.444 | -0.418 | 1 | |
| 47 | Indonesia | AS | -25 | -25 | -26 | -18 | -22 | -22 | -0.492 | | -0.337 | -0.495 | -0.550 | -0.571 | 0.615 | 3 | |
| 48 | Iraq | AS | 28 | 28 | 28 | 28 | 28 | 28 | 0.362 | | 0.327 | 0.334 | 0.325 | 0.316 | -0.304 | 4 | |
| 49 | Ireland | EU | 7 | 4 | 8 | -2 | -2 | -2 | -0.778 | | -0.649 | -0.223 | -0.741 | -0.640 | 0.639 | 1 | |
| 50 | Israel | AS | 3 | 5 | -2 | -2 | -2 | -2 | -0.571 | | -0.408 | -0.531 | -0.603 | -0.523 | 0.529 | 2 | |
| 51 | Italy | EU | 7 | 3 | 2 | 3 | 5 | 5 | -0.798 | | -0.662 | -0.739 | -0.801 | -0.791 | 0.812 | 2 | |
| 52 | Jamaica | NorthAm | 0 | 4 | 4 | -3 | -2 | -2 | -0.404 | | -0.409 | -0.431 | -0.428 | -0.421 | 0.467 | 2 | |
| 53 | Japan | AS | 15 | 25 | -16 | 15 | 14 | 14 | -0.678 | | -0.372 | 0.291 | -0.688 | -0.736 | 0.680 | 1 | |
| 54 | Jordan | AS | 4 | 4 | 3 | 4 | 3 | 3 | -0.343 | | -0.275 | -0.337 | -0.369 | -0.395 | 0.367 | 2 | |
| 55 | Kazakhstan | AS | 28 | -28 | 28 | 28 | -23 | 28 | 0.358 | | -0.330 | 0.436 | 0.354 | -0.331 | -0.368 | 1 | |
| 56 | Kenya | AF | -28 | -24 | -28 | -27 | -24 | -28 | -0.403 | | -0.389 | -0.461 | -0.401 | -0.316 | 0.444 | 3 | |
| 57 | Kuwait | AS | -15 | 1 | 1 | 0 | -4 | -3 | -0.725 | | -0.739 | -0.693 | -0.682 | -0.714 | 0.720 | 2 | |
| 58 | Kyrgyzstan | AS | 28 | 28 | 28 | 28 | 28 | 28 | 0.387 | | 0.361 | 0.410 | 0.326 | 0.314 | -0.374 | 4 | |
| 59 | Laos | AS | 10 | 11 | 11 | 10 | -26 | 8 | -0.412 | | -0.420 | -0.382 | -0.404 | 0.435 | 0.413 | 1 | |
| 60 | Latvia | EU | 3 | 3 | -27 | 5 | 5 | 5 | -0.643 | | -0.509 | -0.209 | -0.635 | -0.579 | 0.568 | 2 | |
| 61 | Lebanon | AS | 4 | -22 | -28 | 7 | 5 | -24 | -0.298 | | 0.314 | 0.295 | -0.351 | -0.303 | -0.309 | 1 | |
| 62 | Libya | AF | 26 | -26 | 28 | 28 | -9 | -9 | 0.209 | | 0.303 | 0.216 | 0.236 | -0.166 | 0.145 | 1 | |
| 63 | Lithuania | EU | 0 | -3 | -28 | -3 | -2 | -2 | -0.696 | | -0.434 | -0.231 | -0.704 | -0.567 | 0.572 | 2 | |
| 64 | Luxembourg | EU | 4 | 4 | -1 | 5 | 6 | 6 | -0.739 | | -0.654 | -0.481 | -0.767 | -0.725 | 0.741 | 2 | |
| 65 | Malaysia | AS | 6 | 7 | 6 | 7 | 4 | 5 | -0.599 | | -0.593 | -0.631 | -0.656 | -0.649 | 0.664 | 2 | |
| 66 | Mali | AF | -27 | -25 | -25 | -17 | -22 | -26 | -0.486 | | -0.452 | -0.388 | -0.580 | -0.435 | 0.506 | 3 | |
| 67 | Malta | EU | 3 | 3 | 0 | 3 | 3 | 0 | -0.537 | | -0.505 | -0.473 | -0.550 | -0.524 | 0.510 | 2 | |
| 68 | Mauritius | AF | 0 | 0 | 3 | 4 | 4 | 5 | -0.484 | | -0.431 | -0.480 | -0.504 | -0.510 | 0.485 | 2 | |
| 69 | Mexico | NorthAm | -26 | -23 | -26 | -28 | -28 | -28 | -0.638 | | -0.566 | -0.686 | -0.728 | -0.535 | 0.681 | 3 | |
| 70 | Moldova | EU | 27 | 28 | 27 | 28 | 28 | 28 | 0.595 | | 0.553 | 0.594 | 0.509 | 0.442 | -0.486 | 4 | |
| 71 | Mongolia | AS | 12 | 17 | 10 | 17 | 26 | 12 | 0.285 | | 0.279 | 0.303 | 0.301 | 0.327 | -0.296 | 1 | |
| 72 | Morocco | AF | -7 | -7 | -8 | -7 | -10 | -4 | -0.553 | | -0.531 | -0.581 | -0.593 | -0.570 | 0.646 | 2 | |
| 73 | Mozambique | AF | -28 | -28 | 0 | 0 | -24 | -25 | -0.320 | | -0.173 | -0.426 | -0.299 | -0.277 | 0.309 | 1 | |
| 74 | Namibia | AF | 20 | -16 | 21 | 17 | -13 | 20 | -0.243 | | 0.323 | -0.262 | -0.232 | 0.223 | 0.223 | 1 | |
| 75 | Nepal | AS | 28 | 28 | 28 | 28 | 28 | 28 | 0.340 | | 0.365 | 0.337 | 0.356 | 0.322 | -0.329 | 4 | |
| 76 | Netherlands | EU | 0 | 3 | -27 | -3 | -3 | -2 | -0.726 | | -0.535 | -0.399 | -0.696 | -0.518 | 0.640 | 2 | |
| 77 | New Zealand | OC | 10 | 11 | 6 | 10 | 7 | 6 | -0.653 | | -0.579 | -0.623 | -0.669 | -0.659 | 0.640 | 2 | |
| 78 | Nicaragua | NorthAm | -2 | -2 | -2 | 21 | 1 | 20 | -0.237 | | -0.229 | -0.197 | -0.220 | -0.193 | 0.182 | 1 | |
| 79 | Niger | AF | -4 | -7 | -2 | 1 | -2 | -2 | -0.594 | | -0.457 | -0.571 | -0.582 | -0.493 | 0.594 | 2 | |
| 80 | Nigeria | AF | -28 | -27 | -28 | -28 | -28 | -28 | -0.473 | | -0.422 | -0.506 | -0.440 | -0.455 | 0.538 | 3 | |
| 81 | North Macedonia | EU | -7 | -4 | 28 | -6 | 4 | -6 | -0.308 | | -0.270 | 0.354 | -0.330 | -0.315 | 0.315 | 1 | |
| 82 | Norway | EU | 3 | -4 | -24 | 6 | 7 | 7 | -0.576 | | -0.313 | -0.456 | -0.685 | -0.581 | 0.626 | 2 | |
| 83 | Oman | AS | -28 | -26 | -22 | -28 | -24 | -24 | -0.407 | | -0.435 | -0.519 | -0.438 | -0.456 | 0.627 | 3 | |
| 84 | Pakistan | AS | 28 | 28 | 28 | 28 | -22 | 28 | 0.421 | | 0.386 | 0.372 | 0.401 | -0.300 | -0.356 | 1 | |
| 85 | Panama | NorthAm | -6 | -6 | -14 | 1 | -4 | -14 | -0.351 | | -0.352 | -0.352 | -0.345 | -0.248 | 0.320 | 2 | |
| 86 | Papua New Guinea | OC | 7 | -11 | 12 | 5 | 5 | 4 | -0.208 | | -0.191 | -0.216 | -0.185 | -0.198 | 0.203 | 1 | |
| 87 | Paraguay | SA | 27 | 27 | 27 | 27 | 28 | 27 | 0.374 | | 0.362 | 0.322 | 0.327 | 0.311 | -0.317 | 4 | |
| 88 | Peru | SA | -28 | -28 | 27 | 27 | 27 | 28 | -0.449 | | -0.410 | 0.473 | 0.484 | 0.523 | -0.471 | 1 | |
| 89 | Philippines | AS | -25 | 28 | -26 | 28 | 27 | -6 | -0.344 | | 0.228 | -0.404 | 0.267 | 0.274 | 0.309 | 1 | |
| 90 | Poland | EU | 27 | 27 | 28 | 28 | 28 | 28 | 0.507 | | 0.434 | 0.487 | 0.500 | 0.359 | -0.461 | 4 | |
| 91 | Portugal | EU | -5 | -4 | -5 | 1 | -1 | -1 | -0.597 | | -0.539 | -0.498 | -0.593 | -0.553 | 0.567 | 2 | |
| 92 | Qatar | AS | -28 | -28 | -28 | -28 | -28 | -27 | -0.585 | | -0.530 | -0.607 | -0.618 | -0.681 | 0.694 | 3 | |
| 93 | Romania | EU | -4 | -4 | -15 | -10 | -9 | -9 | -0.592 | | -0.576 | -0.503 | -0.612 | -0.602 | 0.599 | 2 | |
| 94 | Russia | EU | -28 | -28 | 28 | -28 | -25 | -25 | -0.662 | | -0.591 | 0.528 | -0.646 | -0.542 | 0.558 | 1 | |
| 95 | Rwanda | AF | -1 | -1 | 1 | 2 | -1 | -1 | -0.284 | | -0.277 | -0.278 | -0.262 | -0.253 | 0.287 | 2 | |
| 96 | Saudi Arabia | AS | -23 | -23 | -24 | -23 | -25 | -25 | -0.555 | | -0.549 | -0.637 | -0.623 | -0.628 | 0.662 | 3 | |
| 97 | Senegal | AF | -28 | -24 | -28 | -24 | -28 | -28 | -0.477 | | -0.486 | -0.593 | -0.527 | -0.418 | 0.542 | 3 | |
| 98 | Serbia | EU | -7 | -10 | -14 | -10 | -9 | -10 | -0.641 | | -0.511 | -0.555 | -0.651 | -0.631 | 0.588 | 2 | |
| 99 | Singapore | AS | 1 | -3 | 2 | -5 | -2 | -2 | -0.754 | | -0.497 | -0.752 | -0.748 | -0.716 | 0.711 | 2 | |
| 100 | Slovakia | EU | 3 | 3 | -21 | 1 | 6 | -1 | -0.618 | | -0.415 | -0.427 | -0.601 | -0.583 | 0.565 | 2 | |
| 101 | Slovenia | EU | 4 | 4 | 1 | 6 | 6 | 6 | -0.625 | | -0.545 | -0.446 | -0.678 | -0.683 | 0.657 | 2 | |
| 102 | South Africa | AF | -28 | -28 | -27 | -28 | -28 | -28 | -0.294 | | -0.260 | -0.460 | -0.345 | -0.361 | 0.402 | 3 | |
| 103 | Spain | EU | 2 | 2 | 2 | 3 | 6 | 6 | -0.742 | | -0.686 | -0.702 | -0.752 | -0.726 | 0.699 | 2 | |
| 104 | Sri Lanka | AS | -22 | -28 | -22 | -22 | -27 | -27 | -0.402 | | -0.396 | -0.402 | -0.401 | -0.434 | 0.439 | 3 | |
| 105 | Sweden | EU | 27 | 27 | 0 | -23 | -15 | -15 | 0.492 | | 0.329 | 0.548 | -0.378 | -0.463 | 0.476 | 1 | |
| 106 | Switzerland | EU | 9 | 4 | 23 | 6 | 6 | 6 | -0.761 | | -0.387 | 0.228 | -0.703 | -0.666 | 0.672 | 1 | |
| 107 | Tajikistan | AS | -10 | -11 | -8 | -10 | -10 | -10 | -0.721 | | -0.696 | -0.694 | -0.730 | -0.673 | 0.730 | 2 | |
| 108 | Tanzania | AF | 16 | 16 | 23 | 16 | 14 | 19 | -0.390 | | -0.395 | -0.392 | -0.398 | -0.370 | 0.406 | 2 | |
| 109 | Thailand | AS | 12 | 10 | 13 | 13 | 14 | 12 | -0.683 | | -0.591 | -0.652 | -0.717 | -0.570 | 0.682 | 2 | |
| 110 | Togo | AF | -27 | -27 | 28 | 2 | 26 | -27 | -0.348 | | -0.335 | 0.326 | -0.351 | 0.219 | 0.380 | 1 | |
| 111 | Trinidad and Tobago | NorthAm | 20 | 20 | 15 | -27 | 16 | -27 | -0.556 | | -0.514 | -0.549 | 0.533 | -0.493 | -0.513 | 1 | |
| 112 | Turkey | AS | -5 | -5 | -5 | -5 | 2 | -5 | -0.593 | | -0.290 | -0.483 | -0.620 | -0.644 | 0.621 | 2 | |
| 113 | Uganda | AF | -28 | -26 | -24 | -28 | -28 | -28 | -0.348 | | -0.345 | -0.508 | -0.405 | -0.337 | 0.388 | 3 | |
| 114 | Ukraine | EU | 27 | 28 | 28 | 28 | 28 | 28 | 0.552 | | 0.579 | 0.580 | 0.544 | 0.436 | -0.485 | 4 | |
| 115 | United Arab Emirates | AS | -28 | -26 | -28 | -28 | -28 | -27 | -0.641 | | -0.573 | -0.684 | -0.691 | -0.646 | 0.677 | 3 | |
| 116 | United Kingdom | EU | 2 | 4 | -11 | 0 | 0 | 0 | -0.759 | | -0.662 | -0.383 | -0.747 | -0.715 | 0.697 | 2 | |
| 117 | United States | NorthAm | -12 | -4 | -12 | -8 | -8 | -8 | -0.694 | | -0.577 | -0.374 | -0.690 | -0.680 | 0.695 | 2 | |
| 118 | Uruguay | SA | 1 | 4 | 1 | 1 | 6 | 1 | -0.677 | | -0.578 | -0.623 | -0.631 | -0.599 | 0.676 | 2 | |
| 119 | Venezuela | SA | 27 | 27 | 27 | 28 | 28 | 28 | 0.368 | | 0.314 | 0.338 | 0.383 | 0.318 | -0.302 | 4 | |
| 120 | Vietnam | AS | 12 | 12 | -25 | 14 | 15 | 15 | -0.549 | | -0.489 | 0.520 | -0.481 | -0.481 | 0.539 | 1 | |
| 121 | Yemen | AS | -16 | -16 | -19 | -16 | -16 | -5 | -0.376 | | -0.210 | -0.502 | -0.280 | -0.549 | 0.641 | 1 | |
| 122 | Zambia | AF | -26 | 28 | 27 | -26 | -21 | 8 | -0.283 | | 0.296 | 0.353 | -0.311 | -0.300 | 0.408 | 1 | |

**S2: Cluster level aggregated summaries and comparisons.**

| cluster | 1 | 2 | 3 | 4 | p |
| --- | --- | --- | --- | --- | --- |
| n | 41 | 52 | 18 | 11 |  |
| Continent (%) |  |  |  |  | 0.006 |
| AF | 7 ( 17.1) | 9 ( 17.3) | 8 ( 44.4) | 0 ( 0.0) |  |
| AS | 12 ( 29.3) | 9 ( 17.3) | 8 ( 44.4) | 3 ( 27.3) |  |
| EU | 9 ( 22.0) | 23 ( 44.2) | 0 ( 0.0) | 4 ( 36.4) |  |
| OC | 2 ( 4.9) | 2 ( 3.8) | 0 ( 0.0) | 0 ( 0.0) |  |
| SA | 5 ( 12.2) | 2 ( 3.8) | 0 ( 0.0) | 3 ( 27.3) |  |
| NorthAm | 6 ( 14.6) | 7 ( 13.5) | 2 ( 11.1) | 1 ( 9.1) |  |
| Retail and recreation-lag (median [IQR]) | 4.00 [-24.00, 20.00] | 2.00 [-4.25, 4.00] | -28.00 [-28.00, -26.00] | 27.00 [27.00, 28.00] | <0.001 |
| Grocery and pharmacy- lag (median [IQR]) | -2.00 [-24.00, 25.00] | 3.00 [-4.00, 4.25] | -26.00 [-28.00, -25.00] | 28.00 [27.00, 28.00] | <0.001 |
| Parks- lag (median [IQR]) | 10.00 [-25.00, 27.00] | -2.00 [-12.50, 2.25] | -26.50 [-28.00, -24.00] | 28.00 [27.00, 28.00] | <0.001 |
| Transit stations- lag (median [IQR]) | 2.00 [-21.00, 17.00] | 2.00 [-2.00, 5.00] | -28.00 [-28.00, -23.25] | 28.00 [28.00, 28.00] | <0.001 |
| Workplaces- lag (median [IQR]) | 0.00 [-21.00, 16.00] | 3.50 [-2.00, 6.00] | -27.50 [-28.00, -23.25] | 28.00 [28.00, 28.00] | <0.001 |
| Residential- lag (median [IQR]) | -3.00 [-24.00, 15.00] | 0.00 [-2.00, 6.00] | -28.00 [-28.00, -26.25] | 28.00 [28.00, 28.00] | <0.001 |
| Retail and recreation-Kendall`s τ (median [IQR]) | -0.34 [-0.55, 0.21] | -0.60 [-0.70, -0.48] | -0.45 [-0.51, -0.40] | 0.49 [0.37, 0.53] | <0.001 |
| Grocery and pharmacy- Kendall`s τ (median [IQR]) | -0.23 [-0.40, 0.30] | -0.51 [-0.58, -0.41] | -0.42 [-0.50, -0.36] | 0.42 [0.36, 0.48] | <0.001 |
| Parks- Kendall`s τ (median [IQR]) | 0.22 [-0.38, 0.35] | -0.47 [-0.55, -0.37] | -0.51 [-0.60, -0.42] | 0.47 [0.34, 0.50] | <0.001 |
| Transit stations- Kendall`s τ (median [IQR]) | -0.31 [-0.40, 0.27] | -0.63 [-0.70, -0.56] | -0.44 [-0.59, -0.40] | 0.46 [0.34, 0.50] | <0.001 |
| Workplaces- Kendall`s τ (median [IQR]) | -0.30 [-0.48, 0.32] | -0.57 [-0.66, -0.48] | -0.44 [-0.52, -0.39] | 0.36 [0.32, 0.44] | <0.001 |
| Residential- Kendall`s τ (median [IQR]) | 0.31 [-0.31, 0.49] | 0.59 [0.51, 0.67] | 0.52 [0.44, 0.65] | -0.42 [-0.45, -0.32] | <0.001 |
| cluster (%) |  |  |  |  | <0.001 |
| 1 | 41 (100.0) | 0 ( 0.0) | 0 ( 0.0) | 0 ( 0.0) |  |
| 2 | 0 ( 0.0) | 52 (100.0) | 0 ( 0.0) | 0 ( 0.0) |  |
| 3 | 0 ( 0.0) | 0 ( 0.0) | 18 (100.0) | 0 ( 0.0) |  |
| 4 | 0 ( 0.0) | 0 ( 0.0) | 0 ( 0.0) | 11 (100.0) |  |

**S3: Statistical code with output- R**

**library**(readr)
**library**(ggpubr)

## Loading required package: ggplot2

*#mobility data*
gmr <- **read_csv**("Downloads/Global_Mobility_Report(1).csv", col_types = **cols**(date = **col_date**(format = "%Y-%m-%d")))

**library**(data.table)
**library**(ggplot2)

### Loading country data

countries <- **fread**("http://download.geonames.org/export/dump/countryInfo.txt", skip = "ISO3", na.strings = "")
**names**(countries)[**c**(1,5, 9)] <- **c**("geo", "Country.Region", "Continent")
countries**$**lang <- **sapply**(**strsplit**(**sapply**(**strsplit**(countries**$**Languages, ","), `[`, 1), "-"), `[`, 1)
countries**$**translated <- "Coronavirus"

### Obtaining the case numbers

jhu_url <- **paste0**("https://raw.githubusercontent.com/CSSEGISandData/COVID-19/master/csse_covid_19_data/",
 "csse_covid_19_time_series/time_series_covid19_confirmed_global.csv")
CaseData <- **fread**(jhu_url, check.names = TRUE)
CaseData**$**Province.State[ CaseData**$**Province.State**==**"" ] <- CaseData**$**Country.Region[ CaseData**$**Province.State**==**"" ]
CaseData <- **melt**(CaseData, id.vars = 1**:**4, variable.name = "Date", variable.factor = FALSE)
CaseData**$**Date <- **as.Date**( **substring**(CaseData**$**Date, 2), format = "%m.%d.%y" )
CaseData <- CaseData[ , .(CumCaseNumber = **sum**(value)), .(Country.Region, Date)][**order**(Country.Region, Date)]
CaseData <- CaseData[ ,.(date = Date[**-**1], CumCaseNumber = CumCaseNumber[**-**1], IncCaseNumber = **diff**(CumCaseNumber)),
 .(Country.Region)]

CaseData[Country.Region**==**"US"]**$**Country.Region <- "United States"
CaseData <- **merge**(CaseData,countries[,**c**("Country.Region", "geo", "Continent")])
CaseData**$**country_region<-CaseData**$**Country.Region

CaseData**$**IncCaseNumber<-**ifelse**(CaseData**$**IncCaseNumber**<**0, 0, CaseData**$**IncCaseNumber )
*#write.csv(CaseData, "CaseNumbers1407JHUreal.csv")*
**levels**(**factor**(gmr**$**country_region))

## [1] "Afghanistan" "Angola" "Antigua and Barbuda"
## [4] "Argentina" "Aruba" "Australia"
## [7] "Austria" "Bahrain" "Bangladesh"
## [10] "Barbados" "Belarus" "Belgium"
## [13] "Belize" "Benin" "Bolivia"
## [16] "Bosnia and Herzegovina" "Botswana" "Brazil"
## [19] "Bulgaria" "Burkina Faso" "Cambodia"
## [22] "Cameroon" "Canada" "Cape Verde"
## [25] "Chile" "Colombia" "Costa Rica"
## [28] "Côte d'Ivoire" "Croatia" "Czechia"
## [31] "Denmark" "Dominican Republic" "Ecuador"
## [34] "Egypt" "El Salvador" "Estonia"
## [37] "Fiji" "Finland" "France"
## [40] "Gabon" "Georgia" "Germany"
## [43] "Ghana" "Greece" "Guatemala"
## [46] "Guinea-Bissau" "Haiti" "Honduras"
## [49] "Hong Kong" "Hungary" "India"
## [52] "Indonesia" "Iraq" "Ireland"
## [55] "Israel" "Italy" "Jamaica"
## [58] "Japan" "Jordan" "Kazakhstan"
## [61] "Kenya" "Kuwait" "Kyrgyzstan"
## [64] "Laos" "Latvia" "Lebanon"
## [67] "Libya" "Liechtenstein" "Lithuania"
## [70] "Luxembourg" "Malaysia" "Mali"
## [73] "Malta" "Mauritius" "Mexico"
## [76] "Moldova" "Mongolia" "Morocco"
## [79] "Mozambique" "Myanmar (Burma)" "Namibia"
## [82] "Nepal" "Netherlands" "New Zealand"
## [85] "Nicaragua" "Niger" "Nigeria"
## [88] "North Macedonia" "Norway" "Oman"
## [91] "Pakistan" "Panama" "Papua New Guinea"
## [94] "Paraguay" "Peru" "Philippines"
## [97] "Poland" "Portugal" "Puerto Rico"
## [100] "Qatar" "Réunion" "Romania"
## [103] "Russia" "Rwanda" "Saudi Arabia"
## [106] "Senegal" "Serbia" "Singapore"
## [109] "Slovakia" "Slovenia" "South Africa"
## [112] "South Korea" "Spain" "Sri Lanka"
## [115] "Sweden" "Switzerland" "Taiwan"
## [118] "Tajikistan" "Tanzania" "Thailand"
## [121] "The Bahamas" "Togo" "Trinidad and Tobago"
## [124] "Turkey" "Uganda" "Ukraine"
## [127] "United Arab Emirates" "United Kingdom" "United States"
## [130] "Uruguay" "Venezuela" "Vietnam"
## [133] "Yemen" "Zambia" "Zimbabwe"

**levels**(**factor**(CaseData**$**country_region))

## [1] "Afghanistan" "Albania"
## [3] "Algeria" "Andorra"
## [5] "Angola" "Antigua and Barbuda"
## [7] "Argentina" "Armenia"
## [9] "Australia" "Austria"
## [11] "Azerbaijan" "Bahamas"
## [13] "Bahrain" "Bangladesh"
## [15] "Barbados" "Belarus"
## [17] "Belgium" "Belize"
## [19] "Benin" "Bhutan"
## [21] "Bolivia" "Bosnia and Herzegovina"
## [23] "Botswana" "Brazil"
## [25] "Brunei" "Bulgaria"
## [27] "Burkina Faso" "Burundi"
## [29] "Cabo Verde" "Cambodia"
## [31] "Cameroon" "Canada"
## [33] "Central African Republic" "Chad"
## [35] "Chile" "China"
## [37] "Colombia" "Comoros"
## [39] "Costa Rica" "Croatia"
## [41] "Cuba" "Cyprus"
## [43] "Czechia" "Denmark"
## [45] "Djibouti" "Dominica"
## [47] "Dominican Republic" "Ecuador"
## [49] "Egypt" "El Salvador"
## [51] "Equatorial Guinea" "Eritrea"
## [53] "Estonia" "Eswatini"
## [55] "Ethiopia" "Fiji"
## [57] "Finland" "France"
## [59] "Gabon" "Gambia"
## [61] "Georgia" "Germany"
## [63] "Ghana" "Greece"
## [65] "Grenada" "Guatemala"
## [67] "Guinea" "Guinea-Bissau"
## [69] "Guyana" "Haiti"
## [71] "Honduras" "Hungary"
## [73] "Iceland" "India"
## [75] "Indonesia" "Iran"
## [77] "Iraq" "Ireland"
## [79] "Israel" "Italy"
## [81] "Jamaica" "Japan"
## [83] "Jordan" "Kazakhstan"
## [85] "Kenya" "Kosovo"
## [87] "Kuwait" "Kyrgyzstan"
## [89] "Laos" "Latvia"
## [91] "Lebanon" "Lesotho"
## [93] "Liberia" "Libya"
## [95] "Liechtenstein" "Lithuania"
## [97] "Luxembourg" "Madagascar"
## [99] "Malawi" "Malaysia"
## [101] "Maldives" "Mali"
## [103] "Malta" "Mauritania"
## [105] "Mauritius" "Mexico"
## [107] "Moldova" "Monaco"
## [109] "Mongolia" "Montenegro"
## [111] "Morocco" "Mozambique"
## [113] "Namibia" "Nepal"
## [115] "Netherlands" "New Zealand"
## [117] "Nicaragua" "Niger"
## [119] "Nigeria" "North Macedonia"
## [121] "Norway" "Oman"
## [123] "Pakistan" "Panama"
## [125] "Papua New Guinea" "Paraguay"
## [127] "Peru" "Philippines"
## [129] "Poland" "Portugal"
## [131] "Qatar" "Romania"
## [133] "Russia" "Rwanda"
## [135] "Saint Kitts and Nevis" "Saint Lucia"
## [137] "Saint Vincent and the Grenadines" "San Marino"
## [139] "Sao Tome and Principe" "Saudi Arabia"
## [141] "Senegal" "Serbia"
## [143] "Seychelles" "Sierra Leone"
## [145] "Singapore" "Slovakia"
## [147] "Slovenia" "Somalia"
## [149] "South Africa" "South Sudan"
## [151] "Spain" "Sri Lanka"
## [153] "Sudan" "Suriname"
## [155] "Sweden" "Switzerland"
## [157] "Syria" "Tajikistan"
## [159] "Tanzania" "Thailand"
## [161] "Togo" "Trinidad and Tobago"
## [163] "Tunisia" "Turkey"
## [165] "Uganda" "Ukraine"
## [167] "United Arab Emirates" "United Kingdom"
## [169] "United States" "Uruguay"
## [171] "Uzbekistan" "Venezuela"
## [173] "Vietnam" "Western Sahara"
## [175] "Yemen" "Zambia"
## [177] "Zimbabwe"

countryanalysis<- **function**(x) {
 xgmr<-**subset**(gmr, country_region**==**x **&** **is.na**(sub_region_1)**==**TRUE)
 xgmr<-xgmr[**c**(2,7**:**13)]
 xmerged<-**merge**(xgmr, CaseData, by=**c**("country_region", "date"))
 crosscorr<-**function**(b) {ccfkendall <-**sapply**( **-**28**:**28, **function**(l) **cor.test**(b, Hmisc**::Lag**(xmerged**$**IncCaseNumber,l),method = "kendall", use = "complete.obs")**$**estimate )
 }
 cc<-**as.data.frame**(**lapply**(xmerged[3**:**8], crosscorr))
 ccabs<-**abs**(cc)
 a<-**as.data.frame**(**lapply**(ccabs[1**:**6], which.max))
 b<-**as.data.frame**(**lapply**(cc[1**:**6], max ))
 c<-**as.data.frame**(**lapply**(cc[1**:**6], min))
 e<-**ifelse**(**abs**(b)**<abs**(c), c, b)
 **names**(e) <-**c**("V1", "V2", "V3", "V4", "V5", "V6")
 a**$**country_region<-x
 a<-**data.frame**(**cbind**(a,e))
 }
results<-NULL
d<-NULL

**for**( country_region **in** **unique**(gmr**$**country_region) ) {
 skip_to_next <- FALSE
 **tryCatch**(d<-**countryanalysis**(country_region), **tryCatch**(results<-**rbind**(results, **data.frame**(d))), error = **function**(e) { skip_to_next <<- TRUE})
 **if**(skip_to_next) { **next** }

 }

results<-**unique**(results)
results**$**retail_and_recreation_percent_change_from_baseline<-results**$**retail_and_recreation_percent_change_from_baseline**-**29
results**$**grocery_and_pharmacy_percent_change_from_baseline<- results**$**grocery_and_pharmacy_percent_change_from_baseline**-**29
results**$**parks_percent_change_from_baseline<- results**$**parks_percent_change_from_baseline**-**29
results**$**transit_stations_percent_change_from_baseline<-results**$**transit_stations_percent_change_from_baseline**-**29
results**$**workplaces_percent_change_from_baseline<-results**$**workplaces_percent_change_from_baseline**-**29
results**$**residential_percent_change_from_baseline<-results**$**workplaces_percent_change_from_baseline**-**29


results<-**merge**(**unique**(CaseData[,6**:**7]), results, by="country_region")

**lapply**(results[,3**:**14], shapiro.test)

## $retail_and_recreation_percent_change_from_baseline
##
## Shapiro-Wilk normality test
##
## data: X[[i]]
## W = 0.91749, p-value = 1.453e-06
##
##
## $grocery_and_pharmacy_percent_change_from_baseline
##
## Shapiro-Wilk normality test
##
## data: X[[i]]
## W = 0.9132, p-value = 8.296e-07
##
##
## $parks_percent_change_from_baseline
##
## Shapiro-Wilk normality test
##
## data: X[[i]]
## W = 0.88994, p-value = 5.096e-08
##
##
## $transit_stations_percent_change_from_baseline
##
## Shapiro-Wilk normality test
##
## data: X[[i]]
## W = 0.92098, p-value = 2.319e-06
##
##
## $workplaces_percent_change_from_baseline
##
## Shapiro-Wilk normality test
##
## data: X[[i]]
## W = 0.92489, p-value = 3.973e-06
##
##
## $residential_percent_change_from_baseline
##
## Shapiro-Wilk normality test
##
## data: X[[i]]
## W = 0.92489, p-value = 3.973e-06
##
##
## $V1
##
## Shapiro-Wilk normality test
##
## data: X[[i]]
## W = 0.82322, p-value = 8.511e-11
##
##
## $V2
##
## Shapiro-Wilk normality test
##
## data: X[[i]]
## W = 0.82111, p-value = 7.151e-11
##
##
## $V3
##
## Shapiro-Wilk normality test
##
## data: X[[i]]
## W = 0.84402, p-value = 5.132e-10
##
##
## $V4
##
## Shapiro-Wilk normality test
##
## data: X[[i]]
## W = 0.8227, p-value = 8.157e-11
##
##
## $V5
##
## Shapiro-Wilk normality test
##
## data: X[[i]]
## W = 0.8301, p-value = 1.517e-10
##
##
## $V6
##
## Shapiro-Wilk normality test
##
## data: X[[i]]
## W = 0.79737, p-value = 1.101e-11

**library**(dunn.test)

percontinetanalysislag<-**function**(x) {
 **print**(x)
p<-**ggplot**(results, **aes**(Continent, x)) **+** **geom_boxplot**()**+** **geom_jitter**()**+** **xlab**("Continent") **+** **ylab**("Lag in days") **+** **labs**(x)
**print**(p)
**dunn.test**(x, results**$**Continent, method="holm")}

percontinetanalysistau<-**function**(x) {
 **print**(x)
p<-**ggplot**(results, **aes**(Continent, x)) **+** **geom_boxplot**()**+** **geom_jitter**()**+** **xlab**("Continent") **+** **ylab**("Kendall Tau") **+** **ggtitle**(x)
**print**(p)
**dunn.test**(x, results**$**Continent, method="holm")}


**lapply**(results[,3**:**8], percontinetanalysislag)

## [1] 1 14 11 28 15 3 -28 -28 15 -26 -6 13 -26 -27 -4 4 28 27
## [19] 4 -24 -1 -11 -6 28 3 2 1 2 28 28 -21 -28 1 6 -4 2
## [37] -24 -7 2 -26 9 28 -13 28 -5 -28 -25 28 7 3 7 0 15 4
## [55] 28 -28 -15 28 10 3 4 26 0 4 6 -27 3 0 -26 27 12 -7
## [73] -28 20 28 0 10 -2 -4 -28 -7 3 -28 28 -6 7 27 -28 -25 27
## [91] -5 -28 -4 -28 -1 -23 -28 -7 1 3 4 -28 2 -22 27 9 -10 16
## [109] 12 -27 20 -5 -28 27 -28 2 -12 1 27 12 -16 -26


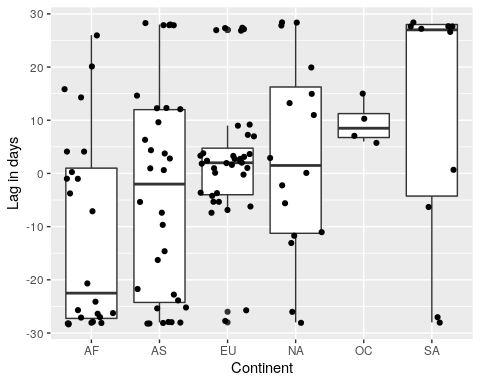


## Kruskal-Wallis rank sum test
##
## data: x and group
## Kruskal-Wallis chi-squared = 14.4404, df = 5, p-value = 0.01
##
##
## Comparison of x by group
## (Holm)
## Col Mean-|
## Row Mean | AF AS EU NA OC
## ---------+-------------------------------------------------------
## AS | -1.644285
## | 0.4505
## |
## EU | -2.672084 -1.070761
## | 0.0528 0.8528
## |
## NA | -2.279588 -0.952773 -0.105097
## | 0.1358 0.6814 0.4581
## |
## OC | -2.455773 -1.663607 -1.180376 -1.056381
## | 0.0914 0.4810 0.9514 0.7270
## |
## SA | -2.917747 -1.805742 -1.102344 -0.899168 0.385506
## | 0.0264 0.3903 0.9461 0.5528 0.6999
##
## alpha = 0.05
## Reject Ho if p <= alpha/2
## [1] 4 15 10 28 15 3 -28 -27 16 -26 1 -24 -22 -27 -3 5 28 27
## [19] 4 -24 -1 -4 -6 28 10 4 3 -12 28 28 28 -28 3 7 -4 4
## [37] -28 -6 6 -26 10 -26 -12 28 2 28 -25 28 4 5 3 4 25 4
## [55] -28 -24 1 28 11 3 -22 -26 -3 4 7 -25 3 0 -23 28 17 -7
## [73] -28 -16 28 3 11 -2 -7 -27 -4 -4 -26 28 -6 -11 27 -28 28 27
## [91] -4 -28 -4 -28 -1 -23 -24 -10 -3 3 4 -28 2 -28 28 4 -11 16
## [109] 10 -27 20 -5 -26 28 -26 4 -4 4 27 12 -16 28


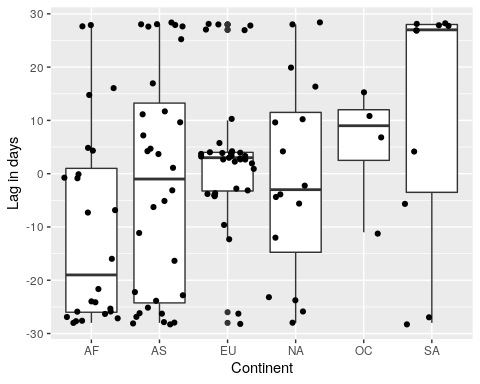


## Kruskal-Wallis rank sum test
##
## data: x and group
## Kruskal-Wallis chi-squared = 10.2439, df = 5, p-value = 0.07
##
##
## Comparison of x by group
## (Holm)
## Col Mean-|
## Row Mean | AF AS EU NA OC
## ---------+-------------------------------------------------------
## AS | -1.675444
## | 0.5631
## |
## EU | -2.453453 -0.798991
## | 0.0990 1.0000
## |
## NA | -1.522557 -0.127313 0.516332
## | 0.6393 0.8987 0.9084
## |
## OC | -1.756373 -0.935508 -0.573020 -0.817768
## | 0.5137 1.0000 1.0000 1.0000
## |
## SA | -2.685858 -1.541602 -1.019354 -1.288763 -0.105425
## | 0.0543 0.6774 1.0000 0.8887 0.4580
##
## alpha = 0.05
## Reject Ho if p <= alpha/2
## [1] -28 9 11 -26 14 -1 -28 -28 12 25 -6 13 23 -27 28 -3 -25 26
## [19] 4 -28 -1 -23 -13 28 3 2 22 -24 28 28 -28 -28 -24 -28 27 -2
## [37] -10 -7 -26 -23 -6 -26 -13 -18 -17 -28 -26 28 8 -2 2 4 -16 3
## [55] 28 -28 1 28 11 -27 -28 28 -28 -1 6 -25 0 3 -26 27 10 -8
## [73] 0 21 28 -27 6 -2 -2 -28 28 -24 -22 28 -14 12 27 27 -26 28
## [91] -5 -28 -15 28 1 -24 -28 -14 2 -21 1 -27 2 -22 -6 23 -8 23
## [109] 13 28 15 -5 -24 28 -28 -11 -12 1 27 -25 -19 27


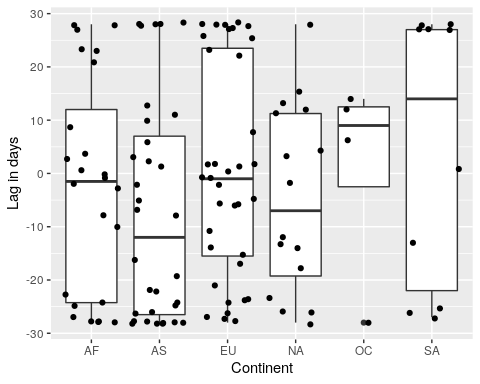


## Kruskal-Wallis rank sum test
##
## data: x and group
## Kruskal-Wallis chi-squared = 4.0549, df = 5, p-value = 0.54
##
##
## Comparison of x by group
## (Holm)
## Col Mean-|
## Row Mean | AF AS EU NA OC
## ---------+-------------------------------------------------------
## AS | 0.736809
## | 1.0000
## |
## EU | -0.772181 -1.656466
## | 1.0000 0.7322
## |
## NA | -0.036585 -0.688369 0.637949
## | 0.4854 1.0000 1.0000
## |
## OC | -0.441648 -0.824917 -0.066464 -0.405550
## | 1.0000 1.0000 0.9470 1.0000
## |
## SA | -0.972515 -1.559556 -0.454747 -0.878745 -0.215555
## | 1.0000 0.8321 1.0000 1.0000 1.0000
##
## alpha = 0.05
## Reject Ho if p <= alpha/2
## [1] 1 2 10 28 16 3 -28 -28 15 -24 -2 13 -25 -27 -3 5 28 28
## [19] 4 -28 -1 -10 1 28 10 3 -3 2 28 28 -21 -28 -1 6 -1 3
## [37] -19 4 2 -26 10 -28 -28 -18 -2 28 -18 28 -2 -2 3 -3 15 4
## [55] 28 -27 0 28 10 5 7 28 -3 5 7 -17 3 4 -28 28 17 -7
## [73] 0 17 28 -3 10 21 1 -28 -6 6 -28 28 1 5 27 27 28 28
## [91] 1 -28 -10 -28 2 -23 -24 -10 -5 1 6 -28 3 -22 -22 6 -10 16
## [109] 13 2 -27 -5 -28 28 -28 0 -8 1 28 14 -16 -26


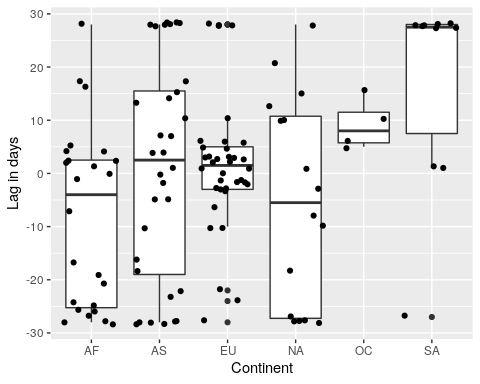


## Kruskal-Wallis rank sum test
##
## data: x and group
## Kruskal-Wallis chi-squared = 14.2374, df = 5, p-value = 0.01
##
##
## Comparison of x by group
## (Holm)
## Col Mean-|
## Row Mean | AF AS EU NA OC
## ---------+-------------------------------------------------------
## AS | -1.641874
## | 0.4528
## |
## EU | -1.628992 0.057950
## | 0.4133 0.4769
## |
## NA | -0.396274 1.030287 1.003054
## | 1.0000 0.7572 0.6317
## |
## OC | -2.122076 -1.325015 -1.359984 -1.821331
## | 0.1861 0.5555 0.6084 0.3428
## |
## SA | -3.269011 -2.172476 -2.241183 -2.735000 -0.142592
## | 0.0081* 0.1789 0.1626 0.0437 0.8866
##
## alpha = 0.05
## Reject Ho if p <= alpha/2
## [1] 1 11 21 28 17 5 -28 -22 12 -22 -3 -26 -25 28 -1 8 28 28
## [19] 1 -27 7 -7 -3 28 14 2 0 6 28 28 -20 -23 6 11 -2 0
## [37] -28 4 6 -23 10 -23 -9 28 -2 28 -22 28 -2 -2 5 -2 14 3
## [55] -23 -24 -4 28 -26 5 5 -9 -2 6 4 -22 3 4 -28 28 26 -10
## [73] -24 -13 28 -3 7 1 -2 -28 4 7 -24 -22 -4 5 28 27 27 28
## [91] -1 -28 -9 -25 -1 -25 -28 -9 -2 6 6 -28 6 -27 -14 6 -10 14
## [109] 14 26 16 2 -28 28 -28 0 -8 6 28 15 -16 -21


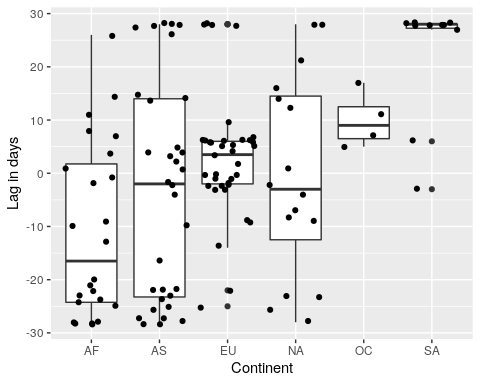


## Kruskal-Wallis rank sum test
##
## data: x and group
## Kruskal-Wallis chi-squared = 26.2134, df = 5, p-value = 0
##
##
## Comparison of x by group
## (Holm)
## Col Mean-|
## Row Mean | AF AS EU NA OC
## ---------+-------------------------------------------------------
## AS | -1.399224
## | 0.4044
## |
## EU | -2.825737 -1.509792
## | 0.0283 0.4588
## |
## NA | -1.590471 -0.442504 0.769895
## | 0.4469 0.3291 0.6620
## |
## OC | -2.457387 -1.790030 -1.105204 -1.455802
## | 0.0700 0.3305 0.5381 0.4363
## |
## SA | -4.634629 -3.772111 -2.796853 -3.053954 -0.705315
## | 0.0000* 0.0011* 0.0284 0.0147* 0.4806
##
## alpha = 0.05
## Reject Ho if p <= alpha/2
## [1] -28 -18 -8 -1 -12 -24 -57 -51 -17 -51 -32 -55 -54 -1 -30 -21 -1 -1
## [19] -28 -56 -22 -36 -32 -1 -15 -27 -29 -23 -1 -1 -49 -52 -23 -18 -31 -29
## [37] -57 -25 -23 -52 -19 -52 -38 -1 -31 -1 -51 -1 -31 -31 -24 -31 -15 -26
## [55] -52 -53 -33 -1 -55 -24 -24 -38 -31 -23 -25 -51 -26 -25 -57 -1 -3 -39
## [73] -53 -42 -1 -32 -22 -28 -31 -57 -25 -22 -53 -51 -33 -24 -1 -2 -2 -1
## [91] -30 -57 -38 -54 -30 -54 -57 -38 -31 -23 -23 -57 -23 -56 -43 -23 -39 -15
## [109] -15 -3 -13 -27 -57 -1 -57 -29 -37 -23 -1 -14 -45 -50


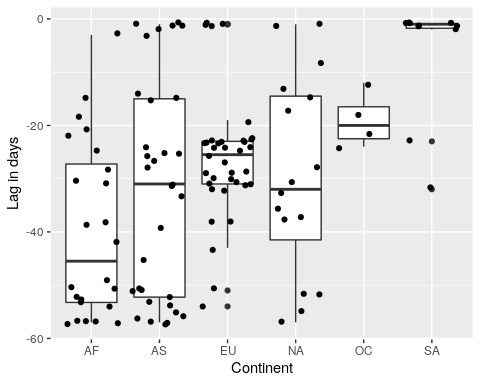


## Kruskal-Wallis rank sum test
##
## data: x and group
## Kruskal-Wallis chi-squared = 26.2134, df = 5, p-value = 0
##
##
## Comparison of x by group
## (Holm)
## Col Mean-|
## Row Mean | AF AS EU NA OC
## ---------+-------------------------------------------------------
## AS | -1.399224
## | 0.4044
## |
## EU | -2.825737 -1.509792
## | 0.0283 0.4588
## |
## NA | -1.590471 -0.442504 0.769895
## | 0.4469 0.3291 0.6620
## |
## OC | -2.457387 -1.790030 -1.105204 -1.455802
## | 0.0700 0.3305 0.5381 0.4363
## |
## SA | -4.634629 -3.772111 -2.796853 -3.053954 -0.705315
## | 0.0000* 0.0011* 0.0284 0.0147* 0.4806
##
## alpha = 0.05
## Reject Ho if p <= alpha/2

## $retail_and_recreation_percent_change_from_baseline
## $retail_and_recreation_percent_change_from_baseline$chi2
## [1] 14.44038
##
## $retail_and_recreation_percent_change_from_baseline$Z
## [1] -1.6442859 -2.6720848 -1.0707613 -2.2795885 -0.9527733 -0.1050974
## [7] -2.4557730 -1.6636074 -1.1803761 -1.0563811 -2.9177477 -1.8057425
## [13] -1.1023442 -0.8991683 0.3855064
##
## $retail_and_recreation_percent_change_from_baseline$P
## [1] 0.050058577 0.003769080 0.142138393 0.011316054 0.170352467 0.458149268
## [7] 0.007029096 0.048095455 0.118925330 0.145397064 0.001762848 0.035479286
## [13] 0.135156036 0.184281524 0.349931116
##
## $retail_and_recreation_percent_change_from_baseline$P.adjusted
## [1] 0.45052719 0.05276712 0.85283036 0.13579265 0.68140987 0.45814927
## [7] 0.09137825 0.48095455 0.95140264 0.72698532 0.02644272 0.39027214
## [13] 0.94609225 0.55284457 0.69986223
##
## $retail_and_recreation_percent_change_from_baseline$comparisons
## [1] "AF - AS" "AF - EU" "AS - EU" "AF - NA" "AS - NA" "EU - NA" "AF - OC"
## [8] "AS - OC" "EU - OC" "NA - OC" "AF - SA" "AS - SA" "EU - SA" "NA - SA"
## [15] "OC - SA"
##
##
## $grocery_and_pharmacy_percent_change_from_baseline
## $grocery_and_pharmacy_percent_change_from_baseline$chi2
## [1] 10.2439
##
## $grocery_and_pharmacy_percent_change_from_baseline$Z
## [1] -1.6754445 -2.4534534 -0.7989914 -1.5225572 -0.1273131 0.5163322
## [7] -1.7563733 -0.9355084 -0.5730203 -0.8177690 -2.6858584 -1.5416028
## [13] -1.0193545 -1.2887635 -0.1054252
##
## $grocery_and_pharmacy_percent_change_from_baseline$P
## [1] 0.046923528 0.007074594 0.212147693 0.063934763 0.449346310 0.302811207
## [7] 0.039512348 0.174763177 0.283315465 0.206744562 0.003617187 0.061585076
## [13] 0.154017349 0.098740168 0.458019197
##
## $grocery_and_pharmacy_percent_change_from_baseline$P.adjusted
## [1] 0.56308234 0.09904431 1.00000000 0.63934763 0.89869262 0.90843362
## [7] 0.51366053 1.00000000 1.00000000 1.00000000 0.05425780 0.67743584
## [13] 1.00000000 0.88866151 0.45801920
##
## $grocery_and_pharmacy_percent_change_from_baseline$comparisons
## [1] "AF - AS" "AF - EU" "AS - EU" "AF - NA" "AS - NA" "EU - NA" "AF - OC"
## [8] "AS - OC" "EU - OC" "NA - OC" "AF - SA" "AS - SA" "EU - SA" "NA - SA"
## [15] "OC - SA"
##
##
## $parks_percent_change_from_baseline
## $parks_percent_change_from_baseline$chi2
## [1] 4.054902
##
## $parks_percent_change_from_baseline$Z
## [1] 0.73680959 -0.77218134 -1.65646634 -0.03658511 -0.68836908 0.63794913
## [7] -0.44164848 -0.82491788 -0.06646435 -0.40555054 -0.97251571 -1.55955629
## [13] -0.45474706 -0.87874596 -0.21555520
##
## $parks_percent_change_from_baseline$P
## [1] 0.23061907 0.22000351 0.04881371 0.48540791 0.24561020 0.26175340
## [7] 0.32937180 0.20470911 0.47350407 0.34253644 0.16539703 0.05943239
## [13] 0.32464561 0.18976952 0.41466724
##
## $parks_percent_change_from_baseline$P.adjusted
## [1] 1.0000000 1.0000000 0.7322056 0.4854079 1.0000000 1.0000000 1.0000000
## [8] 1.0000000 0.9470081 1.0000000 1.0000000 0.8320534 1.0000000 1.0000000
## [15] 1.0000000
##
## $parks_percent_change_from_baseline$comparisons
## [1] "AF - AS" "AF - EU" "AS - EU" "AF - NA" "AS - NA" "EU - NA" "AF - OC"
## [8] "AS - OC" "EU - OC" "NA - OC" "AF - SA" "AS - SA" "EU - SA" "NA - SA"
## [15] "OC - SA"
##
##
## $transit_stations_percent_change_from_baseline
## $transit_stations_percent_change_from_baseline$chi2
## [1] 14.23744
##
## $transit_stations_percent_change_from_baseline$Z
## [1] -1.64187455 -1.62899279 0.05795077 -0.39627456 1.03028718 1.00305403
## [7] -2.12207636 -1.32501519 -1.35998481 -1.82133131 -3.26901120 -2.17247631
## [13] -2.24118343 -2.73500075 -0.14259256
##
## $transit_stations_percent_change_from_baseline$P
## [1] 0.0503080030 0.0516572727 0.4768939193 0.3459512457 0.1514376077
## [6] 0.1579173971 0.0169156630 0.0925830562 0.0869173661 0.0342782547
## [11] 0.0005396201 0.0149098774 0.0125070979 0.0031190067 0.4433059873
##
## $transit_stations_percent_change_from_baseline$P.adjusted
## [1] 0.452772027 0.413258182 0.476893919 1.000000000 0.757188038 0.631669588
## [7] 0.186072293 0.555498337 0.608421562 0.342782547 0.008094302 0.178918529
## [13] 0.162592273 0.043666093 0.886611975
##
## $transit_stations_percent_change_from_baseline$comparisons
## [1] "AF - AS" "AF - EU" "AS - EU" "AF - NA" "AS - NA" "EU - NA" "AF - OC"
## [8] "AS - OC" "EU - OC" "NA - OC" "AF - SA" "AS - SA" "EU - SA" "NA - SA"
## [15] "OC - SA"
##
##
## $workplaces_percent_change_from_baseline
## $workplaces_percent_change_from_baseline$chi2
## [1] 26.21336
##
## $workplaces_percent_change_from_baseline$Z
## [1] -1.3992243 -2.8257379 -1.5097923 -1.5904718 -0.4425046 0.7698954
## [7] -2.4573876 -1.7900309 -1.1052040 -1.4558027 -4.6346294 -3.7721120
## [13] -2.7968537 -3.0539549 -0.7053153
##
## $workplaces_percent_change_from_baseline$P
## [1] 8.087287e-02 2.358592e-03 6.554822e-02 5.586425e-02 3.290620e-01
## [6] 2.206810e-01 6.997579e-03 3.672447e-02 1.345356e-01 7.272359e-02
## [11] 1.787887e-06 8.093579e-05 2.580145e-03 1.129230e-03 2.403070e-01
##
## $workplaces_percent_change_from_baseline$P.adjusted
## [1] 4.043644e-01 2.830311e-02 4.588375e-01 4.469140e-01 3.290620e-01
## [6] 6.620429e-01 6.997579e-02 3.305202e-01 5.381424e-01 4.363415e-01
## [11] 2.681831e-05 1.133101e-03 2.838159e-02 1.467999e-02 4.806141e-01
##
## $workplaces_percent_change_from_baseline$comparisons
## [1] "AF - AS" "AF - EU" "AS - EU" "AF - NA" "AS - NA" "EU - NA" "AF - OC"
## [8] "AS - OC" "EU - OC" "NA - OC" "AF - SA" "AS - SA" "EU - SA" "NA - SA"
## [15] "OC - SA"
##
##
## $residential_percent_change_from_baseline
## $residential_percent_change_from_baseline$chi2
## [1] 26.21336
##
## $residential_percent_change_from_baseline$Z
## [1] -1.3992243 -2.8257379 -1.5097923 -1.5904718 -0.4425046 0.7698954
## [7] -2.4573876 -1.7900309 -1.1052040 -1.4558027 -4.6346294 -3.7721120
## [13] -2.7968537 -3.0539549 -0.7053153
##
## $residential_percent_change_from_baseline$P
## [1] 8.087287e-02 2.358592e-03 6.554822e-02 5.586425e-02 3.290620e-01
## [6] 2.206810e-01 6.997579e-03 3.672447e-02 1.345356e-01 7.272359e-02
## [11] 1.787887e-06 8.093579e-05 2.580145e-03 1.129230e-03 2.403070e-01
##
## $residential_percent_change_from_baseline$P.adjusted
## [1] 4.043644e-01 2.830311e-02 4.588375e-01 4.469140e-01 3.290620e-01
## [6] 6.620429e-01 6.997579e-02 3.305202e-01 5.381424e-01 4.363415e-01
## [11] 2.681831e-05 1.133101e-03 2.838159e-02 1.467999e-02 4.806141e-01
##
## $residential_percent_change_from_baseline$comparisons
## [1] "AF - AS" "AF - EU" "AS - EU" "AF - NA" "AS - NA" "EU - NA" "AF - OC"
## [8] "AS - OC" "EU - OC" "NA - OC" "AF - SA" "AS - SA" "EU - SA" "NA - SA"
## [15] "OC - SA"

**lapply**(results[,9**:**14], percontinetanalysistau)

## [1] -0.5534977 -0.1582540 -0.2499850 0.3433071 -0.6839898 -0.7298250
## [7] -0.4306285 -0.4168797 -0.4674413 -0.7168852 -0.7499364 -0.3761730
## [13] -0.1706894 -0.3732628 -0.4679852 -0.1464787 0.3857661 0.5474611
## [19] -0.5515555 0.5036446 -0.2180149 -0.7223672 -0.4842100 0.5123257
## [25] -0.3814631 -0.6866255 -0.5964913 -0.4793979 0.4853103 0.3246045
## [31] -0.4173397 -0.5121667 -0.6015127 -0.3708448 -0.6507424 -0.6010145
## [37] -0.3439793 -0.5556870 -0.7306371 -0.2222320 -0.5480343 0.3201554
## [43] -0.3240138 0.3808841 -0.6704902 -0.5826455 -0.4918616 0.3616783
## [49] -0.7783861 -0.5710822 -0.7978336 -0.4036024 -0.6775705 -0.3433811
## [55] 0.3579900 -0.4029831 -0.7252601 0.3866533 -0.4122310 -0.6427474
## [61] -0.2981789 0.2085345 -0.6957270 -0.7393324 -0.5991855 -0.4859687
## [67] -0.5365017 -0.4838013 -0.6379013 0.5947028 0.2845355 -0.5527216
## [73] -0.3200635 -0.2428333 0.3398790 -0.7259035 -0.6530664 -0.2373977
## [79] -0.5939036 -0.4730018 -0.3079249 -0.5758381 -0.4068586 0.4213663
## [85] -0.3514292 -0.2075643 0.3737949 -0.4485486 -0.3441840 0.5074654
## [91] -0.5968379 -0.5845451 -0.5924457 -0.6618138 -0.2841941 -0.5554798
## [97] -0.4770963 -0.6408547 -0.7538761 -0.6176974 -0.6249448 -0.2944822
## [103] -0.7418862 -0.4021148 0.4921492 -0.7605880 -0.7207047 -0.3899516
## [109] -0.6830308 -0.3484619 -0.5556397 -0.5928170 -0.3476033 0.5523905
## [115] -0.6414562 -0.7585932 -0.6943479 -0.6770242 0.3680166 -0.5494112
## [121] -0.3755799 -0.2828116


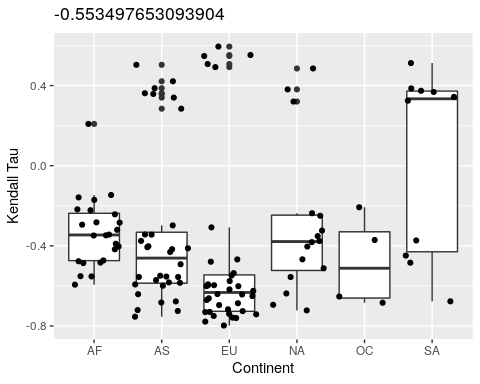


## Kruskal-Wallis rank sum test
##
## data: x and group
## Kruskal-Wallis chi-squared = 25.2021, df = 5, p-value = 0
##
##
## Comparison of x by group
## (Holm)
## Col Mean-|
## Row Mean | AF AS EU NA OC
## ---------+-------------------------------------------------------
## AS | 1.640670
## | 0.5043
## |
## EU | 4.056906 2.576829
## | 0.0004* 0.0598
## |
## NA | 0.666262 -0.744632 -2.842461
## | 0.5052 0.6847 0.0291
## |
## OC | 1.199958 0.386589 -0.798861 0.774603
## | 0.8055 0.3495 1.0000 0.8771
## |
## SA | -0.776362 -2.029466 -3.808259 -1.258326 -1.589336
## | 1.0000 0.2333 0.0010* 0.8331 0.5039
##
## alpha = 0.05
## Reject Ho if p <= alpha/2
## [1] -0.51354801 -0.12938710 -0.27306551 0.36003319 -0.56033235 -0.58347992
## [7] -0.39882599 -0.40948631 -0.46255253 -0.55706838 -0.60497910 0.39737548
## [13] -0.18308884 -0.39696283 -0.37549759 -0.09917067 0.36593233 0.50854969
## [19] -0.51890173 0.51203351 -0.21546209 -0.61399682 -0.54139385 0.43922040
## [25] -0.38532322 -0.55301195 -0.41821734 -0.41923781 0.44326424 0.26721237
## [31] 0.33643340 -0.50096681 -0.49888154 -0.36699504 -0.47116528 -0.52388508
## [37] -0.35068567 -0.51265156 -0.41117673 -0.21280964 -0.34589176 -0.37951396
## [43] -0.27812298 0.27344488 -0.51770147 0.59780899 -0.33697341 0.32737623
## [49] -0.64857755 -0.40839186 -0.66184077 -0.40921771 -0.37177725 -0.27476062
## [55] -0.33003698 -0.38911793 -0.73927898 0.36144433 -0.41991039 -0.50857061
## [61] 0.31359050 0.30324938 -0.43433797 -0.65426120 -0.59264147 -0.45159470
## [67] -0.50457893 -0.43130415 -0.56555128 0.55345038 0.27852564 -0.53071113
## [73] -0.17269028 0.32314266 0.36497813 -0.53534543 -0.57925417 -0.22866182
## [79] -0.45659089 -0.42165697 -0.27038318 -0.31320577 -0.43493240 0.38554247
## [85] -0.35226047 -0.19143881 0.36169863 -0.40966432 0.22776153 0.43421299
## [91] -0.53891293 -0.52963339 -0.57599133 -0.59056810 -0.27730343 -0.54924603
## [97] -0.48553274 -0.51061770 -0.49749072 -0.41519710 -0.54471810 -0.26020249
## [103] -0.68615505 -0.39639005 0.32935841 -0.38734993 -0.69564062 -0.39536215
## [109] -0.59107766 -0.33468418 -0.51362709 -0.28957593 -0.34539200 0.57927103
## [115] -0.57329060 -0.66235837 -0.57689134 -0.57756282 0.31370966 -0.48936936
## [121] -0.21046230 0.29587002


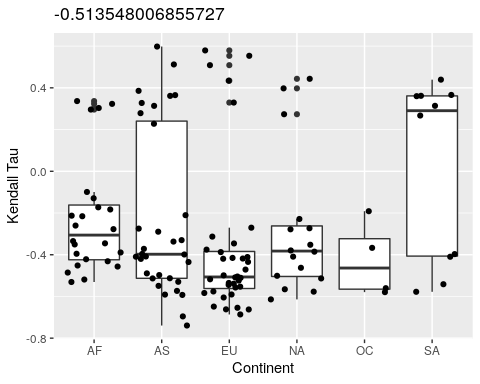


## Kruskal-Wallis rank sum test
##
## data: x and group
## Kruskal-Wallis chi-squared = 13.1473, df = 5, p-value = 0.02
##
##
## Comparison of x by group
## (Holm)
## Col Mean-|
## Row Mean | AF AS EU NA OC
## ---------+-------------------------------------------------------
## AS | 1.091962
## | 0.9620
## |
## EU | 2.976356 2.014663
## | 0.0219* 0.2856
## |
## NA | 0.987528 0.077926 -1.549661
## | 0.9702 0.4689 0.7273
## |
## OC | 1.319954 0.788175 -0.135627 0.705046
## | 0.9343 1.0000 0.8921 0.9616
## |
## SA | -0.472704 -1.305005 -2.691931 -1.232020 -1.505686
## | 0.9546 0.8635 0.0497 0.8718 0.7268
##
## alpha = 0.05
## Reject Ho if p <= alpha/2
## [1] -0.6972263 -0.1406743 -0.2820707 -0.5474599 -0.5749035 -0.5375181
## [7] -0.3931160 -0.6234311 -0.4522494 0.4054679 -0.3836653 -0.3566681
## [13] -0.2067056 -0.3718782 0.3688151 0.1039652 -0.4336895 0.5043227
## [19] -0.4899501 0.4842701 -0.2779962 -0.4746373 -0.5858973 0.4876603
## [25] -0.4227897 -0.5039871 0.2495798 -0.2638605 0.4688289 0.3252128
## [31] -0.3930422 -0.5351902 -0.2399288 0.3504618 0.3268300 -0.4655647
## [37] -0.3344597 -0.5119941 -0.3253124 -0.2394384 -0.3527943 -0.3828613
## [43] -0.3524789 -0.2733995 -0.4825393 -0.6519975 -0.4954826 0.3343076
## [49] -0.2225099 -0.5314196 -0.7393493 -0.4313110 0.2913841 -0.3370043
## [55] 0.4357961 -0.4606250 -0.6931503 0.4095125 -0.3816546 -0.2094598
## [61] 0.2952211 0.2163014 -0.2305994 -0.4814768 -0.6311546 -0.3875723
## [67] -0.4729177 -0.4797767 -0.6855731 0.5944762 0.3030080 -0.5808705
## [73] -0.4261764 -0.2617667 0.3371022 -0.3992977 -0.6225580 -0.1965018
## [79] -0.5713760 -0.5056637 0.3536219 -0.4557112 -0.5191949 0.3717892
## [85] -0.3519320 -0.2161225 0.3223731 0.4726078 -0.4038322 0.4870364
## [91] -0.4975984 -0.6068624 -0.5030600 0.5278162 -0.2783857 -0.6370951
## [97] -0.5926375 -0.5547067 -0.7520335 -0.4266185 -0.4462395 -0.4596031
## [103] -0.7024191 -0.4022653 0.5478339 0.2276984 -0.6935712 -0.3922300
## [109] -0.6517403 0.3261033 -0.5494581 -0.4825813 -0.5079187 0.5795392
## [115] -0.6841033 -0.3826084 -0.3735142 -0.6225834 0.3375566 0.5203411
## [121] -0.5024829 0.3526681


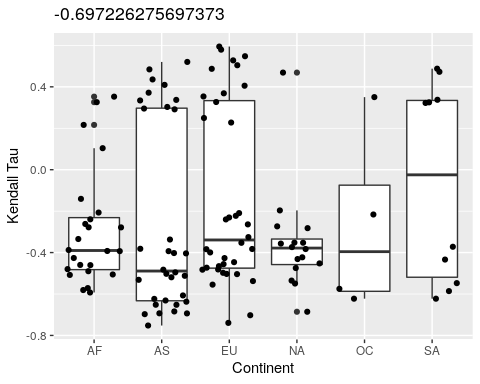


## Kruskal-Wallis rank sum test
##
## data: x and group
## Kruskal-Wallis chi-squared = 5.5825, df = 5, p-value = 0.35
##
##
## Comparison of x by group
## (Holm)
## Col Mean-|
## Row Mean | AF AS EU NA OC
## ---------+-------------------------------------------------------
## AS | 1.036327
## | 1.0000
## |
## EU | -0.961316 -2.194508
## | 1.0000 0.2115
## |
## NA | 0.204442 -0.698454 1.062736
## | 1.0000 1.0000 1.0000
## |
## OC | 0.272717 -0.249950 0.760111 0.145435
## | 1.0000 1.0000 1.0000 0.8844
## |
## SA | -0.660534 -1.458678 0.013184 -0.780425 -0.669194
## | 1.0000 1.0000 0.4947 1.0000 1.0000
##
## alpha = 0.05
## Reject Ho if p <= alpha/2
## [1] -0.5726070 -0.1766209 -0.2377148 0.3923511 -0.7056835 -0.7648966
## [7] -0.4118530 -0.4043994 -0.4777787 -0.7493716 -0.7730150 -0.3907032
## [13] -0.2161860 -0.4006054 -0.4962252 -0.1692275 0.4661006 0.4813273
## [19] -0.6028944 0.4893664 -0.2117865 -0.7299455 -0.6129206 0.5784570
## [25] -0.4327298 -0.7089791 -0.5961135 -0.6363822 0.4634063 0.3488880
## [31] -0.3502570 -0.5903386 -0.6142577 -0.3585332 -0.6622662 -0.5983316
## [37] -0.3223121 -0.5676438 -0.7563131 -0.2306099 -0.5653600 -0.3143399
## [43] -0.2900600 -0.3221777 -0.6614548 0.5657969 -0.5500090 0.3245195
## [49] -0.7411780 -0.6030977 -0.8010124 -0.4282175 -0.6876772 -0.3686877
## [55] 0.3543151 -0.4011943 -0.6819579 0.3256387 -0.4038790 -0.6348178
## [61] -0.3505466 0.2358338 -0.7041428 -0.7674922 -0.6561378 -0.5801924
## [67] -0.5502790 -0.5041723 -0.7276408 0.5086579 0.3007417 -0.5934412
## [73] -0.2986417 -0.2320672 0.3555856 -0.6960974 -0.6688991 -0.2203254
## [79] -0.5821476 -0.4396744 -0.3300947 -0.6845743 -0.4376073 0.4013670
## [85] -0.3445700 -0.1850663 0.3267276 0.4836605 0.2674712 0.4998710
## [91] -0.5931153 -0.6176555 -0.6116445 -0.6455171 -0.2620028 -0.6232359
## [97] -0.5270826 -0.6512402 -0.7481810 -0.6008805 -0.6782901 -0.3452520
## [103] -0.7521519 -0.4010383 -0.3778988 -0.7033298 -0.7297485 -0.3981229
## [109] -0.7173616 -0.3512519 0.5330842 -0.6203814 -0.4045483 0.5437310
## [115] -0.6909615 -0.7465325 -0.6902400 -0.6310369 0.3825599 -0.4806138
## [121] -0.2798201 -0.3112147


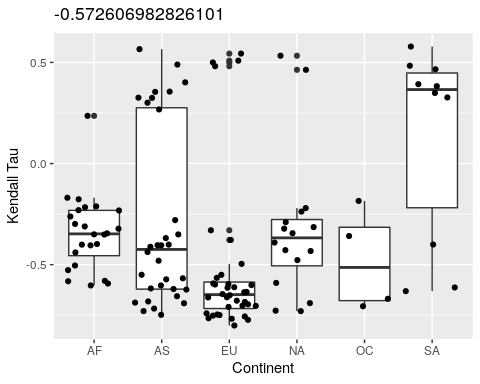


## Kruskal-Wallis rank sum test
##
## data: x and group
## Kruskal-Wallis chi-squared = 27.7418, df = 5, p-value = 0
##
##
## Comparison of x by group
## (Holm)
## Col Mean-|
## Row Mean | AF AS EU NA OC
## ---------+-------------------------------------------------------
## AS | 1.271955
## | 0.8136
## |
## EU | 4.001761 2.926819
## | 0.0004* 0.0223*
## |
## NA | 0.593247 -0.496421 -2.872526
## | 0.8295 0.3098 0.0244*
## |
## OC | 1.162868 0.536559 -0.809294 0.780926
## | 0.7346 0.5916 1.0000 0.8697
## |
## SA | -1.235293 -2.231437 -4.250835 -1.628371 -1.847453
## | 0.7585 0.1411 0.0002* 0.4655 0.3234
##
## alpha = 0.05
## Reject Ho if p <= alpha/2
## [1] -0.5129512 -0.1648298 -0.2531381 0.4585050 -0.6940019 -0.6909161
## [7] -0.4558520 -0.3840352 -0.4677658 -0.6897786 -0.6710472 0.3430625
## [13] -0.1453652 0.4709181 -0.4903037 -0.1553609 0.3602114 0.3784390
## [19] -0.4469237 0.5211389 -0.2065658 -0.6307847 -0.4003338 0.4907732
## [25] -0.4070730 -0.6590935 -0.5708910 -0.5090364 0.4851721 0.3198620
## [31] -0.3296240 -0.4230355 -0.5339414 -0.3421886 -0.4553937 -0.4882581
## [37] -0.4929486 -0.5293585 -0.5902462 -0.1848314 -0.5199858 -0.3273797
## [43] -0.2581092 0.4819861 -0.6160800 0.4438532 -0.5705209 0.3155529
## [49] -0.6399573 -0.5231711 -0.7907835 -0.4206808 -0.7357722 -0.3953319
## [55] -0.3307258 -0.3162344 -0.7141558 0.3144720 0.4346101 -0.5793986
## [61] -0.3029128 -0.1660522 -0.5671096 -0.7254762 -0.6487601 -0.4348773
## [67] -0.5241339 -0.5103348 -0.5352543 0.4421402 0.3265441 -0.5696662
## [73] -0.2770015 0.2226941 0.3221619 -0.5180469 -0.6593436 -0.1928450
## [79] -0.4930406 -0.4545520 -0.3149534 -0.5814699 -0.4561900 -0.3004274
## [85] -0.2479336 -0.1984179 0.3114031 0.5226940 0.2740420 0.3592096
## [91] -0.5531972 -0.6812070 -0.6016859 -0.5417365 -0.2528229 -0.6277069
## [97] -0.4177485 -0.6308793 -0.7160368 -0.5831742 -0.6828104 -0.3611030
## [103] -0.7263155 -0.4338291 -0.4631977 -0.6661455 -0.6733437 -0.3702215
## [109] -0.5697671 0.2192677 -0.4926538 -0.6435807 -0.3370956 0.4362986
## [115] -0.6455565 -0.7146775 -0.6798596 -0.5991029 0.3176600 -0.4806887
## [121] -0.5491790 -0.2997764


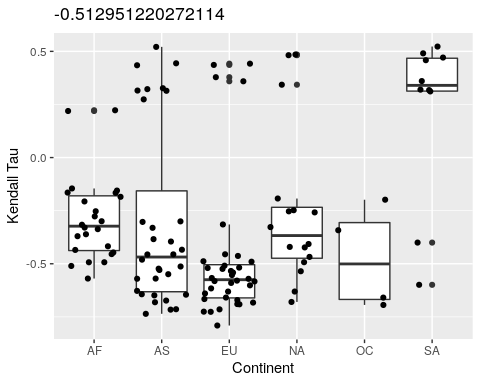


## Kruskal-Wallis rank sum test
##
## data: x and group
## Kruskal-Wallis chi-squared = 31.6534, df = 5, p-value = 0
##
##
## Comparison of x by group
## (Holm)
## Col Mean-|
## Row Mean | AF AS EU NA OC
## ---------+-------------------------------------------------------
## AS | 1.963568
## | 0.2231
## |
## EU | 3.939164 2.090239
## | 0.0006* 0.1830
## |
## NA | 0.339520 -1.373818 -3.090172
## | 0.3671 0.4237 0.0120*
## |
## OC | 1.525038 0.553222 -0.406883 1.277304
## | 0.3817 0.8702 0.6841 0.4030
## |
## SA | -1.684832 -3.213972 -4.678028 -1.844961 -2.464068
## | 0.3221 0.0085* 0.0000* 0.2602 0.0756
##
## alpha = 0.05
## Reject Ho if p <= alpha/2
## [1] 0.6300953 0.1478741 0.3432874 -0.4500194 0.6757498 0.6960393
## [7] 0.6589533 0.4394048 0.4778985 0.5557610 0.7251281 0.3445480
## [13] 0.2014684 -0.3993238 0.4870678 0.1925446 0.3699210 -0.4454225
## [19] 0.5913820 -0.5181839 0.2444784 0.6562264 0.5559480 -0.4287644
## [25] 0.4168785 0.6569495 0.5744941 0.5494818 -0.4293238 0.2827904
## [31] 0.3909724 0.4891530 0.5664777 -0.3624062 0.5686990 0.5327202
## [37] 0.4642563 0.5022371 0.6636567 0.2695202 0.5439880 0.3837516
## [43] 0.3105690 -0.3737575 0.6059915 -0.4177808 0.6149725 -0.3040186
## [49] 0.6386089 0.5292433 0.8120832 0.4672500 0.6803916 0.3665787
## [55] -0.3683447 0.4441851 0.7198547 -0.3742581 0.4127730 0.5684164
## [61] -0.3087898 0.1448971 0.5723127 0.7412086 0.6644456 0.5059272
## [67] 0.5102731 0.4846305 0.6809069 -0.4861235 -0.2962409 0.6455039
## [73] 0.3088654 0.2232864 -0.3288082 0.6401722 0.6402898 0.1824793
## [79] 0.5939883 0.5378781 0.3148797 0.6263767 0.6270833 -0.3564462
## [85] 0.3202322 0.2031230 -0.3167914 -0.4706038 0.3093554 -0.4612694
## [91] 0.5666173 0.6942468 0.5985987 0.5581997 0.2874489 0.6616901
## [97] 0.5419160 0.5875159 0.7105066 0.5654094 0.6572900 0.4018575
## [103] 0.6994592 0.4386276 0.4757399 0.6721069 0.7298431 0.4063304
## [109] 0.6818833 0.3795784 -0.5131745 0.6212946 0.3876472 -0.4854047
## [115] 0.6769021 0.6971996 0.6951800 0.6762063 -0.3022770 0.5393764
## [121] 0.6412054 0.4080639


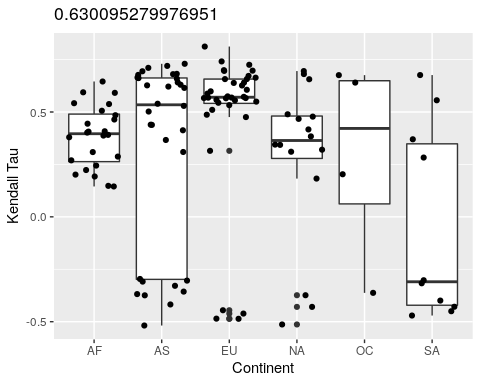


## Kruskal-Wallis rank sum test
##
## data: x and group
## Kruskal-Wallis chi-squared = 19.0864, df = 5, p-value = 0
##
##
## Comparison of x by group
## (Holm)
## Col Mean-|
## Row Mean | AF AS EU NA OC
## ---------+-------------------------------------------------------
## AS | -1.785756
## | 0.4078
## |
## EU | -3.032992 -1.304984
## | 0.0169* 0.7676
## |
## NA | -0.062062 1.509469 2.593444
## | 0.4753 0.6559 0.0570
## |
## OC | -0.626160 0.271612 0.874872 -0.569096
## | 1.0000 0.7859 0.9541 0.8539
## |
## SA | 1.227780 2.606595 3.528738 1.196067 1.352728
## | 0.7684 0.0594 0.0031* 0.6950 0.7926
##
## alpha = 0.05
## Reject Ho if p <= alpha/2

## $V1
## $V1$chi2
## [1] 25.20206
##
## $V1$Z
## [1] 1.6406704 4.0569067 2.5768299 0.6662625 -0.7446329 -2.8424618
## [7] 1.1999584 0.3865894 -0.7988619 0.7746031 -0.7763629 -2.0294663
## [13] -3.8082596 -1.2583266 -1.5893362
##
## $V1$P
## [1] 5.043292e-02 2.486346e-05 4.985550e-03 2.526217e-01 2.282468e-01
## [6] 2.238330e-03 1.150777e-01 3.495301e-01 2.121853e-01 2.192871e-01
## [11] 2.187674e-01 2.120541e-02 6.997415e-05 1.041368e-01 5.599225e-02
##
## $V1$P.adjusted
## [1] 0.5043292280 0.0003729520 0.0598265984 0.5052433054 0.6847404776
## [6] 0.0290982867 0.8055442011 0.3495301194 1.0000000000 0.8771484244
## [11] 1.0000000000 0.2332595002 0.0009796381 0.8330946347 0.5039302804
##
## $V1$comparisons
## [1] "AF - AS" "AF - EU" "AS - EU" "AF - NA" "AS - NA" "EU - NA" "AF - OC"
## [8] "AS - OC" "EU - OC" "NA - OC" "AF - SA" "AS - SA" "EU - SA" "NA - SA"
## [15] "OC - SA"
##
##
## $V2
## $V2$chi2
## [1] 13.14727
##
## $V2$Z
## [1] 1.0919622 2.9763566 2.0146639 0.9875289 0.0779267 -1.5496613
## [7] 1.3199543 0.7881757 -0.1356277 0.7050469 -0.4727048 -1.3050054
## [13] -2.6919319 -1.2320201 -1.5056869
##
## $V2$P
## [1] 0.137424865 0.001458477 0.021969937 0.161691721 0.468943179 0.060611417
## [7] 0.093425143 0.215296966 0.446057816 0.240390518 0.318211898 0.095945509
## [13] 0.003551972 0.108970781 0.066073779
##
## $V2$P.adjusted
## [1] 0.96197405 0.02187716 0.28560918 0.97015033 0.46894318 0.72733701
## [7] 0.93425143 1.00000000 0.89211563 0.96156207 0.95463569 0.86350958
## [13] 0.04972761 0.87176625 0.72681157
##
## $V2$comparisons
## [1] "AF - AS" "AF - EU" "AS - EU" "AF - NA" "AS - NA" "EU - NA" "AF - OC"
## [8] "AS - OC" "EU - OC" "NA - OC" "AF - SA" "AS - SA" "EU - SA" "NA - SA"
## [15] "OC - SA"
##
##
## $V3
## $V3$chi2
## [1] 5.5825
##
## $V3$Z
## [1] 1.03632773 -0.96131699 -2.19450855 0.20444220 -0.69845415 1.06273693
## [7] 0.27271782 -0.24995001 0.76011111 0.14543569 -0.66053453 -1.45867890
## [13] 0.01318497 -0.78042557 -0.66919420
##
## $V3$P
## [1] 0.15002464 0.16819640 0.01409944 0.41900398 0.24244661 0.14395064
## [7] 0.39253507 0.40131300 0.22359409 0.44218344 0.25445543 0.07232675
## [13] 0.49474011 0.21757021 0.25168580
##
## $V3$P.adjusted
## [1] 1.0000000 1.0000000 0.2114915 1.0000000 1.0000000 1.0000000 1.0000000
## [8] 1.0000000 1.0000000 0.8843669 1.0000000 1.0000000 0.4947401 1.0000000
## [15] 1.0000000
##
## $V3$comparisons
## [1] "AF - AS" "AF - EU" "AS - EU" "AF - NA" "AS - NA" "EU - NA" "AF - OC"
## [8] "AS - OC" "EU - OC" "NA - OC" "AF - SA" "AS - SA" "EU - SA" "NA - SA"
## [15] "OC - SA"
##
##
## $V4
## $V4$chi2
## [1] 27.74177
##
## $V4$Z
## [1] 1.2719559 4.0017614 2.9268197 0.5932475 -0.4964220 -2.8725269
## [7] 1.1628688 0.5365594 -0.8092948 0.7809264 -1.2352935 -2.2314372
## [13] -4.2508352 -1.6283711 -1.8474540
##
## $V4$P
## [1] 1.016944e-01 3.143634e-05 1.712237e-03 2.765078e-01 3.097984e-01
## [6] 2.036017e-03 1.224414e-01 2.957860e-01 2.091728e-01 2.174229e-01
## [11] 1.083606e-01 1.282609e-02 1.064874e-05 5.172311e-02 3.234069e-02
##
## $V4$P.adjusted
## [1] 0.8135551056 0.0004401088 0.0222590781 0.8295233259 0.3097983684
## [6] 0.0244322022 0.7346482080 0.5915720188 1.0000000000 0.8696915531
## [11] 0.7585245319 0.1410869980 0.0001597311 0.4655079976 0.3234068627
##
## $V4$comparisons
## [1] "AF - AS" "AF - EU" "AS - EU" "AF - NA" "AS - NA" "EU - NA" "AF - OC"
## [8] "AS - OC" "EU - OC" "NA - OC" "AF - SA" "AS - SA" "EU - SA" "NA - SA"
## [15] "OC - SA"
##
##
## $V5
## $V5$chi2
## [1] 31.65336
##
## $V5$Z
## [1] 1.9635683 3.9391640 2.0902391 0.3395201 -1.3738189 -3.0901723
## [7] 1.5250381 0.5532227 -0.4068830 1.2773047 -1.6848326 -3.2139721
## [13] -4.6780283 -1.8449611 -2.4640686
##
## $V5$P
## [1] 2.479009e-02 4.088301e-05 1.829816e-02 3.671090e-01 8.474896e-02
## [6] 1.000202e-03 6.362480e-02 2.900555e-01 3.420470e-01 1.007473e-01
## [11] 4.601043e-02 6.545619e-04 1.448233e-06 3.252160e-02 6.868490e-03
##
## $V5$P.adjusted
## [1] 2.231108e-01 5.723621e-04 1.829816e-01 3.671090e-01 4.237448e-01
## [6] 1.200242e-02 3.817488e-01 8.701664e-01 6.840939e-01 4.029894e-01
## [11] 3.220730e-01 8.509305e-03 2.172349e-05 2.601728e-01 7.555339e-02
##
## $V5$comparisons
## [1] "AF - AS" "AF - EU" "AS - EU" "AF - NA" "AS - NA" "EU - NA" "AF - OC"
## [8] "AS - OC" "EU - OC" "NA - OC" "AF - SA" "AS - SA" "EU - SA" "NA - SA"
## [15] "OC - SA"
##
##
## $V6
## $V6$chi2
## [1] 19.08643
##
## $V6$Z
## [1] -1.78575632 -3.03299235 -1.30498492 -0.06206281 1.50946909 2.59344411
## [7] -0.62616013 0.27161235 0.87487298 -0.56909617 1.22778030 2.60659578
## [13] 3.52873820 1.19606795 1.35272827
##
## $V6$P
## [1] 0.037069361 0.001210709 0.095948992 0.475256406 0.065589474 0.004750999
## [7] 0.265604959 0.392960051 0.190821512 0.284645438 0.109764726 0.004572362
## [13] 0.000208773 0.115835022 0.088071228
##
## $V6$P.adjusted
## [1] 0.407762974 0.016949922 0.767591934 0.475256406 0.655894743 0.057011989
## [7] 1.000000000 0.785920103 0.954107559 0.853936315 0.768353081 0.059440709
## [13] 0.003131595 0.695010132 0.792641054
##
## $V6$comparisons
## [1] "AF - AS" "AF - EU" "AS - EU" "AF - NA" "AS - NA" "EU - NA" "AF - OC"
## [8] "AS - OC" "EU - OC" "NA - OC" "AF - SA" "AS - SA" "EU - SA" "NA - SA"
## [15] "OC - SA"

**library**(tableone)
vars<-**c**(**colnames**(results[,3**:**14]))
factorvars<-**c**("Continent")
tab1<-**CreateTableOne**(vars=vars, strata="Continent", data=results)
**print**(tab1, nonnormal=vars)

## Stratified by Continent
## AF
## n 24
## retail_and_recreation_percent_change_from_baseline (median [IQR]) -22.50 [-27.25, 1.00]
## grocery_and_pharmacy_percent_change_from_baseline (median [IQR]) -19.00 [-26.00, 1.00]
## parks_percent_change_from_baseline (median [IQR]) -1.50 [-24.25, 12.00]
## transit_stations_percent_change_from_baseline (median [IQR]) -4.00 [-25.25, 2.50]
## workplaces_percent_change_from_baseline (median [IQR]) -16.50 [-24.25, 1.75]
## residential_percent_change_from_baseline (median [IQR]) -45.50 [-53.25, -27.25]
## V1 (median [IQR]) -0.35 [-0.47, -0.24]
## V2 (median [IQR]) -0.31 [-0.42, -0.16]
## V3 (median [IQR]) -0.39 [-0.48, -0.23]
## V4 (median [IQR]) -0.35 [-0.46, -0.23]
## V5 (median [IQR]) -0.32 [-0.44, -0.18]
## V6 (median [IQR]) 0.40 [0.26, 0.49]
## Stratified by Continent
## AS
## n 32
## retail_and_recreation_percent_change_from_baseline (median [IQR]) -2.00 [-24.25, 12.00]
## grocery_and_pharmacy_percent_change_from_baseline (median [IQR]) -1.00 [-24.25, 13.25]
## parks_percent_change_from_baseline (median [IQR]) -12.00 [-26.50, 7.00]
## transit_stations_percent_change_from_baseline (median [IQR]) 2.50 [-19.00, 15.50]
## workplaces_percent_change_from_baseline (median [IQR]) -2.00 [-23.25, 14.00]
## residential_percent_change_from_baseline (median [IQR]) -31.00 [-52.25, -15.00]
## V1 (median [IQR]) -0.46 [-0.59, -0.33]
## V2 (median [IQR]) -0.40 [-0.51, 0.24]
## V3 (median [IQR]) -0.49 [-0.63, 0.30]
## V4 (median [IQR]) -0.42 [-0.62, 0.28]
## V5 (median [IQR]) -0.47 [-0.63, -0.16]
## V6 (median [IQR]) 0.53 [-0.30, 0.66]
## Stratified by Continent
## EU
## n 36
## retail_and_recreation_percent_change_from_baseline (median [IQR]) 2.00 [-4.00, 4.75]
## grocery_and_pharmacy_percent_change_from_baseline (median [IQR]) 3.00 [-3.25, 4.00]
## parks_percent_change_from_baseline (median [IQR]) -1.00 [-15.50, 23.50]
## transit_stations_percent_change_from_baseline (median [IQR]) 1.50 [-3.00, 5.00]
## workplaces_percent_change_from_baseline (median [IQR]) 3.50 [-2.00, 6.00]
## residential_percent_change_from_baseline (median [IQR]) -25.50 [-31.00, -23.00]
## V1 (median [IQR]) -0.63 [-0.73, -0.55]
## V2 (median [IQR]) -0.51 [-0.56, -0.38]
## V3 (median [IQR]) -0.34 [-0.48, 0.33]
## V4 (median [IQR]) -0.65 [-0.72, -0.59]
## V5 (median [IQR]) -0.58 [-0.66, -0.50]
## V6 (median [IQR]) 0.57 [0.54, 0.66]
## Stratified by Continent
## NA
## n 16
## retail_and_recreation_percent_change_from_baseline (median [IQR]) 1.50 [-11.25, 16.25]
## grocery_and_pharmacy_percent_change_from_baseline (median [IQR]) -3.00 [-14.75, 11.50]
## parks_percent_change_from_baseline (median [IQR]) -7.00 [-19.25, 11.25]
## transit_stations_percent_change_from_baseline (median [IQR]) -5.50 [-27.25, 10.75]
## workplaces_percent_change_from_baseline (median [IQR]) -3.00 [-12.50, 14.50]
## residential_percent_change_from_baseline (median [IQR]) -32.00 [-41.50, -14.50]
## V1 (median [IQR]) -0.38 [-0.52, -0.25]
## V2 (median [IQR]) -0.38 [-0.50, -0.26]
## V3 (median [IQR]) -0.38 [-0.46, -0.33]
## V4 (median [IQR]) -0.37 [-0.51, -0.28]
## V5 (median [IQR]) -0.37 [-0.47, -0.23]
## V6 (median [IQR]) 0.36 [0.28, 0.48]
## Stratified by Continent
## OC
## n 4
## retail_and_recreation_percent_change_from_baseline (median [IQR]) 8.50 [6.75, 11.25]
## grocery_and_pharmacy_percent_change_from_baseline (median [IQR]) 9.00 [2.50, 12.00]
## parks_percent_change_from_baseline (median [IQR]) 9.00 [-2.50, 12.50]
## transit_stations_percent_change_from_baseline (median [IQR]) 8.00 [5.75, 11.50]
## workplaces_percent_change_from_baseline (median [IQR]) 9.00 [6.50, 12.50]
## residential_percent_change_from_baseline (median [IQR]) -20.00 [-22.50, -16.50]
## V1 (median [IQR]) -0.51 [-0.66, -0.33]
## V2 (median [IQR]) -0.46 [-0.57, -0.32]
## V3 (median [IQR]) -0.40 [-0.59, -0.07]
## V4 (median [IQR]) -0.51 [-0.68, -0.32]
## V5 (median [IQR]) -0.50 [-0.67, -0.31]
## V6 (median [IQR]) 0.42 [0.06, 0.65]
## Stratified by Continent
## SA
## n 10
## retail_and_recreation_percent_change_from_baseline (median [IQR]) 27.00 [-4.25, 28.00]
## grocery_and_pharmacy_percent_change_from_baseline (median [IQR]) 27.00 [-3.50, 28.00]
## parks_percent_change_from_baseline (median [IQR]) 14.00 [-22.00, 27.00]
## transit_stations_percent_change_from_baseline (median [IQR]) 27.50 [7.50, 28.00]
## workplaces_percent_change_from_baseline (median [IQR]) 28.00 [27.25, 28.00]
## residential_percent_change_from_baseline (median [IQR]) -1.00 [-1.75, -1.00]
## V1 (median [IQR]) 0.33 [-0.43, 0.37]
## V2 (median [IQR]) 0.29 [-0.41, 0.36]
## V3 (median [IQR]) -0.02 [-0.52, 0.33]
## V4 (median [IQR]) 0.37 [-0.22, 0.45]
## V5 (median [IQR]) 0.34 [0.31, 0.47]
## V6 (median [IQR]) -0.31 [-0.42, 0.35]
## Stratified by Continent
## p
## n
## retail_and_recreation_percent_change_from_baseline (median [IQR]) 0.013
## grocery_and_pharmacy_percent_change_from_baseline (median [IQR]) 0.069
## parks_percent_change_from_baseline (median [IQR]) 0.542
## transit_stations_percent_change_from_baseline (median [IQR]) 0.014
## workplaces_percent_change_from_baseline (median [IQR]) <0.001
## residential_percent_change_from_baseline (median [IQR]) <0.001
## V1 (median [IQR]) <0.001
## V2 (median [IQR]) 0.022
## V3 (median [IQR]) 0.349
## V4 (median [IQR]) <0.001
## V5 (median [IQR]) <0.001
## V6 (median [IQR]) 0.002
## Stratified by Continent
## test
## n
## retail_and_recreation_percent_change_from_baseline (median [IQR]) nonnorm
## grocery_and_pharmacy_percent_change_from_baseline (median [IQR]) nonnorm
## parks_percent_change_from_baseline (median [IQR]) nonnorm
## transit_stations_percent_change_from_baseline (median [IQR]) nonnorm
## workplaces_percent_change_from_baseline (median [IQR]) nonnorm
## residential_percent_change_from_baseline (median [IQR]) nonnorm
## V1 (median [IQR]) nonnorm
## V2 (median [IQR]) nonnorm
## V3 (median [IQR]) nonnorm
## V4 (median [IQR]) nonnorm
## V5 (median [IQR]) nonnorm
## V6 (median [IQR]) nonnorm

**write.csv**(**print**(tab1, nonnormal=vars), "correlationtablescovidcontinentrev.csv")

## Stratified by Continent
## AF
## n 24
## retail_and_recreation_percent_change_from_baseline (median [IQR]) -22.50 [-27.25, 1.00]
## grocery_and_pharmacy_percent_change_from_baseline (median [IQR]) -19.00 [-26.00, 1.00]
## parks_percent_change_from_baseline (median [IQR]) -1.50 [-24.25, 12.00]
## transit_stations_percent_change_from_baseline (median [IQR]) -4.00 [-25.25, 2.50]
## workplaces_percent_change_from_baseline (median [IQR]) -16.50 [-24.25, 1.75]
## residential_percent_change_from_baseline (median [IQR]) -45.50 [-53.25, -27.25]
## V1 (median [IQR]) -0.35 [-0.47, -0.24]
## V2 (median [IQR]) -0.31 [-0.42, -0.16]
## V3 (median [IQR]) -0.39 [-0.48, -0.23]
## V4 (median [IQR]) -0.35 [-0.46, -0.23]
## V5 (median [IQR]) -0.32 [-0.44, -0.18]
## V6 (median [IQR]) 0.40 [0.26, 0.49]
## Stratified by Continent
## AS
## n 32
## retail_and_recreation_percent_change_from_baseline (median [IQR]) -2.00 [-24.25, 12.00]
## grocery_and_pharmacy_percent_change_from_baseline (median [IQR]) -1.00 [-24.25, 13.25]
## parks_percent_change_from_baseline (median [IQR]) -12.00 [-26.50, 7.00]
## transit_stations_percent_change_from_baseline (median [IQR]) 2.50 [-19.00, 15.50]
## workplaces_percent_change_from_baseline (median [IQR]) -2.00 [-23.25, 14.00]
## residential_percent_change_from_baseline (median [IQR]) -31.00 [-52.25, -15.00]
## V1 (median [IQR]) -0.46 [-0.59, -0.33]
## V2 (median [IQR]) -0.40 [-0.51, 0.24]
## V3 (median [IQR]) -0.49 [-0.63, 0.30]
## V4 (median [IQR]) -0.42 [-0.62, 0.28]
## V5 (median [IQR]) -0.47 [-0.63, -0.16]
## V6 (median [IQR]) 0.53 [-0.30, 0.66]
## Stratified by Continent
## EU
## n 36
## retail_and_recreation_percent_change_from_baseline (median [IQR]) 2.00 [-4.00, 4.75]
## grocery_and_pharmacy_percent_change_from_baseline (median [IQR]) 3.00 [-3.25, 4.00]
## parks_percent_change_from_baseline (median [IQR]) -1.00 [-15.50, 23.50]
## transit_stations_percent_change_from_baseline (median [IQR]) 1.50 [-3.00, 5.00]
## workplaces_percent_change_from_baseline (median [IQR]) 3.50 [-2.00, 6.00]
## residential_percent_change_from_baseline (median [IQR]) -25.50 [-31.00, -23.00]
## V1 (median [IQR]) -0.63 [-0.73, -0.55]
## V2 (median [IQR]) -0.51 [-0.56, -0.38]
## V3 (median [IQR]) -0.34 [-0.48, 0.33]
## V4 (median [IQR]) -0.65 [-0.72, -0.59]
## V5 (median [IQR]) -0.58 [-0.66, -0.50]
## V6 (median [IQR]) 0.57 [0.54, 0.66]
## Stratified by Continent
## NA
## n 16
## retail_and_recreation_percent_change_from_baseline (median [IQR]) 1.50 [-11.25, 16.25]
## grocery_and_pharmacy_percent_change_from_baseline (median [IQR]) -3.00 [-14.75, 11.50]
## parks_percent_change_from_baseline (median [IQR]) -7.00 [-19.25, 11.25]
## transit_stations_percent_change_from_baseline (median [IQR]) -5.50 [-27.25, 10.75]
## workplaces_percent_change_from_baseline (median [IQR]) -3.00 [-12.50, 14.50]
## residential_percent_change_from_baseline (median [IQR]) -32.00 [-41.50, -14.50]
## V1 (median [IQR]) -0.38 [-0.52, -0.25]
## V2 (median [IQR]) -0.38 [-0.50, -0.26]
## V3 (median [IQR]) -0.38 [-0.46, -0.33]
## V4 (median [IQR]) -0.37 [-0.51, -0.28]
## V5 (median [IQR]) -0.37 [-0.47, -0.23]
## V6 (median [IQR]) 0.36 [0.28, 0.48]
## Stratified by Continent
## OC
## n 4
## retail_and_recreation_percent_change_from_baseline (median [IQR]) 8.50 [6.75, 11.25]
## grocery_and_pharmacy_percent_change_from_baseline (median [IQR]) 9.00 [2.50, 12.00]
## parks_percent_change_from_baseline (median [IQR]) 9.00 [-2.50, 12.50]
## transit_stations_percent_change_from_baseline (median [IQR]) 8.00 [5.75, 11.50]
## workplaces_percent_change_from_baseline (median [IQR]) 9.00 [6.50, 12.50]
## residential_percent_change_from_baseline (median [IQR]) -20.00 [-22.50, -16.50]
## V1 (median [IQR]) -0.51 [-0.66, -0.33]
## V2 (median [IQR]) -0.46 [-0.57, -0.32]
## V3 (median [IQR]) -0.40 [-0.59, -0.07]
## V4 (median [IQR]) -0.51 [-0.68, -0.32]
## V5 (median [IQR]) -0.50 [-0.67, -0.31]
## V6 (median [IQR]) 0.42 [0.06, 0.65]
## Stratified by Continent
## SA
## n 10
## retail_and_recreation_percent_change_from_baseline (median [IQR]) 27.00 [-4.25, 28.00]
## grocery_and_pharmacy_percent_change_from_baseline (median [IQR]) 27.00 [-3.50, 28.00]
## parks_percent_change_from_baseline (median [IQR]) 14.00 [-22.00, 27.00]
## transit_stations_percent_change_from_baseline (median [IQR]) 27.50 [7.50, 28.00]
## workplaces_percent_change_from_baseline (median [IQR]) 28.00 [27.25, 28.00]
## residential_percent_change_from_baseline (median [IQR]) -1.00 [-1.75, -1.00]
## V1 (median [IQR]) 0.33 [-0.43, 0.37]
## V2 (median [IQR]) 0.29 [-0.41, 0.36]
## V3 (median [IQR]) -0.02 [-0.52, 0.33]
## V4 (median [IQR]) 0.37 [-0.22, 0.45]
## V5 (median [IQR]) 0.34 [0.31, 0.47]
## V6 (median [IQR]) -0.31 [-0.42, 0.35]
## Stratified by Continent
## p
## n
## retail_and_recreation_percent_change_from_baseline (median [IQR]) 0.013
## grocery_and_pharmacy_percent_change_from_baseline (median [IQR]) 0.069
## parks_percent_change_from_baseline (median [IQR]) 0.542
## transit_stations_percent_change_from_baseline (median [IQR]) 0.014
## workplaces_percent_change_from_baseline (median [IQR]) <0.001
## residential_percent_change_from_baseline (median [IQR]) <0.001
## V1 (median [IQR]) <0.001
## V2 (median [IQR]) 0.022
## V3 (median [IQR]) 0.349
## V4 (median [IQR]) <0.001
## V5 (median [IQR]) <0.001
## V6 (median [IQR]) 0.002
## Stratified by Continent
## test
## n
## retail_and_recreation_percent_change_from_baseline (median [IQR]) nonnorm
## grocery_and_pharmacy_percent_change_from_baseline (median [IQR]) nonnorm
## parks_percent_change_from_baseline (median [IQR]) nonnorm
## transit_stations_percent_change_from_baseline (median [IQR]) nonnorm
## workplaces_percent_change_from_baseline (median [IQR]) nonnorm
## residential_percent_change_from_baseline (median [IQR]) nonnorm
## V1 (median [IQR]) nonnorm
## V2 (median [IQR]) nonnorm
## V3 (median [IQR]) nonnorm
## V4 (median [IQR]) nonnorm
## V5 (median [IQR]) nonnorm
## V6 (median [IQR]) nonnorm

**library**(dplyr)

##
## Attaching package: 'dplyr'

## The following objects are masked from 'package:data.table':
##
## between, first, last

## The following objects are masked from 'package:stats':
##
## filter, lag

## The following objects are masked from 'package:base':
##
## intersect, setdiff, setequal, union

**require**(maps)

## Loading required package: maps

**require**(viridis)

## Loading required package: viridis

## Loading required package: viridisLite

**theme_set**(**theme_void**())

world_map <- **map_data**("world")


results**$**region<-results**$**country_region
**print**(**levels**(**factor**(world_map**$**region)))

## [1] "Afghanistan"
## [2] "Albania"
## [3] "Algeria"
## [4] "American Samoa"
## [5] "Andorra"
## [6] "Angola"
## [7] "Anguilla"
## [8] "Antarctica"
## [9] "Antigua"
## [10] "Argentina"
## [11] "Armenia"
## [12] "Aruba"
## [13] "Ascension Island"
## [14] "Australia"
## [15] "Austria"
## [16] "Azerbaijan"
## [17] "Azores"
## [18] "Bahamas"
## [19] "Bahrain"
## [20] "Bangladesh"
## [21] "Barbados"
## [22] "Barbuda"
## [23] "Belarus"
## [24] "Belgium"
## [25] "Belize"
## [26] "Benin"
## [27] "Bermuda"
## [28] "Bhutan"
## [29] "Bolivia"
## [30] "Bonaire"
## [31] "Bosnia and Herzegovina"
## [32] "Botswana"
## [33] "Brazil"
## [34] "Brunei"
## [35] "Bulgaria"
## [36] "Burkina Faso"
## [37] "Burundi"
## [38] "Cambodia"
## [39] "Cameroon"
## [40] "Canada"
## [41] "Canary Islands"
## [42] "Cape Verde"
## [43] "Cayman Islands"
## [44] "Central African Republic"
## [45] "Chad"
## [46] "Chagos Archipelago"
## [47] "Chile"
## [48] "China"
## [49] "Christmas Island"
## [50] "Cocos Islands"
## [51] "Colombia"
## [52] "Comoros"
## [53] "Cook Islands"
## [54] "Costa Rica"
## [55] "Croatia"
## [56] "Cuba"
## [57] "Curacao"
## [58] "Cyprus"
## [59] "Czech Republic"
## [60] "Democratic Republic of the Congo"
## [61] "Denmark"
## [62] "Djibouti"
## [63] "Dominica"
## [64] "Dominican Republic"
## [65] "Ecuador"
## [66] "Egypt"
## [67] "El Salvador"
## [68] "Equatorial Guinea"
## [69] "Eritrea"
## [70] "Estonia"
## [71] "Ethiopia"
## [72] "Falkland Islands"
## [73] "Faroe Islands"
## [74] "Fiji"
## [75] "Finland"
## [76] "France"
## [77] "French Guiana"
## [78] "French Polynesia"
## [79] "French Southern and Antarctic Lands"
## [80] "Gabon"
## [81] "Gambia"
## [82] "Georgia"
## [83] "Germany"
## [84] "Ghana"
## [85] "Greece"
## [86] "Greenland"
## [87] "Grenada"
## [88] "Grenadines"
## [89] "Guadeloupe"
## [90] "Guam"
## [91] "Guatemala"
## [92] "Guernsey"
## [93] "Guinea"
## [94] "Guinea-Bissau"
## [95] "Guyana"
## [96] "Haiti"
## [97] "Heard Island"
## [98] "Honduras"
## [99] "Hungary"
## [100] "Iceland"
## [101] "India"
## [102] "Indonesia"
## [103] "Iran"
## [104] "Iraq"
## [105] "Ireland"
## [106] "Isle of Man"
## [107] "Israel"
## [108] "Italy"
## [109] "Ivory Coast"
## [110] "Jamaica"
## [111] "Japan"
## [112] "Jersey"
## [113] "Jordan"
## [114] "Kazakhstan"
## [115] "Kenya"
## [116] "Kiribati"
## [117] "Kosovo"
## [118] "Kuwait"
## [119] "Kyrgyzstan"
## [120] "Laos"
## [121] "Latvia"
## [122] "Lebanon"
## [123] "Lesotho"
## [124] "Liberia"
## [125] "Libya"
## [126] "Liechtenstein"
## [127] "Lithuania"
## [128] "Luxembourg"
## [129] "Macedonia"
## [130] "Madagascar"
## [131] "Madeira Islands"
## [132] "Malawi"
## [133] "Malaysia"
## [134] "Maldives"
## [135] "Mali"
## [136] "Malta"
## [137] "Marshall Islands"
## [138] "Martinique"
## [139] "Mauritania"
## [140] "Mauritius"
## [141] "Mayotte"
## [142] "Mexico"
## [143] "Micronesia"
## [144] "Moldova"
## [145] "Monaco"
## [146] "Mongolia"
## [147] "Montenegro"
## [148] "Montserrat"
## [149] "Morocco"
## [150] "Mozambique"
## [151] "Myanmar"
## [152] "Namibia"
## [153] "Nauru"
## [154] "Nepal"
## [155] "Netherlands"
## [156] "Nevis"
## [157] "New Caledonia"
## [158] "New Zealand"
## [159] "Nicaragua"
## [160] "Niger"
## [161] "Nigeria"
## [162] "Niue"
## [163] "Norfolk Island"
## [164] "North Korea"
## [165] "Northern Mariana Islands"
## [166] "Norway"
## [167] "Oman"
## [168] "Pakistan"
## [169] "Palau"
## [170] "Palestine"
## [171] "Panama"
## [172] "Papua New Guinea"
## [173] "Paraguay"
## [174] "Peru"
## [175] "Philippines"
## [176] "Pitcairn Islands"
## [177] "Poland"
## [178] "Portugal"
## [179] "Puerto Rico"
## [180] "Qatar"
## [181] "Republic of Congo"
## [182] "Reunion"
## [183] "Romania"
## [184] "Russia"
## [185] "Rwanda"
## [186] "Saba"
## [187] "Saint Barthelemy"
## [188] "Saint Helena"
## [189] "Saint Kitts"
## [190] "Saint Lucia"
## [191] "Saint Martin"
## [192] "Saint Pierre and Miquelon"
## [193] "Saint Vincent"
## [194] "Samoa"
## [195] "San Marino"
## [196] "Sao Tome and Principe"
## [197] "Saudi Arabia"
## [198] "Senegal"
## [199] "Serbia"
## [200] "Seychelles"
## [201] "Siachen Glacier"
## [202] "Sierra Leone"
## [203] "Singapore"
## [204] "Sint Eustatius"
## [205] "Sint Maarten"
## [206] "Slovakia"
## [207] "Slovenia"
## [208] "Solomon Islands"
## [209] "Somalia"
## [210] "South Africa"
## [211] "South Georgia"
## [212] "South Korea"
## [213] "South Sandwich Islands"
## [214] "South Sudan"
## [215] "Spain"
## [216] "Sri Lanka"
## [217] "Sudan"
## [218] "Suriname"
## [219] "Swaziland"
## [220] "Sweden"
## [221] "Switzerland"
## [222] "Syria"
## [223] "Taiwan"
## [224] "Tajikistan"
## [225] "Tanzania"
## [226] "Thailand"
## [227] "Timor-Leste"
## [228] "Tobago"
## [229] "Togo"
## [230] "Tonga"
## [231] "Trinidad"
## [232] "Tunisia"
## [233] "Turkey"
## [234] "Turkmenistan"
## [235] "Turks and Caicos Islands"
## [236] "Uganda"
## [237] "UK"
## [238] "Ukraine"
## [239] "United Arab Emirates"
## [240] "Uruguay"
## [241] "USA"
## [242] "Uzbekistan"
## [243] "Vanuatu"
## [244] "Vatican"
## [245] "Venezuela"
## [246] "Vietnam"
## [247] "Virgin Islands"
## [248] "Wallis and Futuna"
## [249] "Western Sahara"
## [250] "Yemen"
## [251] "Zambia"
## [252] "Zimbabwe"

**print**(**levels**(**factor**(results**$**region)))

## [1] "Afghanistan" "Angola" "Antigua and Barbuda"
## [4] "Argentina" "Australia" "Austria"
## [7] "Bahrain" "Bangladesh" "Barbados"
## [10] "Belarus" "Belgium" "Belize"
## [13] "Benin" "Bolivia" "Bosnia and Herzegovina"
## [16] "Botswana" "Brazil" "Bulgaria"
## [19] "Burkina Faso" "Cambodia" "Cameroon"
## [22] "Canada" "Chile" "Colombia"
## [25] "Costa Rica" "Croatia" "Czechia"
## [28] "Denmark" "Dominican Republic" "Ecuador"
## [31] "Egypt" "El Salvador" "Estonia"
## [34] "Fiji" "Finland" "France"
## [37] "Gabon" "Georgia" "Germany"
## [40] "Ghana" "Greece" "Guatemala"
## [43] "Haiti" "Honduras" "Hungary"
## [46] "India" "Indonesia" "Iraq"
## [49] "Ireland" "Israel" "Italy"
## [52] "Jamaica" "Japan" "Jordan"
## [55] "Kazakhstan" "Kenya" "Kuwait"
## [58] "Kyrgyzstan" "Laos" "Latvia"
## [61] "Lebanon" "Libya" "Lithuania"
## [64] "Luxembourg" "Malaysia" "Mali"
## [67] "Malta" "Mauritius" "Mexico"
## [70] "Moldova" "Mongolia" "Morocco"
## [73] "Mozambique" "Namibia" "Nepal"
## [76] "Netherlands" "New Zealand" "Nicaragua"
## [79] "Niger" "Nigeria" "North Macedonia"
## [82] "Norway" "Oman" "Pakistan"
## [85] "Panama" "Papua New Guinea" "Paraguay"
## [88] "Peru" "Philippines" "Poland"
## [91] "Portugal" "Qatar" "Romania"
## [94] "Russia" "Rwanda" "Saudi Arabia"
## [97] "Senegal" "Serbia" "Singapore"
## [100] "Slovakia" "Slovenia" "South Africa"
## [103] "Spain" "Sri Lanka" "Sweden"
## [106] "Switzerland" "Tajikistan" "Tanzania"
## [109] "Thailand" "Togo" "Trinidad and Tobago"
## [112] "Turkey" "Uganda" "Ukraine"
## [115] "United Arab Emirates" "United Kingdom" "United States"
## [118] "Uruguay" "Venezuela" "Vietnam"
## [121] "Yemen" "Zambia"

different2 <- **anti_join**(results, world_map, by = "region")
**print**(**levels**(**factor**(different2**$**region )))

## [1] "Antigua and Barbuda" "Czechia" "North Macedonia"
## [4] "Trinidad and Tobago" "United Kingdom" "United States"

world_map <- **map_data**("world")
 world_map<-world_map **%>%** *#here change all county names that do not match in the world map to the data terminology*
 **mutate**(region = **ifelse**(region **==**"USA", "United States", region))
 world_map<-world_map **%>%** *#here change all county names that do not match in the world map to the data terminology*
 **mutate**(region = **ifelse**(region **==**"UK", "United Kingdom", region))
 world_map<-world_map **%>%** *#here change all county names that do not match in the world map to the data terminology*
 **mutate**(region = **ifelse**(region **==**"Trinidad", "Trinidad and Tobago", region))
 world_map<-world_map **%>%** *#here change all county names that do not match in the world map to the data terminology*
 **mutate**(region = **ifelse**(region **==**"Macedonia", "North Macedonia", region))
 world_map<-world_map **%>%** *#here change all county names that do not match in the world map to the data terminology*
 **mutate**(region = **ifelse**(region **==**"Czech Republic", "Czechia", region))
 world_map<-world_map **%>%** *#here change all county names that do not match in the world map to the data terminology*
 **mutate**(region = **ifelse**(region **==**"Antigua", "Antigua and Barbuda", region))

corrmap <- **full_join**( world_map, results, by = "region")


 p1<-**ggplot**(corrmap, **aes**(long, lat, group=group))**+**
 **geom_polygon**(**aes**(fill = retail_and_recreation_percent_change_from_baseline ), color = "white")**+**
 **scale_fill_viridis_c**(option = "C") **+** **ggtitle**("Retail and recreation - lag") **+** **labs**(fill = "Lag to maximum correlation in days")
p2<- **ggplot**(corrmap, **aes**(long, lat, group=group))**+**
 **geom_polygon**(**aes**(fill = V1 ), color = "white")**+**
 **scale_fill_viridis_c**(option = "C") **+** **ggtitle**("Retail and recreation -strength of correlation") **+** **labs**(fill = "Kendall`s Tau")

 p3<-**ggplot**(corrmap, **aes**(long, lat, group=group))**+**
 **geom_polygon**(**aes**(fill = grocery_and_pharmacy_percent_change_from_baseline), color = "white")**+**
 **scale_fill_viridis_c**(option = "C") **+** **ggtitle**("Grocery and pharmacy- lag") **+** **labs**(fill = "Lag to maximum correlation in days")
p4<- **ggplot**(corrmap, **aes**(long, lat, group=group))**+**
 **geom_polygon**(**aes**(fill = V2 ), color = "white")**+**
 **scale_fill_viridis_c**(option = "C") **+** **ggtitle**("Grocery and pharmacy- strength of correlation") **+** **labs**(fill = "Kendall`s Tau")

 p5<-**ggplot**(corrmap, **aes**(long, lat, group=group))**+**
 **geom_polygon**(**aes**(fill = parks_percent_change_from_baseline), color = "white")**+**
 **scale_fill_viridis_c**(option = "C") **+** **ggtitle**("Parks- lag") **+** **labs**(fill = "Lag to maximum correlation in days")
p6<- **ggplot**(corrmap, **aes**(long, lat, group=group))**+**
 **geom_polygon**(**aes**(fill = V3 ), color = "white")**+**
 **scale_fill_viridis_c**(option = "C") **+** **ggtitle**("Parks- strength of correlation") **+** **labs**(fill = "Kendall`s Tau")
 p7<-**ggplot**(corrmap, **aes**(long, lat, group=group))**+**
 **geom_polygon**(**aes**(fill = transit_stations_percent_change_from_baseline), color = "white")**+**
 **scale_fill_viridis_c**(option = "C") **+** **ggtitle**("Transit stations- lag") **+** **labs**(fill = "Lag to maximum correlation in days")
p8<- **ggplot**(corrmap, **aes**(long, lat, group=group))**+**
 **geom_polygon**(**aes**(fill = V4 ), color = "white")**+**
 **scale_fill_viridis_c**(option = "C") **+** **ggtitle**("Transit stations- strength of correlation") **+** **labs**(fill = "Kendall`s Tau")
p9<-**ggplot**(corrmap, **aes**(long, lat, group=group))**+**
 **geom_polygon**(**aes**(fill = workplaces_percent_change_from_baseline), color = "white")**+**
 **scale_fill_viridis_c**(option = "C") **+** **ggtitle**("Workplace- lag") **+** **labs**(fill = "Lag to maximum correlation in days")
p10<- **ggplot**(corrmap, **aes**(long, lat, group=group))**+**
 **geom_polygon**(**aes**(fill = V5 ), color = "white")**+**
 **scale_fill_viridis_c**(option = "C") **+** **ggtitle**("Workplace- strength of correlation") **+** **labs**(fill = "Kendall`s Tau")
p11<-**ggplot**(corrmap, **aes**(long, lat, group=group))**+**
 **geom_polygon**(**aes**(fill = residential_percent_change_from_baseline), color = "white")**+**
 **scale_fill_viridis_c**(option = "C") **+** **ggtitle**("Residential- lag") **+** **labs**(fill = "Lag to maximum correlation in days")
p12<- **ggplot**(corrmap, **aes**(long, lat, group=group))**+**
 **geom_polygon**(**aes**(fill = V6), color = "white")**+**
 **scale_fill_viridis_c**(option = "C") **+** **ggtitle**("Residential- strength of correlation") **+** **labs**(fill = "Kendall`s Tau")


**library**(ggpubr)
lags<- **ggarrange**(p1, p3, p5, p7, p9, p11 , ncol = 2, nrow = 3)
lags


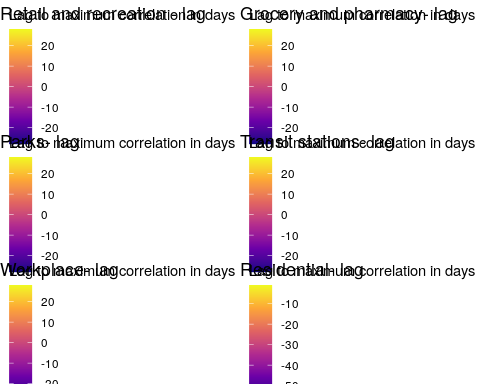


taus<- **ggarrange**(p2, p4, p6, p8, p10, p12, ncol = 2, nrow = 3)
taus


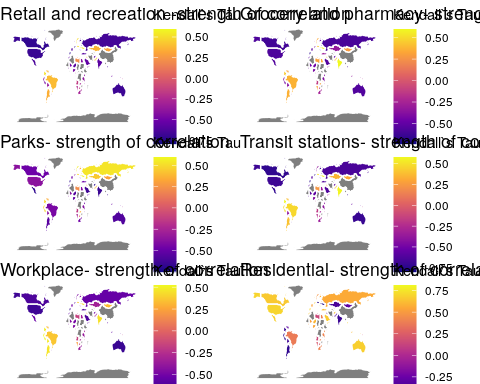


**summary**(results)

## country_region Continent
## Length:122 Length:122
## Class :character Class :character
## Mode :character Mode :character
##
##
##
## retail_and_recreation_percent_change_from_baseline
## Min. :-28.0000
## 1st Qu.:-15.7500
## Median : 1.0000
## Mean : -0.7705
## 3rd Qu.: 11.7500
## Max. : 28.0000
## grocery_and_pharmacy_percent_change_from_baseline
## Min. :-28.0000
## 1st Qu.:-20.5000
## Median : 2.0000
## Mean : -0.6721
## 3rd Qu.: 10.7500
## Max. : 28.0000
## parks_percent_change_from_baseline
## Min. :-28.000
## 1st Qu.:-24.000
## Median : -2.000
## Mean : -2.566
## 3rd Qu.: 13.000
## Max. : 28.000
## transit_stations_percent_change_from_baseline
## Min. :-28.00000
## 1st Qu.:-14.50000
## Median : 1.00000
## Mean : 0.02459
## 3rd Qu.: 10.00000
## Max. : 28.00000
## workplaces_percent_change_from_baseline
## Min. :-28.00000
## 1st Qu.:-15.50000
## Median : 1.00000
## Mean : -0.08197
## 3rd Qu.: 11.00000
## Max. : 28.00000
## residential_percent_change_from_baseline V1 V2
## Min. :-57.00 Min. :-0.7978 Min. :-0.7393
## 1st Qu.:-44.50 1st Qu.:-0.6231 1st Qu.:-0.5186
## Median :-28.00 Median :-0.4705 Median :-0.3979
## Mean :-29.08 Mean :-0.3464 Mean :-0.2611
## 3rd Qu.:-18.00 3rd Qu.:-0.2582 3rd Qu.:-0.1852
## Max. : -1.00 Max. : 0.5947 Max. : 0.5978
## V3 V4 V5 V6
## Min. :-0.7520 Min. :-0.8010 Min. :-0.7908 Min. :-0.5182
## 1st Qu.:-0.5052 1st Qu.:-0.6432 1st Qu.:-0.5810 1st Qu.: 0.2507
## Median :-0.3856 Median :-0.4386 Median :-0.4550 Median : 0.4881
## Mean :-0.2289 Mean :-0.3451 Mean :-0.3100 Mean : 0.3439
## 3rd Qu.: 0.2248 3rd Qu.:-0.2335 3rd Qu.:-0.1942 3rd Qu.: 0.6293
## Max. : 0.5945 Max. : 0.5785 Max. : 0.5227 Max. : 0.8121
## region
## Length:122
## Class :character
## Mode :character
##
##
##

**write.csv**(results, "resultscovidcorrgmd.csv")

#mobility data
gmr <- read_csv("Downloads/Global_Mobility_Report(2).csv", col_types = cols(date = col_date(format = "%Y-%m-%d")))


### Loading country data

countries <- fread("http://download.geonames.org/export/dump/countryInfo.txt", skip = "ISO3", na.strings = "")
names(countries)[c(1,5, 9)] <- c("geo", "Country.Region", "Continent")
countries$lang <- sapply(strsplit(sapply(strsplit(countries$Languages, ","), `[`, 1), "-"), `[`, 1)
countries$translated <- "Coronavirus"

### Obtaining the case numbers

jhu_url <- paste0("https://raw.githubusercontent.com/CSSEGISandData/COVID-19/master/csse_covid_19_data/",
 "csse_covid_19_time_series/time_series_covid19_confirmed_global.csv")
CaseData <- fread(jhu_url, check.names = TRUE)
CaseData$Province.State[ CaseData$Province.State=="" ] <- CaseData$Country.Region[ CaseData$Province.State=="" ]
CaseData <- melt(CaseData, id.vars = 1:4, variable.name = "Date", variable.factor = FALSE)
CaseData$Date <- as.Date( substring(CaseData$Date, 2), format = "%m.%d.%y" )
CaseData <- CaseData[ , .(CumCaseNumber = sum(value)), .(Country.Region, Date)][order(Country.Region, Date)]
CaseData <- CaseData[ ,.(date = Date[-1], CumCaseNumber = CumCaseNumber[-1], IncCaseNumber = diff(CumCaseNumber)),
 .(Country.Region)]

CaseData[Country.Region=="US"]$Country.Region <- "United States"
CaseData <- merge(CaseData,countries[,c("Country.Region", "geo", "Continent")])
CaseData$country_region<-CaseData$Country.Region

CaseData$IncCaseNumber<-ifelse(CaseData$IncCaseNumber<0, 0, CaseData$IncCaseNumber )
write.csv(CaseData, "CaseNumbberseptember0110.csv")


allmerged<-merge(gmr, CaseData, by=c("country_region", "date"))
all<-allmerged
all$NumDate <- as.numeric(all$date)-min(as.numeric(all$date))
data<- subset(all, is.na(all$sub_region_1)==TRUE)
data$IncCaseNumber<-ifelse(data$IncCaseNumber<0, 0, data$IncCaseNumber )

data<-data[c(1,2, 9:14, 17,20)]
summary(data)

## country_region date
## Length:38439 Min. :2020-02-15
## Class :character 1st Qu.:2020-04-06
## Mode :character Median :2020-05-28
## Mean :2020-05-27
## 3rd Qu.:2020-07-19
## Max. :2020-09-08
##
## retail_and_recreation_percent_change_from_baseline
## Min. :-98.00
## 1st Qu.:-47.00
## Median :-21.00
## Mean :-26.45
## 3rd Qu.: -4.00
## Max. : 66.00
## NA's :305
## grocery_and_pharmacy_percent_change_from_baseline
## Min. :-98.00
## 1st Qu.:-24.00
## Median : -7.00
## Mean :-11.98
## 3rd Qu.: 3.00
## Max. :100.00
## NA's :350
## parks_percent_change_from_baseline
## Min. :-97.000
## 1st Qu.:-35.000
## Median :-11.000
## Mean : -2.264
## 3rd Qu.: 11.000
## Max. :517.000
## NA's :333
## transit_stations_percent_change_from_baseline
## Min. :-96.00
## 1st Qu.:-51.00
## Median :-30.00
## Mean :-31.12
## 3rd Qu.: -9.00
## Max. : 67.00
## NA's :214
## workplaces_percent_change_from_baseline
## Min. :-93.00
## 1st Qu.:-39.00
## Median :-24.00
## Mean :-24.97
## 3rd Qu.: -8.00
## Max. : 80.00
## NA's :60
## residential_percent_change_from_baseline IncCaseNumber NumDate
## Min. :-35.00 Min. : 0 Min. : 0.0
## 1st Qu.: 2.00 1st Qu.: 2 1st Qu.: 51.0
## Median : 10.00 Median : 72 Median :103.0
## Mean : 10.82 Mean : 1297 Mean :102.9
## 3rd Qu.: 18.00 3rd Qu.: 637 3rd Qu.:155.0
## Max. : 55.00 Max. :90802 Max. :206.0
## NA's :534

data<-na.omit(data)


library(dlnm)

## This is dlnm 2.4.2. For details: help(dlnm) and vignette('dlnmOverview').

library(lme4)

## Loading required package: Matrix

library(lmerTest)

##
## Attaching package: 'lmerTest'

## The following object is masked from 'package:lme4':
##
## lmer

## The following object is masked from 'package:stats':
##
## step

library(gamm4)

## Loading required package: mgcv

## Loading required package: nlme

##
## Attaching package: 'nlme'

## The following object is masked from 'package:lme4':
##
## lmList

## This is mgcv 1.8-33. For overview type 'help("mgcv-package")'.

## This is gamm4 0.2-6

library(dplyr)

##
## Attaching package: 'dplyr'

## The following object is masked from 'package:nlme':
##
## collapse

## The following objects are masked from 'package:data.table':
##
## between, first, last

## The following objects are masked from 'package:stats':
##
## filter, lag

## The following objects are masked from 'package:base':
##
## intersect, setdiff, setequal, union

data$country_region<-factor(data$country_region)

datat<-data %>%
 filter(date<= "2020-06-19")
datav<-data %>%
 filter(date> "2020-06-19")

cb1 <- crossbasis(datat$grocery_and_pharmacy_percent_change_from_baseline , lag=14, argvar=list(fun="bs"),arglag=list(df=5), group=datat$country_region)
cb2 <- crossbasis(datat$retail_and_recreation_percent_change_from_baseline , lag=14, argvar=list(fun="bs"),arglag=list(df=5), group=datat$country_region)
cb4 <- crossbasis(datat$transit_stations_percent_change_from_baseline , lag=14, argvar=list(fun="bs"),arglag=list(df=5), group=datat$country_region)
cb5 <- crossbasis(datat$workplaces_percent_change_from_baseline , lag=14, argvar=list(fun="bs"),arglag=list(df=5), group=datat$country_region)
cb6 <- crossbasis(datat$residential_percent_change_from_baseline , lag=14, argvar=list(fun="bs"),arglag=list(df=5), group=datat$country_region)

 #make data identical to the dataset with crossbasis (deleting the first 14 observations in all countries)
datam<-datat %>%
 group_by(country_region) %>%
 slice(-c(1:14))
set.seed(2020)
fittweedie<-gam(IncCaseNumber~s(NumDate) + s(country_region, bs="re"), data=datam, family="tw")
summary(fittweedie) # just to estimate p , with gam instead of gamm model ######

##
## Family: Tweedie(p=1.656)
## Link function: log
##
## Formula:
## IncCaseNumber ~ s(NumDate) + s(country_region, bs = "re")
##
## Parametric coefficients:
## Estimate Std. Error t value Pr(>|t|)
## (Intercept) 3.2818 0.2192 14.97 <2e-16 ***
## ---
## Signif. codes: 0 '***' 0.001 '**' 0.01 '*' 0.05 '.' 0.1 ' ' 1
##
## Approximate significance of smooth terms:
## edf Ref.df F p-value
## s(NumDate) 8.896 8.994 2675.8 <2e-16 ***
## s(country_region) 121.568 122.000 647.3 <2e-16 ***
## ---
## Signif. codes: 0 '***' 0.001 '**' 0.01 '*' 0.05 '.' 0.1 ' ' 1
##
## R-sq.(adj) = 0.664 Deviance explained = 83.9%
## -REML = 1.0975e+05 Scale est. = 6.5753 n = 21311

BIC(fittweedie)

## [1] 220034.9

fit1 <- gamm(IncCaseNumber~s(NumDate), random=list(country_region=~1), data=datam, family=Tweedie(p=1.656)) ####modify power functin accordingly

##
## Maximum number of PQL iterations: 20

## iteration 1

## iteration 2

## iteration 3

## iteration 4

## iteration 5

## iteration 6

## iteration 7

fit2 <- gamm(IncCaseNumber~s(NumDate)+s(retail_and_recreation_percent_change_from_baseline)+s(grocery_and_pharmacy_percent_change_from_baseline)+ s(transit_stations_percent_change_from_baseline)+s(workplaces_percent_change_from_baseline+residential_percent_change_from_baseline), random=list(country_region=~1), data=datam, family=Tweedie(p=1.656))

##
## Maximum number of PQL iterations: 20

## iteration 1

## iteration 2

## iteration 3

## iteration 4

## iteration 5

## iteration 6

## iteration 7

fitcb<- gamm(IncCaseNumber~s(NumDate) + cb1 + cb2 + cb4 + cb5 + cb6, random=list(country_region=~1), data=datat, family=Tweedie(p=1.656))

##
## Maximum number of PQL iterations: 20

## iteration 1

## iteration 2

## iteration 3

## iteration 4

## iteration 5

## iteration 6

## iteration 7

## iteration 8

## iteration 9

## iteration 10

## iteration 11

BIC(fit1$lme)

## [1] 79802.47

BIC(fit2$lme)

## [1] 79096.29

BIC(fitcb$lme)

## [1] 80286.14

summary(fit1$lme)

## Linear mixed-effects model fit by maximum likelihood
## Data: data
## AIC BIC logLik
## 79762.63 79802.47 -39876.32
##
## Random effects:
## Formula: ~Xr - 1 | g
## Structure: pdIdnot
## Xr1 Xr2 Xr3 Xr4 Xr5 Xr6 Xr7 Xr8
## StdDev: 8.205045 8.205045 8.205045 8.205045 8.205045 8.205045 8.205045 8.205045
##
## Formula: ~1 | country_region %in% g
## (Intercept) Residual
## StdDev: 2.412045 3.046468
##
## Variance function:
## Structure: fixed weights
## Formula: ~invwt
## Fixed effects: list(fixed)
## Value Std.Error DF t-value p-value
## X(Intercept) 3.286403 0.2181920 21187 15.06198 0
## Xs(NumDate)Fx1 8.575054 0.5385667 21187 15.92199 0
## Correlation:
## X(Int)
## Xs(NumDate)Fx1 -0.016
##
## Standardized Within-Group Residuals:
## Min Q1 Med Q3 Max
## -1.6850292 -0.4765888 -0.2406981 0.2216138 22.4753197
##
## Number of Observations: 21311
## Number of Groups:
## g country_region %in% g
## 1 123

summary(fit2$lme)

## Linear mixed-effects model fit by maximum likelihood
## Data: data
## AIC BIC logLik
## 78992.72 79096.29 -39483.36
##
## Random effects:
## Formula: ~Xr - 1 | g
## Structure: pdIdnot
## Xr1 Xr2 Xr3 Xr4 Xr5 Xr6 Xr7 Xr8
## StdDev: 20.31991 20.31991 20.31991 20.31991 20.31991 20.31991 20.31991 20.31991
##
## Formula: ~Xr.0 - 1 | g.0 %in% g
## Structure: pdIdnot
## Xr.01 Xr.02 Xr.03 Xr.04 Xr.05 Xr.06 Xr.07 Xr.08
## StdDev: 4.320994 4.320994 4.320994 4.320994 4.320994 4.320994 4.320994 4.320994
##
## Formula: ~Xr.1 - 1 | g.1 %in% g.0 %in% g
## Structure: pdIdnot
## Xr.11 Xr.12 Xr.13 Xr.14 Xr.15 Xr.16 Xr.17 Xr.18
## StdDev: 4.58942 4.58942 4.58942 4.58942 4.58942 4.58942 4.58942 4.58942
##
## Formula: ~Xr.2 - 1 | g.2 %in% g.1 %in% g.0 %in% g
## Structure: pdIdnot
## Xr.21 Xr.22 Xr.23 Xr.24 Xr.25 Xr.26 Xr.27 Xr.28
## StdDev: 1.560062 1.560062 1.560062 1.560062 1.560062 1.560062 1.560062 1.560062
##
## Formula: ~Xr.3 - 1 | g.3 %in% g.2 %in% g.1 %in% g.0 %in% g
## Structure: pdIdnot
## Xr.31 Xr.32 Xr.33 Xr.34 Xr.35 Xr.36 Xr.37 Xr.38
## StdDev: 2.60778 2.60778 2.60778 2.60778 2.60778 2.60778 2.60778 2.60778
##
## Formula: ~1 | country_region %in% g.3 %in% g.2 %in% g.1 %in% g.0 %in% g
## (Intercept) Residual
## StdDev: 2.150661 2.912942
##
## Variance function:
## Structure: fixed weights
## Formula: ~invwt
## Fixed effects: list(fixed)
## Value Std.Error
## X(Intercept) 3.136555 0.1947519
## Xs(NumDate)Fx1 14.876413 0.6673706
## Xs(retail_and_recreation_percent_change_from_baseline)Fx1 -1.335009 0.5947718
## Xs(grocery_and_pharmacy_percent_change_from_baseline)Fx1 0.333238 0.3623668
## Xs(transit_stations_percent_change_from_baseline)Fx1 -0.325932 0.3616842
## Xs(workplaces_percent_change_from_baseline)Fx1 -0.417412 0.5124154
## DF t-value
## X(Intercept) 21183 16.105393
## Xs(NumDate)Fx1 21183 22.291083
## Xs(retail_and_recreation_percent_change_from_baseline)Fx1 21183 -2.244573
## Xs(grocery_and_pharmacy_percent_change_from_baseline)Fx1 21183 0.919616
## Xs(transit_stations_percent_change_from_baseline)Fx1 21183 -0.901151
## Xs(workplaces_percent_change_from_baseline)Fx1 21183 -0.814596
## p-value
## X(Intercept) 0.0000
## Xs(NumDate)Fx1 0.0000
## Xs(retail_and_recreation_percent_change_from_baseline)Fx1 0.0248
## Xs(grocery_and_pharmacy_percent_change_from_baseline)Fx1 0.3578
## Xs(transit_stations_percent_change_from_baseline)Fx1 0.3675
## Xs(workplaces_percent_change_from_baseline)Fx1 0.4153
## Correlation:
## X(Int) X(ND)F
## Xs(NumDate)Fx1 -0.028
## Xs(retail_and_recreation_percent_change_from_baseline)Fx1 0.001 -0.029
## Xs(grocery_and_pharmacy_percent_change_from_baseline)Fx1 0.001 -0.005
## Xs(transit_stations_percent_change_from_baseline)Fx1 0.000 0.004
## Xs(workplaces_percent_change_from_baseline)Fx1 0.001 -0.005
## Xs(r______)F1
## Xs(NumDate)Fx1
## Xs(retail_and_recreation_percent_change_from_baseline)Fx1
## Xs(grocery_and_pharmacy_percent_change_from_baseline)Fx1 -0.075
## Xs(transit_stations_percent_change_from_baseline)Fx1 -0.167
## Xs(workplaces_percent_change_from_baseline)Fx1 0.001
## Xs(g______)F1
## Xs(NumDate)Fx1
## Xs(retail_and_recreation_percent_change_from_baseline)Fx1
## Xs(grocery_and_pharmacy_percent_change_from_baseline)Fx1
## Xs(transit_stations_percent_change_from_baseline)Fx1 -0.032
## Xs(workplaces_percent_change_from_baseline)Fx1 -0.010
## X(_____)
## Xs(NumDate)Fx1
## Xs(retail_and_recreation_percent_change_from_baseline)Fx1
## Xs(grocery_and_pharmacy_percent_change_from_baseline)Fx1
## Xs(transit_stations_percent_change_from_baseline)Fx1
## Xs(workplaces_percent_change_from_baseline)Fx1 -0.007
##
## Standardized Within-Group Residuals:
## Min Q1 Med Q3 Max
## -1.5295620 -0.4709505 -0.2391705 0.2085456 24.0972046
##
## Number of Observations: 21311
## Number of Groups:
## g
## 1
## g.0 %in% g
## 1
## g.1 %in% g.0 %in% g
## 1
## g.2 %in% g.1 %in% g.0 %in% g
## 1
## g.3 %in% g.2 %in% g.1 %in% g.0 %in% g
## 1
## country_region %in% g.3 %in% g.2 %in% g.1 %in% g.0 %in% g
## 123

ranef(fit2$lme)

## Level: g
## Xr1 Xr2 Xr3 Xr4 Xr5 Xr6 Xr7 Xr8
## 1 -2.645335 -17.34849 -8.200305 17.14152 -24.29321 14.39787 -19.26648 -38.23257
##
## Level: g.0 %in% g
## Xr.01 Xr.02 Xr.03 Xr.04 Xr.05 Xr.06 Xr.07 Xr.08
## 1/1 -0.6495366 -4.323514 -6.4993 -3.740349 3.320843 4.614124 3.131036 4.916534
##
## Level: g.1 %in% g.0 %in% g
## Xr.11 Xr.12 Xr.13 Xr.14 Xr.15 Xr.16 Xr.17
## 1/1/1 -0.511122 -8.843325 4.893857 -6.596312 -2.704825 0.03800879 -0.5052104
## Xr.18
## 1/1/1 -0.9439142
##
## Level: g.2 %in% g.1 %in% g.0 %in% g
## Xr.21 Xr.22 Xr.23 Xr.24 Xr.25 Xr.26 Xr.27
## 1/1/1/1 -1.79714 0.3400537 -1.035872 1.309102 1.588855 2.022742 1.313216
## Xr.28
## 1/1/1/1 0.7984307
##
## Level: g.3 %in% g.2 %in% g.1 %in% g.0 %in% g
## Xr.31 Xr.32 Xr.33 Xr.34 Xr.35 Xr.36 Xr.37
## 1/1/1/1/1 -1.778276 1.37795 2.905501 -2.577511 -1.935447 -4.35463 0.5206838
## Xr.38
## 1/1/1/1/1 -0.3468908
##
## Level: country_region %in% g.3 %in% g.2 %in% g.1 %in% g.0 %in% g
## (Intercept)
## 1/1/1/1/1/Afghanistan 0.82515705
## 1/1/1/1/1/Angola -3.45004752
## 1/1/1/1/1/Antigua and Barbuda -4.84650933
## 1/1/1/1/1/Argentina 0.55553471
## 1/1/1/1/1/Australia 0.99466991
## 1/1/1/1/1/Austria 0.87086586
## 1/1/1/1/1/Bahrain 0.84307958
## 1/1/1/1/1/Bangladesh 2.27287969
## 1/1/1/1/1/Barbados -3.95456454
## 1/1/1/1/1/Belarus 2.60209951
## 1/1/1/1/1/Belgium 1.82565805
## 1/1/1/1/1/Belize -5.94335234
## 1/1/1/1/1/Benin -1.30841483
## 1/1/1/1/1/Bolivia 0.49063069
## 1/1/1/1/1/Bosnia and Herzegovina -0.41730139
## 1/1/1/1/1/Botswana -3.67544032
## 1/1/1/1/1/Brazil 4.28496046
## 1/1/1/1/1/Bulgaria -0.38520228
## 1/1/1/1/1/Burkina Faso -0.73972329
## 1/1/1/1/1/Cambodia -2.80167204
## 1/1/1/1/1/Cameroon 0.87493408
## 1/1/1/1/1/Canada 2.52753550
## 1/1/1/1/1/Chile 3.02937654
## 1/1/1/1/1/Colombia 1.57244002
## 1/1/1/1/1/Costa Rica -1.22693919
## 1/1/1/1/1/Croatia -0.76480507
## 1/1/1/1/1/Czechia 0.63790108
## 1/1/1/1/1/Denmark 1.66359570
## 1/1/1/1/1/Dominican Republic 0.76823382
## 1/1/1/1/1/Ecuador 1.65234907
## 1/1/1/1/1/Egypt 1.26778044
## 1/1/1/1/1/El Salvador -1.12195570
## 1/1/1/1/1/Estonia -0.59371712
## 1/1/1/1/1/Fiji -4.72664807
## 1/1/1/1/1/Finland 0.35392354
## 1/1/1/1/1/France 2.74662230
## 1/1/1/1/1/Gabon -0.43969863
## 1/1/1/1/1/Georgia -1.15675223
## 1/1/1/1/1/Germany 3.77957291
## 1/1/1/1/1/Ghana 0.98195091
## 1/1/1/1/1/Greece -0.82071769
## 1/1/1/1/1/Guatemala -0.24563662
## 1/1/1/1/1/Haiti -0.49402759
## 1/1/1/1/1/Honduras -0.27096873
## 1/1/1/1/1/Hungary -0.05668553
## 1/1/1/1/1/India 2.98768726
## 1/1/1/1/1/Indonesia 1.70184924
## 1/1/1/1/1/Iraq 1.09860298
## 1/1/1/1/1/Ireland 0.44911657
## 1/1/1/1/1/Israel 0.80077454
## 1/1/1/1/1/Italy 3.21306594
## 1/1/1/1/1/Jamaica -2.08375966
## 1/1/1/1/1/Japan 1.77953652
## 1/1/1/1/1/Jordan -2.23877282
## 1/1/1/1/1/Kazakhstan 1.18078171
## 1/1/1/1/1/Kenya -0.31688688
## 1/1/1/1/1/Kuwait 1.33621901
## 1/1/1/1/1/Kyrgyzstan -0.68374040
## 1/1/1/1/1/Laos -4.90661153
## 1/1/1/1/1/Latvia -0.98396716
## 1/1/1/1/1/Lebanon -1.41848885
## 1/1/1/1/1/Libya -2.34508236
## 1/1/1/1/1/Lithuania -1.12872125
## 1/1/1/1/1/Luxembourg -0.87803005
## 1/1/1/1/1/Malaysia 0.11260757
## 1/1/1/1/1/Mali -0.28760153
## 1/1/1/1/1/Malta -2.16236755
## 1/1/1/1/1/Mauritius -3.02813113
## 1/1/1/1/1/Mexico 2.43013965
## 1/1/1/1/1/Moldova 0.78446551
## 1/1/1/1/1/Mongolia -1.94205675
## 1/1/1/1/1/Morocco -0.21154634
## 1/1/1/1/1/Mozambique -1.80544464
## 1/1/1/1/1/Namibia -4.22976681
## 1/1/1/1/1/Nepal -0.29508093
## 1/1/1/1/1/Netherlands 2.30098227
## 1/1/1/1/1/New Zealand -1.84767497
## 1/1/1/1/1/Nicaragua -1.81590615
## 1/1/1/1/1/Niger -0.87480667
## 1/1/1/1/1/Nigeria 1.02297189
## 1/1/1/1/1/North Macedonia -0.58719893
## 1/1/1/1/1/Norway 1.21939899
## 1/1/1/1/1/Oman 0.89436259
## 1/1/1/1/1/Pakistan 3.17893161
## 1/1/1/1/1/Panama 0.44775797
## 1/1/1/1/1/Papua New Guinea -4.14588082
## 1/1/1/1/1/Paraguay -1.78291266
## 1/1/1/1/1/Peru 2.47618468
## 1/1/1/1/1/Philippines 0.65991590
## 1/1/1/1/1/Poland 1.76727126
## 1/1/1/1/1/Portugal 1.35506275
## 1/1/1/1/1/Qatar 2.03709950
## 1/1/1/1/1/Romania 1.23983141
## 1/1/1/1/1/Russia 4.25094421
## 1/1/1/1/1/Rwanda -2.06032742
## 1/1/1/1/1/Saudi Arabia 1.96760536
## 1/1/1/1/1/Senegal -0.20516455
## 1/1/1/1/1/Serbia 0.75320461
## 1/1/1/1/1/Singapore 1.13593352
## 1/1/1/1/1/Slovakia -1.26490194
## 1/1/1/1/1/Slovenia -0.67846650
## 1/1/1/1/1/South Africa 1.85103903
## 1/1/1/1/1/Spain 2.76847864
## 1/1/1/1/1/Sri Lanka -1.20330048
## 1/1/1/1/1/Sweden 3.06343627
## 1/1/1/1/1/Switzerland 1.64883278
## 1/1/1/1/1/Tajikistan -0.14664654
## 1/1/1/1/1/Tanzania -2.08203956
## 1/1/1/1/1/Thailand -0.18399797
## 1/1/1/1/1/Togo -1.98610547
## 1/1/1/1/1/Trinidad and Tobago -3.66118591
## 1/1/1/1/1/Turkey 2.84148940
## 1/1/1/1/1/Uganda -2.31370354
## 1/1/1/1/1/Ukraine 1.65702526
## 1/1/1/1/1/United Arab Emirates 1.15915934
## 1/1/1/1/1/United Kingdom 3.11615380
## 1/1/1/1/1/United States 5.97640294
## 1/1/1/1/1/Uruguay -1.67223723
## 1/1/1/1/1/Venezuela -0.92658152
## 1/1/1/1/1/Vietnam -1.53492182
## 1/1/1/1/1/Yemen -1.34686890
## 1/1/1/1/1/Zambia -0.80761949
## 1/1/1/1/1/Zimbabwe -2.57078815

summary(fitcb$lme)

## Linear mixed-effects model fit by maximum likelihood
## Data: data
## AIC BIC logLik
## 79648.78 80286.14 -39744.39
##
## Random effects:
## Formula: ~Xr - 1 | g
## Structure: pdIdnot
## Xr1 Xr2 Xr3 Xr4 Xr5 Xr6 Xr7 Xr8
## StdDev: 25.28211 25.28211 25.28211 25.28211 25.28211 25.28211 25.28211 25.28211
##
## Formula: ~1 | country_region %in% g
## (Intercept) Residual
## StdDev: 2.105007 2.934869
##
## Variance function:
## Structure: fixed weights
## Formula: ~invwt
## Fixed effects: list(fixed)
## Value Std.Error DF t-value p-value
## X(Intercept) 5.020970 0.4592959 21112 10.931886 0.0000
## Xcb1v1.l1 0.374865 0.3721891 21112 1.007189 0.3139
## Xcb1v1.l2 -0.255013 0.3618270 21112 -0.704794 0.4809
## Xcb1v1.l3 0.143252 0.3475072 21112 0.412226 0.6802
## Xcb1v1.l4 1.783361 0.2565186 21112 6.952170 0.0000
## Xcb1v1.l5 -1.160218 0.3261709 21112 -3.557087 0.0004
## Xcb1v2.l1 0.944672 0.3661131 21112 2.580273 0.0099
## Xcb1v2.l2 0.646309 0.3600314 21112 1.795146 0.0726
## Xcb1v2.l3 -0.643013 0.3467898 21112 -1.854187 0.0637
## Xcb1v2.l4 0.881516 0.2438502 21112 3.614987 0.0003
## Xcb1v2.l5 -2.082041 0.3049360 21112 -6.827797 0.0000
## Xcb1v3.l1 -1.015948 0.4887558 21112 -2.078641 0.0377
## Xcb1v3.l2 -0.474631 0.4783082 21112 -0.992311 0.3211
## Xcb1v3.l3 -1.342914 0.4596649 21112 -2.921506 0.0035
## Xcb1v3.l4 2.870586 0.3304954 21112 8.685706 0.0000
## Xcb1v3.l5 -2.247276 0.4112231 21112 -5.464859 0.0000
## Xcb2v1.l1 -0.193364 0.4257012 21112 -0.454225 0.6497
## Xcb2v1.l2 0.111292 0.4031299 21112 0.276071 0.7825
## Xcb2v1.l3 0.591788 0.3934023 21112 1.504281 0.1325
## Xcb2v1.l4 -1.773234 0.2747198 21112 -6.454701 0.0000
## Xcb2v1.l5 2.713757 0.3302797 21112 8.216544 0.0000
## Xcb2v2.l1 -0.427686 0.3300349 21112 -1.295882 0.1950
## Xcb2v2.l2 -0.362205 0.3128055 21112 -1.157925 0.2469
## Xcb2v2.l3 -0.235648 0.2948090 21112 -0.799324 0.4241
## Xcb2v2.l4 0.718468 0.2109788 21112 3.405404 0.0007
## Xcb2v2.l5 0.144222 0.2595512 21112 0.555659 0.5785
## Xcb2v3.l1 -0.133090 0.5150176 21112 -0.258418 0.7961
## Xcb2v3.l2 0.287773 0.4761248 21112 0.604407 0.5456
## Xcb2v3.l3 1.654037 0.4520969 21112 3.658590 0.0003
## Xcb2v3.l4 -4.603246 0.3150966 21112 -14.608999 0.0000
## Xcb2v3.l5 4.743748 0.3740915 21112 12.680716 0.0000
## Xcb4v1.l1 -0.465572 0.4425798 21112 -1.051950 0.2928
## Xcb4v1.l2 -0.515572 0.4308014 21112 -1.196773 0.2314
## Xcb4v1.l3 -0.337342 0.4171748 21112 -0.808634 0.4187
## Xcb4v1.l4 -1.231644 0.2959405 21112 -4.161796 0.0000
## Xcb4v1.l5 0.281375 0.3503950 21112 0.803024 0.4220
## Xcb4v2.l1 -0.017281 0.3836048 21112 -0.045048 0.9641
## Xcb4v2.l2 0.266413 0.3688443 21112 0.722290 0.4701
## Xcb4v2.l3 0.953939 0.3558674 21112 2.680604 0.0074
## Xcb4v2.l4 0.146267 0.2447881 21112 0.597525 0.5502
## Xcb4v2.l5 1.221864 0.3058759 21112 3.994639 0.0001
## Xcb4v3.l1 -0.384795 0.5819877 21112 -0.661174 0.5085
## Xcb4v3.l2 -0.640216 0.5525944 21112 -1.158564 0.2466
## Xcb4v3.l3 -0.587180 0.5326345 21112 -1.102408 0.2703
## Xcb4v3.l4 -0.583482 0.3787949 21112 -1.540363 0.1235
## Xcb4v3.l5 -0.337323 0.4448719 21112 -0.758247 0.4483
## Xcb5v1.l1 -0.550332 0.4952625 21112 -1.111193 0.2665
## Xcb5v1.l2 0.435913 0.4862241 21112 0.896526 0.3700
## Xcb5v1.l3 0.683760 0.4611893 21112 1.482603 0.1382
## Xcb5v1.l4 -1.564225 0.3379852 21112 -4.628088 0.0000
## Xcb5v1.l5 0.028591 0.4099131 21112 0.069750 0.9444
## Xcb5v2.l1 0.362309 0.5902433 21112 0.613831 0.5393
## Xcb5v2.l2 -0.350162 0.5703016 21112 -0.613995 0.5392
## Xcb5v2.l3 -1.448650 0.5459400 21112 -2.653497 0.0080
## Xcb5v2.l4 3.124060 0.4028864 21112 7.754196 0.0000
## Xcb5v2.l5 -0.932454 0.5025987 21112 -1.855266 0.0636
## Xcb5v3.l1 -0.475463 1.1974424 21112 -0.397065 0.6913
## Xcb5v3.l2 1.080989 1.1616697 21112 0.930547 0.3521
## Xcb5v3.l3 2.917503 1.1124234 21112 2.622655 0.0087
## Xcb5v3.l4 -7.782315 0.8356822 21112 -9.312529 0.0000
## Xcb5v3.l5 2.910286 1.0319802 21112 2.820099 0.0048
## Xcb6v1.l1 -0.053014 0.4578367 21112 -0.115792 0.9078
## Xcb6v1.l2 0.139500 0.4463850 21112 0.312511 0.7547
## Xcb6v1.l3 -0.330962 0.4260007 21112 -0.776904 0.4372
## Xcb6v1.l4 -0.010815 0.2860972 21112 -0.037802 0.9698
## Xcb6v1.l5 0.358085 0.3619518 21112 0.989318 0.3225
## Xcb6v2.l1 -0.067358 0.3337206 21112 -0.201840 0.8400
## Xcb6v2.l2 0.262183 0.3256508 21112 0.805106 0.4208
## Xcb6v2.l3 -0.310227 0.3084347 21112 -1.005810 0.3145
## Xcb6v2.l4 0.529682 0.2117236 21112 2.501762 0.0124
## Xcb6v2.l5 -0.668605 0.2841891 21112 -2.352675 0.0186
## Xcb6v3.l1 -0.463214 0.4340200 21112 -1.067263 0.2859
## Xcb6v3.l2 0.622814 0.4237996 21112 1.469596 0.1417
## Xcb6v3.l3 -0.693762 0.4008974 21112 -1.730522 0.0836
## Xcb6v3.l4 0.016487 0.2764178 21112 0.059645 0.9524
## Xcb6v3.l5 -0.010858 0.3645467 21112 -0.029786 0.9762
## Xs(NumDate)Fx1 17.181707 0.7366950 21112 23.322688 0.0000
## Correlation:
## X(Int) Xc11.1 Xc11.2 Xc11.3 Xc11.4 Xc11.5 Xc12.1 Xc12.2 Xc12.3
## Xcb1v1.l1 -0.061
## Xcb1v1.l2 -0.070 -0.582
## Xcb1v1.l3 -0.066 0.237 -0.578
## Xcb1v1.l4 -0.219 -0.198 -0.289 -0.046
## Xcb1v1.l5 -0.020 0.139 0.181 -0.379 -0.068
## Xcb1v2.l1 0.031 -0.301 0.194 -0.085 0.024 -0.010
## Xcb1v2.l2 0.026 0.145 -0.300 0.179 0.096 -0.084 -0.601
## Xcb1v2.l3 0.004 -0.031 0.149 -0.299 0.031 0.129 0.245 -0.591
## Xcb1v2.l4 0.136 0.062 0.073 -0.006 -0.330 -0.014 -0.227 -0.321 -0.107
## Xcb1v2.l5 -0.022 -0.058 -0.019 0.080 0.071 -0.340 0.077 0.179 -0.378
## Xcb1v3.l1 -0.061 0.722 -0.439 0.190 -0.104 0.059 -0.515 0.302 -0.129
## Xcb1v3.l2 -0.066 -0.406 0.729 -0.434 -0.187 0.145 0.326 -0.506 0.308
## Xcb1v3.l3 -0.053 0.178 -0.402 0.727 -0.054 -0.261 -0.178 0.307 -0.513
## Xcb1v3.l4 -0.193 -0.145 -0.194 0.003 0.742 -0.017 0.089 0.113 0.038
## Xcb1v3.l5 -0.015 0.071 0.133 -0.266 -0.038 0.745 0.012 -0.139 0.198
## Xcb2v1.l1 0.009 -0.441 0.240 -0.085 0.142 -0.034 -0.169 0.124 -0.114
## Xcb2v1.l2 0.007 0.275 -0.442 0.247 0.148 -0.064 0.125 -0.158 0.128
## Xcb2v1.l3 0.010 -0.110 0.256 -0.436 0.028 0.181 -0.076 0.136 -0.169
## Xcb2v1.l4 0.005 0.117 0.177 0.064 -0.446 -0.004 0.061 0.000 0.054
## Xcb2v1.l5 0.014 -0.077 -0.101 0.188 0.005 -0.405 -0.024 -0.022 0.083
## Xcb2v2.l1 -0.009 0.188 -0.153 0.091 -0.016 -0.034 -0.305 0.207 -0.073
## Xcb2v2.l2 -0.004 -0.062 0.173 -0.140 -0.064 0.090 0.150 -0.320 0.202
## Xcb2v2.l3 0.004 0.016 -0.068 0.183 -0.057 -0.105 -0.035 0.154 -0.319
## Xcb2v2.l4 -0.021 -0.103 -0.027 -0.004 0.195 0.016 0.102 0.117 0.018
## Xcb2v2.l5 0.001 0.060 -0.017 -0.015 -0.051 0.180 -0.071 -0.022 0.090
## Xcb2v3.l1 0.017 -0.334 0.205 -0.084 0.082 -0.004 -0.080 0.043 -0.061
## Xcb2v3.l2 0.015 0.193 -0.338 0.209 0.105 -0.062 0.083 -0.065 0.041
## Xcb2v3.l3 0.016 -0.088 0.182 -0.335 0.043 0.139 -0.050 0.093 -0.074
## Xcb2v3.l4 0.052 0.118 0.120 0.023 -0.344 -0.007 0.020 -0.006 0.066
## Xcb2v3.l5 0.028 -0.059 -0.064 0.122 0.002 -0.301 -0.023 -0.017 0.054
## Xcb4v1.l1 -0.018 -0.213 0.183 -0.130 0.043 0.022 -0.024 0.026 0.023
## Xcb4v1.l2 -0.003 0.123 -0.210 0.168 0.048 -0.090 -0.013 -0.048 0.030
## Xcb4v1.l3 0.005 -0.070 0.136 -0.206 0.040 0.104 0.014 -0.017 -0.042
## Xcb4v1.l4 -0.008 0.082 0.012 0.040 -0.162 0.022 0.021 0.042 -0.041
## Xcb4v1.l5 0.013 -0.031 -0.011 0.075 0.008 -0.128 0.016 0.005 -0.021
## Xcb4v2.l1 0.003 0.105 -0.067 0.040 -0.039 0.020 -0.133 0.074 -0.052
## Xcb4v2.l2 -0.004 -0.084 0.099 -0.072 -0.010 0.012 0.106 -0.128 0.078
## Xcb4v2.l3 -0.003 0.056 -0.079 0.100 -0.023 -0.042 -0.058 0.102 -0.132
## Xcb4v2.l4 -0.025 -0.026 -0.015 -0.025 0.066 -0.020 0.020 0.025 0.036
## Xcb4v2.l5 0.012 -0.001 0.026 -0.055 -0.005 0.066 0.003 -0.029 0.065
## Xcb4v3.l1 -0.025 -0.129 0.112 -0.082 0.031 0.012 -0.024 0.020 0.015
## Xcb4v3.l2 -0.010 0.078 -0.125 0.104 0.023 -0.058 -0.009 -0.049 0.027
## Xcb4v3.l3 0.008 -0.053 0.086 -0.123 0.029 0.069 0.022 -0.012 -0.047
## Xcb4v3.l4 -0.089 0.059 0.000 0.029 -0.093 0.011 0.010 0.044 -0.033
## Xcb4v3.l5 0.008 -0.020 -0.009 0.051 0.007 -0.063 0.015 0.010 -0.016
## Xcb5v1.l1 -0.014 -0.127 0.041 0.051 0.003 -0.079 -0.082 0.050 -0.003
## Xcb5v1.l2 -0.009 0.065 -0.146 0.049 0.093 0.032 0.038 -0.077 0.045
## Xcb5v1.l3 0.008 0.006 0.046 -0.136 -0.003 0.024 -0.004 0.036 -0.069
## Xcb5v1.l4 -0.138 -0.002 0.117 -0.041 -0.188 0.016 0.033 0.043 0.003
## Xcb5v1.l5 -0.011 -0.017 -0.031 0.075 0.006 -0.184 -0.033 -0.013 0.042
## Xcb5v2.l1 0.014 0.059 0.001 -0.065 0.007 0.063 -0.034 0.029 -0.012
## Xcb5v2.l2 0.008 -0.023 0.072 -0.005 -0.063 -0.040 0.025 -0.038 0.032
## Xcb5v2.l3 -0.008 -0.018 -0.002 0.064 -0.003 0.002 -0.021 0.022 -0.034
## Xcb5v2.l4 0.112 0.010 -0.090 0.037 0.126 -0.018 0.007 0.004 -0.015
## Xcb5v2.l5 -0.007 0.001 0.021 -0.043 0.008 0.116 0.004 0.003 0.029
## Xcb5v3.l1 -0.021 -0.077 0.014 0.061 -0.008 -0.065 -0.036 0.014 -0.003
## Xcb5v3.l2 -0.014 0.031 -0.095 0.019 0.078 0.036 0.024 -0.030 0.013
## Xcb5v3.l3 0.008 0.022 0.014 -0.084 -0.003 0.008 -0.007 0.024 -0.030
## Xcb5v3.l4 -0.165 -0.008 0.098 -0.035 -0.136 0.016 0.010 0.015 0.012
## Xcb5v3.l5 0.015 -0.011 -0.018 0.048 0.001 -0.135 -0.011 -0.020 0.019
## Xcb6v1.l1 -0.030 0.022 -0.034 0.009 0.032 -0.027 0.012 -0.027 0.019
## Xcb6v1.l2 -0.018 0.028 0.033 -0.027 -0.042 0.033 -0.011 0.007 -0.018
## Xcb6v1.l3 -0.016 -0.039 0.020 0.024 -0.002 -0.028 0.016 -0.014 0.001
## Xcb6v1.l4 -0.237 -0.022 -0.019 0.025 0.069 -0.001 -0.015 0.023 -0.001
## Xcb6v1.l5 -0.017 0.034 -0.032 0.020 0.006 0.037 0.014 -0.009 0.009
## Xcb6v2.l1 0.021 0.072 -0.022 0.009 -0.053 0.042 -0.122 0.104 -0.079
## Xcb6v2.l2 0.005 -0.090 0.064 -0.028 0.008 -0.028 0.075 -0.117 0.096
## Xcb6v2.l3 0.027 0.065 -0.075 0.067 -0.003 -0.008 -0.042 0.074 -0.116
## Xcb6v2.l4 0.021 0.007 -0.021 -0.026 0.033 -0.003 0.044 0.003 0.022
## Xcb6v2.l5 0.030 -0.024 0.051 -0.060 -0.002 0.040 -0.019 0.008 0.036
## Xcb6v3.l1 -0.050 -0.077 0.013 0.013 0.058 -0.055 -0.090 0.052 -0.023
## Xcb6v3.l2 -0.024 0.093 -0.083 0.013 0.024 0.040 0.056 -0.082 0.062
## Xcb6v3.l3 -0.029 -0.044 0.088 -0.077 -0.016 -0.010 -0.034 0.046 -0.081
## Xcb6v3.l4 -0.292 -0.020 0.053 0.041 -0.054 0.004 0.029 0.030 -0.006
## Xcb6v3.l5 -0.019 0.011 -0.045 0.062 0.030 -0.044 -0.007 -0.025 0.050
## Xs(NumDate)Fx1 0.006 0.005 -0.008 -0.006 0.015 0.005 0.000 0.014 0.001
## Xc12.4 Xc12.5 Xc13.1 Xc13.2 Xc13.3 Xc13.4 Xc13.5 Xc21.1 Xc21.2
## Xcb1v1.l1
## Xcb1v1.l2
## Xcb1v1.l3
## Xcb1v1.l4
## Xcb1v1.l5
## Xcb1v2.l1
## Xcb1v2.l2
## Xcb1v2.l3
## Xcb1v2.l4
## Xcb1v2.l5 -0.051
## Xcb1v3.l1 0.097 -0.043
## Xcb1v3.l2 0.095 -0.071 -0.602
## Xcb1v3.l3 0.057 0.161 0.289 -0.599
## Xcb1v3.l4 -0.519 0.048 -0.176 -0.227 -0.053
## Xcb1v3.l5 -0.003 -0.530 0.056 0.200 -0.368 -0.017
## Xcb2v1.l1 0.070 0.017 -0.265 0.145 -0.034 0.073 -0.023
## Xcb2v1.l2 0.017 -0.076 0.158 -0.271 0.151 0.101 -0.025 -0.620
## Xcb2v1.l3 0.034 0.114 -0.064 0.144 -0.262 0.010 0.116 0.297 -0.615
## Xcb2v1.l4 -0.095 -0.032 0.069 0.116 0.019 -0.280 -0.012 -0.319 -0.294
## Xcb2v1.l5 0.052 -0.118 -0.048 -0.073 0.114 -0.013 -0.252 0.119 0.184
## Xcb2v2.l1 0.046 -0.004 0.139 -0.099 0.055 -0.023 -0.022 -0.281 0.201
## Xcb2v2.l2 0.137 -0.076 -0.064 0.130 -0.093 -0.035 0.049 0.159 -0.260
## Xcb2v2.l3 0.038 0.151 0.033 -0.068 0.142 -0.035 -0.068 -0.091 0.160
## Xcb2v2.l4 -0.331 0.022 -0.060 -0.018 -0.015 0.156 0.011 0.119 0.030
## Xcb2v2.l5 0.012 -0.344 0.016 0.014 -0.033 -0.015 0.136 -0.035 -0.031
## Xcb2v3.l1 0.072 -0.004 -0.322 0.220 -0.081 0.041 0.003 0.753 -0.481
## Xcb2v3.l2 -0.002 -0.033 0.157 -0.335 0.231 0.114 -0.069 -0.469 0.747
## Xcb2v3.l3 -0.001 0.054 -0.062 0.153 -0.332 0.045 0.171 0.239 -0.474
## Xcb2v3.l4 -0.022 -0.013 0.125 0.109 -0.018 -0.348 -0.022 -0.268 -0.191
## Xcb2v3.l5 0.057 -0.037 -0.078 -0.045 0.113 -0.029 -0.323 0.114 0.130
## Xcb4v1.l1 -0.041 0.007 -0.147 0.110 -0.088 0.055 0.009 -0.454 0.225
## Xcb4v1.l2 0.048 0.010 0.107 -0.144 0.096 0.029 -0.061 0.320 -0.458
## Xcb4v1.l3 0.028 -0.019 -0.051 0.111 -0.136 0.001 0.068 -0.153 0.315
## Xcb4v1.l4 -0.068 0.002 0.028 0.011 0.048 -0.095 0.018 0.087 0.168
## Xcb4v1.l5 -0.027 -0.054 -0.021 -0.010 0.055 0.014 -0.092 -0.007 -0.136
## Xcb4v2.l1 0.054 -0.016 0.033 -0.010 0.015 -0.030 0.015 0.200 -0.127
## Xcb4v2.l2 0.013 -0.026 -0.039 0.028 -0.012 0.006 0.007 -0.128 0.194
## Xcb4v2.l3 0.016 0.043 0.019 -0.036 0.034 -0.004 -0.004 0.061 -0.125
## Xcb4v2.l4 -0.107 0.015 0.005 -0.003 -0.022 0.010 -0.012 -0.060 -0.046
## Xcb4v2.l5 0.011 -0.109 -0.004 0.005 -0.023 -0.012 0.022 0.015 0.040
## Xcb4v3.l1 -0.032 0.014 -0.084 0.049 -0.051 0.055 -0.001 -0.325 0.166
## Xcb4v3.l2 0.047 0.001 0.082 -0.073 0.041 -0.008 -0.027 0.226 -0.327
## Xcb4v3.l3 0.023 -0.008 -0.056 0.080 -0.071 0.002 0.027 -0.105 0.220
## Xcb4v3.l4 -0.072 -0.008 0.008 -0.007 0.049 -0.047 0.009 0.067 0.111
## Xcb4v3.l5 -0.024 -0.058 0.009 -0.024 0.040 0.021 -0.032 -0.014 -0.091
## Xcb5v1.l1 0.022 -0.029 -0.055 0.037 0.003 -0.020 -0.020 0.053 -0.051
## Xcb5v1.l2 0.041 -0.005 0.012 -0.056 0.040 0.029 0.000 -0.035 0.057
## Xcb5v1.l3 0.009 0.041 -0.005 0.008 -0.061 0.018 0.019 0.040 -0.040
## Xcb5v1.l4 -0.075 0.017 0.026 0.024 -0.031 -0.082 0.006 -0.037 -0.003
## Xcb5v1.l5 0.011 -0.064 -0.007 0.000 0.021 -0.011 -0.086 -0.032 0.028
## Xcb5v2.l1 -0.009 -0.009 0.041 -0.027 -0.009 0.018 0.024 -0.038 0.015
## Xcb5v2.l2 0.005 -0.002 -0.002 0.042 -0.032 -0.027 -0.001 0.040 -0.033
## Xcb5v2.l3 0.013 0.015 -0.010 0.007 0.037 -0.020 -0.023 -0.047 0.035
## Xcb5v2.l4 -0.028 0.000 -0.019 -0.030 0.039 0.071 -0.009 0.019 0.008
## Xcb5v2.l5 -0.030 -0.006 0.015 -0.007 -0.019 0.008 0.064 0.039 -0.044
## Xcb5v3.l1 0.026 -0.016 -0.050 0.043 0.006 -0.029 -0.025 0.035 -0.019
## Xcb5v3.l2 0.010 -0.005 0.003 -0.056 0.051 0.032 -0.002 -0.030 0.040
## Xcb5v3.l3 -0.002 0.016 0.006 -0.002 -0.052 0.025 0.033 0.030 -0.030
## Xcb5v3.l4 -0.031 0.014 0.029 0.032 -0.050 -0.060 0.008 -0.015 -0.018
## Xcb5v3.l5 0.021 -0.034 -0.016 0.005 0.027 -0.016 -0.083 -0.028 0.035
## Xcb6v1.l1 0.006 0.005 -0.016 0.003 0.000 0.021 -0.029 0.085 -0.050
## Xcb6v1.l2 0.008 0.003 0.037 -0.014 0.009 -0.013 0.019 -0.088 0.086
## Xcb6v1.l3 0.001 -0.008 -0.031 0.033 -0.014 -0.003 -0.002 0.082 -0.086
## Xcb6v1.l4 -0.043 -0.014 -0.005 -0.001 0.005 0.029 -0.004 -0.025 -0.009
## Xcb6v1.l5 -0.007 -0.005 0.013 -0.034 0.038 0.019 -0.010 -0.038 0.060
## Xcb6v2.l1 0.025 0.020 0.060 -0.034 0.009 -0.023 0.035 -0.043 0.017
## Xcb6v2.l2 0.019 -0.048 -0.050 0.061 -0.044 -0.012 -0.006 0.053 -0.041
## Xcb6v2.l3 0.024 0.067 0.030 -0.044 0.064 -0.017 -0.033 -0.056 0.053
## Xcb6v2.l4 -0.086 0.001 -0.014 -0.024 0.006 0.051 0.004 0.009 0.003
## Xcb6v2.l5 -0.017 -0.066 0.000 0.023 -0.047 -0.004 0.047 0.026 -0.048
## Xcb6v3.l1 0.028 -0.019 -0.049 0.019 0.002 0.029 -0.029 0.089 -0.074
## Xcb6v3.l2 0.009 -0.006 0.056 -0.051 0.013 0.014 0.020 -0.061 0.093
## Xcb6v3.l3 0.028 0.043 -0.030 0.057 -0.050 -0.009 -0.011 0.035 -0.064
## Xcb6v3.l4 -0.088 -0.001 -0.004 0.022 0.026 -0.022 0.004 -0.029 -0.015
## Xcb6v3.l5 -0.003 -0.063 0.006 -0.026 0.038 0.024 -0.033 0.023 0.011
## Xs(NumDate)Fx1 -0.012 -0.006 0.020 0.001 -0.005 0.015 -0.006 -0.010 -0.001
## Xc21.3 Xc21.4 Xc21.5 Xc22.1 Xc22.2 Xc22.3 Xc22.4 Xc22.5 Xc23.1
## Xcb1v1.l1
## Xcb1v1.l2
## Xcb1v1.l3
## Xcb1v1.l4
## Xcb1v1.l5
## Xcb1v2.l1
## Xcb1v2.l2
## Xcb1v2.l3
## Xcb1v2.l4
## Xcb1v2.l5
## Xcb1v3.l1
## Xcb1v3.l2
## Xcb1v3.l3
## Xcb1v3.l4
## Xcb1v3.l5
## Xcb2v1.l1
## Xcb2v1.l2
## Xcb2v1.l3
## Xcb2v1.l4 -0.161
## Xcb2v1.l5 -0.426 -0.034
## Xcb2v2.l1 -0.137 0.083 0.004
## Xcb2v2.l2 0.191 0.048 -0.098 -0.616
## Xcb2v2.l3 -0.270 0.081 0.118 0.266 -0.609
## Xcb2v2.l4 0.030 -0.256 -0.030 -0.286 -0.317 -0.116
## Xcb2v2.l5 0.068 0.076 -0.260 0.144 0.163 -0.390 -0.099
## Xcb2v3.l1 0.256 -0.243 0.066 -0.461 0.301 -0.186 0.160 -0.034
## Xcb2v3.l2 -0.473 -0.199 0.166 0.305 -0.433 0.302 0.062 -0.100 -0.643
## Xcb2v3.l3 0.753 -0.124 -0.329 -0.193 0.293 -0.435 0.077 0.171 0.340
## Xcb2v3.l4 -0.128 0.757 -0.037 0.166 0.067 0.085 -0.416 0.076 -0.337
## Xcb2v3.l5 -0.302 -0.063 0.743 -0.048 -0.110 0.177 0.039 -0.401 0.142
## Xcb4v1.l1 -0.088 0.189 -0.073 0.123 -0.083 0.051 -0.038 0.014 -0.295
## Xcb4v1.l2 0.226 0.133 -0.024 -0.090 0.123 -0.089 -0.005 0.022 0.204
## Xcb4v1.l3 -0.475 0.043 0.148 0.070 -0.090 0.136 -0.019 -0.036 -0.119
## Xcb4v1.l4 0.114 -0.478 -0.014 -0.039 -0.014 -0.023 0.093 -0.021 0.074
## Xcb4v1.l5 0.216 0.011 -0.494 -0.014 0.044 -0.055 0.031 0.136 0.013
## Xcb4v2.l1 0.078 -0.071 0.011 -0.482 0.273 -0.110 0.153 -0.076 0.271
## Xcb4v2.l2 -0.119 -0.044 0.047 0.316 -0.490 0.277 0.147 -0.053 -0.179
## Xcb4v2.l3 0.194 -0.033 -0.071 -0.148 0.306 -0.490 0.058 0.155 0.118
## Xcb4v2.l4 -0.031 0.192 0.026 0.116 0.172 0.074 -0.519 0.028 -0.098
## Xcb4v2.l5 -0.061 -0.037 0.194 -0.022 -0.109 0.194 0.030 -0.501 0.006
## Xcb4v3.l1 -0.074 0.134 -0.038 0.239 -0.137 0.077 -0.093 0.030 -0.472
## Xcb4v3.l2 0.164 0.091 -0.028 -0.171 0.233 -0.141 -0.038 0.033 0.310
## Xcb4v3.l3 -0.334 0.031 0.101 0.101 -0.159 0.235 -0.036 -0.070 -0.166
## Xcb4v3.l4 0.083 -0.347 0.001 -0.058 -0.057 -0.044 0.218 -0.014 0.131
## Xcb4v3.l5 0.131 0.026 -0.355 -0.002 0.074 -0.097 -0.011 0.229 -0.023
## Xcb5v1.l1 0.030 -0.010 0.010 -0.042 0.046 -0.058 0.029 -0.005 0.051
## Xcb5v1.l2 -0.054 -0.004 0.021 0.056 -0.034 0.051 -0.041 -0.011 -0.040
## Xcb5v1.l3 0.066 -0.037 -0.026 -0.071 0.056 -0.048 0.027 0.011 0.050
## Xcb5v1.l4 -0.002 0.024 -0.020 0.021 -0.033 0.013 0.034 -0.009 -0.037
## Xcb5v1.l5 -0.066 0.046 0.031 0.048 -0.035 0.049 -0.031 0.012 -0.029
## Xcb5v2.l1 0.001 0.022 -0.035 0.075 -0.082 0.092 -0.038 -0.011 -0.057
## Xcb5v2.l2 0.021 -0.010 0.015 -0.091 0.069 -0.101 0.066 0.019 0.068
## Xcb5v2.l3 -0.040 0.028 -0.002 0.127 -0.094 0.103 -0.072 -0.031 -0.080
## Xcb5v2.l4 -0.002 -0.017 0.014 -0.047 0.048 -0.019 -0.028 0.021 0.027
## Xcb5v2.l5 0.057 -0.029 -0.026 -0.077 0.058 -0.066 0.047 0.002 0.039
## Xcb5v3.l1 -0.007 -0.007 0.034 -0.060 0.058 -0.052 0.028 -0.012 0.073
## Xcb5v3.l2 -0.027 -0.005 -0.006 0.075 -0.059 0.075 -0.044 0.003 -0.090
## Xcb5v3.l3 0.043 -0.022 -0.010 -0.103 0.073 -0.083 0.063 0.013 0.101
## Xcb5v3.l4 0.012 0.018 -0.022 0.034 -0.023 0.003 0.021 -0.013 -0.026
## Xcb5v3.l5 -0.059 0.030 0.029 0.072 -0.055 0.062 -0.039 -0.007 -0.058
## Xcb6v1.l1 0.033 -0.037 0.032 -0.026 0.052 -0.024 -0.037 0.010 -0.061
## Xcb6v1.l2 -0.070 0.019 0.001 -0.010 -0.034 0.055 0.018 -0.029 0.002
## Xcb6v1.l3 0.103 -0.037 -0.036 0.009 -0.002 -0.046 0.016 0.041 0.023
## Xcb6v1.l4 -0.007 0.037 0.010 0.033 -0.002 -0.025 -0.015 0.019 0.029
## Xcb6v1.l5 -0.077 0.006 0.095 -0.029 0.031 -0.018 -0.009 -0.082 -0.041
## Xcb6v2.l1 -0.007 0.023 -0.022 -0.034 -0.020 0.052 0.022 -0.071 -0.054
## Xcb6v2.l2 0.028 -0.014 0.007 0.049 -0.047 -0.003 0.015 0.048 0.047
## Xcb6v2.l3 -0.050 0.014 0.014 -0.033 0.041 -0.041 0.008 -0.030 -0.048
## Xcb6v2.l4 0.006 -0.013 0.008 -0.017 0.057 -0.016 -0.058 0.020 0.024
## Xcb6v2.l5 0.052 -0.007 -0.040 0.044 -0.056 0.051 0.012 -0.073 0.007
## Xcb6v3.l1 0.046 -0.016 0.005 0.015 0.008 0.007 -0.032 0.002 -0.027
## Xcb6v3.l2 -0.075 -0.013 0.032 -0.023 0.005 0.006 0.012 -0.003 0.004
## Xcb6v3.l3 0.091 -0.013 -0.060 0.016 -0.019 0.000 0.007 0.002 0.001
## Xcb6v3.l4 -0.003 0.052 -0.001 0.009 -0.002 -0.017 0.012 0.012 0.012
## Xcb6v3.l5 -0.038 -0.014 0.078 -0.002 0.021 -0.018 -0.013 -0.020 0.000
## Xs(NumDate)Fx1 0.009 -0.012 0.012 0.011 -0.004 -0.011 0.028 -0.013 -0.021
## Xc23.2 Xc23.3 Xc23.4 Xc23.5 Xc41.1 Xc41.2 Xc41.3 Xc41.4 Xc41.5
## Xcb1v1.l1
## Xcb1v1.l2
## Xcb1v1.l3
## Xcb1v1.l4
## Xcb1v1.l5
## Xcb1v2.l1
## Xcb1v2.l2
## Xcb1v2.l3
## Xcb1v2.l4
## Xcb1v2.l5
## Xcb1v3.l1
## Xcb1v3.l2
## Xcb1v3.l3
## Xcb1v3.l4
## Xcb1v3.l5
## Xcb2v1.l1
## Xcb2v1.l2
## Xcb2v1.l3
## Xcb2v1.l4
## Xcb2v1.l5
## Xcb2v2.l1
## Xcb2v2.l2
## Xcb2v2.l3
## Xcb2v2.l4
## Xcb2v2.l5
## Xcb2v3.l1
## Xcb2v3.l2
## Xcb2v3.l3 -0.647
## Xcb2v3.l4 -0.243 -0.147
## Xcb2v3.l5 0.203 -0.428 -0.098
## Xcb4v1.l1 0.157 -0.068 0.122 -0.051
## Xcb4v1.l2 -0.293 0.165 0.065 -0.017 -0.601
## Xcb4v1.l3 0.199 -0.315 0.055 0.098 0.252 -0.585
## Xcb4v1.l4 0.092 0.065 -0.324 0.000 -0.277 -0.345 -0.141
## Xcb4v1.l5 -0.103 0.144 -0.001 -0.316 0.101 0.171 -0.389 -0.034
## Xcb4v2.l1 -0.174 0.095 -0.094 0.044 -0.381 0.263 -0.154 0.105 -0.010
## Xcb4v2.l2 0.257 -0.171 -0.041 0.045 0.231 -0.364 0.258 0.080 -0.096
## Xcb4v2.l3 -0.171 0.255 -0.065 -0.102 -0.132 0.222 -0.374 0.089 0.162
## Xcb4v2.l4 -0.055 -0.035 0.269 -0.013 0.144 0.072 0.044 -0.337 -0.014
## Xcb4v2.l5 0.080 -0.108 -0.031 0.227 -0.041 -0.068 0.117 0.050 -0.367
## Xcb4v3.l1 0.279 -0.126 0.164 -0.076 0.729 -0.444 0.209 -0.206 0.046
## Xcb4v3.l2 -0.475 0.282 0.117 -0.061 -0.447 0.731 -0.435 -0.231 0.143
## Xcb4v3.l3 0.298 -0.478 0.083 0.162 0.200 -0.435 0.735 -0.111 -0.280
## Xcb4v3.l4 0.140 0.069 -0.503 0.031 -0.214 -0.224 -0.118 0.744 -0.019
## Xcb4v3.l5 -0.129 0.193 0.039 -0.495 0.073 0.125 -0.258 -0.047 0.737
## Xcb5v1.l1 -0.057 0.051 -0.020 -0.003 -0.143 0.071 -0.030 0.057 -0.042
## Xcb5v1.l2 0.048 -0.062 0.021 0.025 0.108 -0.140 0.067 0.035 -0.018
## Xcb5v1.l3 -0.044 0.064 -0.037 -0.035 -0.052 0.109 -0.142 0.001 0.038
## Xcb5v1.l4 0.018 -0.006 0.003 -0.002 0.021 0.043 0.032 -0.141 0.033
## Xcb5v1.l5 0.026 -0.055 0.045 0.019 -0.003 -0.038 0.094 -0.019 -0.109
## Xcb5v2.l1 0.043 -0.041 0.042 -0.031 0.119 -0.072 0.038 -0.041 0.014
## Xcb5v2.l2 -0.044 0.056 -0.050 0.004 -0.092 0.120 -0.070 -0.023 0.024
## Xcb5v2.l3 0.065 -0.057 0.034 0.023 0.051 -0.092 0.122 -0.007 -0.044
## Xcb5v2.l4 -0.024 0.001 0.023 -0.002 -0.023 -0.028 -0.024 0.104 -0.010
## Xcb5v2.l5 -0.054 0.069 -0.029 -0.004 0.003 0.032 -0.069 0.008 0.082
## Xcb5v3.l1 -0.053 0.036 -0.044 0.045 -0.112 0.065 -0.030 0.037 -0.021
## Xcb5v3.l2 0.074 -0.079 0.048 -0.006 0.082 -0.113 0.063 0.026 -0.022
## Xcb5v3.l3 -0.090 0.090 -0.047 -0.035 -0.039 0.082 -0.115 0.005 0.042
## Xcb5v3.l4 0.009 0.012 -0.022 0.000 0.018 0.031 0.019 -0.106 0.020
## Xcb5v3.l5 0.077 -0.102 0.039 0.027 -0.010 -0.025 0.065 -0.010 -0.083
## Xcb6v1.l1 0.049 -0.024 0.006 0.021 -0.092 0.070 -0.047 0.027 -0.018
## Xcb6v1.l2 -0.054 0.022 0.050 -0.031 0.083 -0.092 0.083 -0.014 -0.014
## Xcb6v1.l3 0.004 -0.037 -0.003 0.011 -0.082 0.082 -0.104 0.045 0.047
## Xcb6v1.l4 0.022 0.018 -0.115 0.021 0.040 0.002 0.000 -0.036 -0.009
## Xcb6v1.l5 0.040 -0.028 0.005 -0.028 0.031 -0.045 0.061 -0.005 -0.084
## Xcb6v2.l1 0.044 -0.032 0.018 -0.015 -0.001 0.010 0.011 -0.023 -0.010
## Xcb6v2.l2 -0.050 0.052 -0.015 -0.012 -0.037 0.001 0.005 0.030 -0.007
## Xcb6v2.l3 0.048 -0.058 0.020 0.032 0.047 -0.037 0.010 -0.005 0.022
## Xcb6v2.l4 -0.009 -0.002 -0.013 0.005 0.013 0.014 -0.028 -0.028 0.008
## Xcb6v2.l5 -0.027 0.040 -0.008 -0.031 -0.045 0.031 -0.023 0.012 -0.032
## Xcb6v3.l1 0.009 -0.003 0.011 -0.002 -0.004 -0.002 -0.022 0.031 -0.016
## Xcb6v3.l2 -0.016 -0.002 0.017 -0.001 0.051 0.002 0.009 -0.056 0.008
## Xcb6v3.l3 0.003 -0.012 0.003 -0.015 -0.071 0.045 -0.007 0.021 -0.009
## Xcb6v3.l4 0.007 0.012 -0.063 0.012 -0.004 -0.029 0.010 0.059 -0.003
## Xcb6v3.l5 0.007 -0.010 -0.005 -0.003 0.047 -0.039 0.039 -0.011 0.038
## Xs(NumDate)Fx1 -0.007 0.016 -0.013 0.037 0.000 0.007 0.000 -0.011 -0.007
## Xc42.1 Xc42.2 Xc42.3 Xc42.4 Xc42.5 Xc43.1 Xc43.2 Xc43.3 Xc43.4
## Xcb1v1.l1
## Xcb1v1.l2
## Xcb1v1.l3
## Xcb1v1.l4
## Xcb1v1.l5
## Xcb1v2.l1
## Xcb1v2.l2
## Xcb1v2.l3
## Xcb1v2.l4
## Xcb1v2.l5
## Xcb1v3.l1
## Xcb1v3.l2
## Xcb1v3.l3
## Xcb1v3.l4
## Xcb1v3.l5
## Xcb2v1.l1
## Xcb2v1.l2
## Xcb2v1.l3
## Xcb2v1.l4
## Xcb2v1.l5
## Xcb2v2.l1
## Xcb2v2.l2
## Xcb2v2.l3
## Xcb2v2.l4
## Xcb2v2.l5
## Xcb2v3.l1
## Xcb2v3.l2
## Xcb2v3.l3
## Xcb2v3.l4
## Xcb2v3.l5
## Xcb4v1.l1
## Xcb4v1.l2
## Xcb4v1.l3
## Xcb4v1.l4
## Xcb4v1.l5
## Xcb4v2.l1
## Xcb4v2.l2 -0.638
## Xcb4v2.l3 0.325 -0.632
## Xcb4v2.l4 -0.285 -0.263 -0.149
## Xcb4v2.l5 0.081 0.193 -0.376 -0.069
## Xcb4v3.l1 -0.571 0.364 -0.221 0.204 -0.033
## Xcb4v3.l2 0.384 -0.547 0.349 0.109 -0.121 -0.634
## Xcb4v3.l3 -0.221 0.373 -0.547 0.082 0.199 0.330 -0.620
## Xcb4v3.l4 0.173 0.104 0.128 -0.523 0.049 -0.298 -0.253 -0.168
## Xcb4v3.l5 -0.044 -0.125 0.212 0.035 -0.515 0.069 0.201 -0.360 -0.067
## Xcb5v1.l1 0.086 -0.061 0.031 -0.023 0.008 -0.118 0.067 -0.031 0.039
## Xcb5v1.l2 -0.060 0.095 -0.061 -0.025 0.020 0.088 -0.126 0.067 0.029
## Xcb5v1.l3 0.036 -0.066 0.110 -0.015 -0.031 -0.045 0.090 -0.135 0.006
## Xcb5v1.l4 -0.028 -0.022 -0.018 0.087 -0.011 0.014 0.035 0.018 -0.125
## Xcb5v1.l5 -0.011 0.029 -0.055 0.024 0.095 0.004 -0.034 0.069 -0.015
## Xcb5v2.l1 -0.111 0.082 -0.055 0.040 -0.007 0.122 -0.071 0.037 -0.047
## Xcb5v2.l2 0.091 -0.113 0.090 -0.004 -0.018 -0.104 0.133 -0.078 -0.016
## Xcb5v2.l3 -0.086 0.092 -0.143 0.057 0.028 0.065 -0.104 0.145 -0.014
## Xcb5v2.l4 0.048 0.005 0.023 -0.083 0.005 -0.014 -0.032 -0.016 0.102
## Xcb5v2.l5 0.045 -0.048 0.063 -0.026 -0.103 -0.016 0.050 -0.073 0.004
## Xcb5v3.l1 0.142 -0.103 0.056 -0.045 0.016 -0.128 0.071 -0.030 0.049
## Xcb5v3.l2 -0.107 0.151 -0.112 -0.019 0.027 0.113 -0.143 0.086 0.013
## Xcb5v3.l3 0.082 -0.110 0.176 -0.052 -0.055 -0.075 0.114 -0.161 0.023
## Xcb5v3.l4 -0.048 -0.026 -0.019 0.112 -0.012 0.012 0.039 0.009 -0.110
## Xcb5v3.l5 -0.026 0.053 -0.083 0.021 0.138 0.028 -0.064 0.091 -0.017
## Xcb6v1.l1 -0.047 -0.007 0.025 0.035 -0.022 0.186 -0.109 0.041 -0.052
## Xcb6v1.l2 0.028 -0.050 -0.016 0.055 0.003 -0.091 0.186 -0.086 -0.100
## Xcb6v1.l3 0.036 0.025 -0.025 -0.055 0.007 -0.005 -0.096 0.162 0.031
## Xcb6v1.l4 -0.032 0.063 0.018 -0.112 0.007 -0.034 -0.083 -0.044 0.249
## Xcb6v1.l5 -0.046 -0.008 0.030 0.028 -0.039 0.084 0.001 -0.052 -0.030
## Xcb6v2.l1 0.272 -0.159 0.046 -0.064 0.086 0.080 -0.050 0.044 -0.039
## Xcb6v2.l2 -0.176 0.277 -0.170 -0.085 0.036 -0.075 0.085 -0.052 -0.002
## Xcb6v2.l3 0.075 -0.175 0.263 -0.012 -0.124 0.049 -0.078 0.093 -0.013
## Xcb6v2.l4 -0.053 -0.105 0.012 0.255 -0.044 -0.011 -0.009 -0.031 0.054
## Xcb6v2.l5 0.041 0.059 -0.132 -0.024 0.293 -0.015 0.030 -0.045 -0.008
## Xcb6v3.l1 -0.009 -0.019 0.015 0.025 -0.011 0.167 -0.105 0.038 -0.035
## Xcb6v3.l2 0.009 -0.001 -0.027 0.015 0.010 -0.070 0.165 -0.086 -0.092
## Xcb6v3.l3 0.018 0.004 0.016 -0.035 -0.013 -0.009 -0.076 0.147 0.017
## Xcb6v3.l4 -0.024 0.022 0.012 -0.027 -0.001 -0.042 -0.072 -0.029 0.212
## Xcb6v3.l5 -0.019 -0.002 0.003 0.016 0.017 0.073 -0.006 -0.030 -0.029
## Xs(NumDate)Fx1 -0.001 -0.006 0.002 -0.008 -0.001 -0.001 0.006 0.001 -0.018
## Xc43.5 Xc51.1 Xc51.2 Xc51.3 Xc51.4 Xc51.5 Xc52.1 Xc52.2 Xc52.3
## Xcb1v1.l1
## Xcb1v1.l2
## Xcb1v1.l3
## Xcb1v1.l4
## Xcb1v1.l5
## Xcb1v2.l1
## Xcb1v2.l2
## Xcb1v2.l3
## Xcb1v2.l4
## Xcb1v2.l5
## Xcb1v3.l1
## Xcb1v3.l2
## Xcb1v3.l3
## Xcb1v3.l4
## Xcb1v3.l5
## Xcb2v1.l1
## Xcb2v1.l2
## Xcb2v1.l3
## Xcb2v1.l4
## Xcb2v1.l5
## Xcb2v2.l1
## Xcb2v2.l2
## Xcb2v2.l3
## Xcb2v2.l4
## Xcb2v2.l5
## Xcb2v3.l1
## Xcb2v3.l2
## Xcb2v3.l3
## Xcb2v3.l4
## Xcb2v3.l5
## Xcb4v1.l1
## Xcb4v1.l2
## Xcb4v1.l3
## Xcb4v1.l4
## Xcb4v1.l5
## Xcb4v2.l1
## Xcb4v2.l2
## Xcb4v2.l3
## Xcb4v2.l4
## Xcb4v2.l5
## Xcb4v3.l1
## Xcb4v3.l2
## Xcb4v3.l3
## Xcb4v3.l4
## Xcb4v3.l5
## Xcb5v1.l1 -0.026
## Xcb5v1.l2 -0.018 -0.599
## Xcb5v1.l3 0.031 0.217 -0.594
## Xcb5v1.l4 0.018 -0.217 -0.352 -0.060
## Xcb5v1.l5 -0.128 0.187 0.162 -0.438 -0.089
## Xcb5v2.l1 0.023 -0.681 0.421 -0.135 0.112 -0.133
## Xcb5v2.l2 0.013 0.378 -0.693 0.420 0.265 -0.115 -0.598
## Xcb5v2.l3 -0.034 -0.116 0.357 -0.689 0.081 0.291 0.208 -0.585
## Xcb5v2.l4 -0.001 0.167 0.256 0.015 -0.744 0.086 -0.230 -0.338 -0.088
## Xcb5v2.l5 0.125 -0.136 -0.078 0.253 0.076 -0.728 0.199 0.145 -0.422
## Xcb5v3.l1 -0.038 0.793 -0.493 0.181 -0.150 0.140 -0.895 0.532 -0.188
## Xcb5v3.l2 -0.011 -0.453 0.797 -0.488 -0.287 0.144 0.536 -0.897 0.514
## Xcb5v3.l3 0.037 0.151 -0.436 0.794 -0.081 -0.353 -0.179 0.529 -0.894
## Xcb5v3.l4 0.014 -0.187 -0.268 -0.040 0.815 -0.093 0.186 0.297 0.092
## Xcb5v3.l5 -0.148 0.158 0.097 -0.304 -0.093 0.803 -0.184 -0.131 0.371
## Xcb6v1.l1 -0.002 0.043 -0.012 0.005 -0.029 0.033 -0.220 0.119 -0.047
## Xcb6v1.l2 0.047 -0.038 0.025 -0.010 0.008 -0.016 0.171 -0.202 0.126
## Xcb6v1.l3 -0.086 0.025 -0.027 0.023 -0.012 0.002 -0.106 0.158 -0.200
## Xcb6v1.l4 -0.013 -0.004 -0.003 -0.009 0.053 -0.009 0.049 0.052 0.017
## Xcb6v1.l5 0.156 0.003 0.014 -0.022 -0.038 0.043 0.001 -0.078 0.143
## Xcb6v2.l1 -0.004 -0.222 0.111 -0.025 0.060 -0.086 0.390 -0.210 0.048
## Xcb6v2.l2 0.019 0.163 -0.219 0.119 0.050 0.005 -0.262 0.391 -0.215
## Xcb6v2.l3 -0.026 -0.083 0.145 -0.211 0.015 0.051 0.122 -0.251 0.386
## Xcb6v2.l4 0.010 0.033 0.087 0.006 -0.219 0.039 -0.086 -0.157 -0.019
## Xcb6v2.l5 0.052 -0.006 -0.070 0.104 0.029 -0.231 0.030 0.103 -0.193
## Xcb6v3.l1 -0.006 0.383 -0.166 0.041 -0.155 0.148 -0.179 0.064 -0.018
## Xcb6v3.l2 0.042 -0.304 0.374 -0.180 -0.094 0.002 0.160 -0.171 0.065
## Xcb6v3.l3 -0.084 0.145 -0.296 0.369 0.007 -0.134 -0.078 0.153 -0.161
## Xcb6v3.l4 -0.008 -0.033 -0.150 -0.048 0.362 -0.041 -0.006 0.065 0.041
## Xcb6v3.l5 0.148 0.021 0.141 -0.250 -0.071 0.367 -0.010 -0.074 0.115
## Xs(NumDate)Fx1 -0.005 -0.007 -0.008 0.001 0.000 0.015 0.010 -0.001 -0.016
## Xc52.4 Xc52.5 Xc53.1 Xc53.2 Xc53.3 Xc53.4 Xc53.5 Xc61.1 Xc61.2
## Xcb1v1.l1
## Xcb1v1.l2
## Xcb1v1.l3
## Xcb1v1.l4
## Xcb1v1.l5
## Xcb1v2.l1
## Xcb1v2.l2
## Xcb1v2.l3
## Xcb1v2.l4
## Xcb1v2.l5
## Xcb1v3.l1
## Xcb1v3.l2
## Xcb1v3.l3
## Xcb1v3.l4
## Xcb1v3.l5
## Xcb2v1.l1
## Xcb2v1.l2
## Xcb2v1.l3
## Xcb2v1.l4
## Xcb2v1.l5
## Xcb2v2.l1
## Xcb2v2.l2
## Xcb2v2.l3
## Xcb2v2.l4
## Xcb2v2.l5
## Xcb2v3.l1
## Xcb2v3.l2
## Xcb2v3.l3
## Xcb2v3.l4
## Xcb2v3.l5
## Xcb4v1.l1
## Xcb4v1.l2
## Xcb4v1.l3
## Xcb4v1.l4
## Xcb4v1.l5
## Xcb4v2.l1
## Xcb4v2.l2
## Xcb4v2.l3
## Xcb4v2.l4
## Xcb4v2.l5
## Xcb4v3.l1
## Xcb4v3.l2
## Xcb4v3.l3
## Xcb4v3.l4
## Xcb4v3.l5
## Xcb5v1.l1
## Xcb5v1.l2
## Xcb5v1.l3
## Xcb5v1.l4
## Xcb5v1.l5
## Xcb5v2.l1
## Xcb5v2.l2
## Xcb5v2.l3
## Xcb5v2.l4
## Xcb5v2.l5 -0.114
## Xcb5v3.l1 0.207 -0.171
## Xcb5v3.l2 0.309 -0.135 -0.594
## Xcb5v3.l3 0.064 0.380 0.204 -0.584
## Xcb5v3.l4 -0.908 0.112 -0.215 -0.336 -0.085
## Xcb5v3.l5 0.125 -0.896 0.198 0.155 -0.427 -0.136
## Xcb6v1.l1 0.079 -0.081 0.212 -0.105 0.033 -0.073 0.082
## Xcb6v1.l2 0.023 -0.002 -0.158 0.190 -0.106 -0.029 -0.008 -0.637
## Xcb6v1.l3 0.038 0.075 0.092 -0.138 0.183 -0.034 -0.054 0.284 -0.651
## Xcb6v1.l4 -0.191 0.028 -0.043 -0.061 -0.019 0.201 -0.038 -0.264 -0.309
## Xcb6v1.l5 0.032 -0.177 0.005 0.066 -0.114 -0.042 0.186 0.165 0.203
## Xcb6v2.l1 -0.100 0.125 -0.164 0.086 -0.013 0.032 -0.064 -0.106 0.046
## Xcb6v2.l2 -0.117 0.015 0.113 -0.166 0.090 0.048 -0.002 0.052 -0.119
## Xcb6v2.l3 -0.035 -0.126 -0.049 0.103 -0.155 0.005 0.049 0.013 0.058
## Xcb6v2.l4 0.374 -0.064 0.024 0.069 -0.004 -0.165 0.041 0.003 0.082
## Xcb6v2.l5 -0.029 0.392 -0.014 -0.049 0.080 0.030 -0.167 -0.056 -0.022
## Xcb6v3.l1 0.090 -0.082 0.317 -0.133 0.031 -0.126 0.123 0.731 -0.450
## Xcb6v3.l2 0.034 0.016 -0.252 0.308 -0.137 -0.075 -0.009 -0.476 0.721
## Xcb6v3.l3 -0.017 0.043 0.121 -0.240 0.298 0.002 -0.086 0.223 -0.481
## Xcb6v3.l4 -0.183 0.027 -0.024 -0.124 -0.049 0.312 -0.049 -0.178 -0.220
## Xcb6v3.l5 0.057 -0.158 0.017 0.111 -0.187 -0.065 0.298 0.103 0.148
## Xs(NumDate)Fx1 0.037 -0.032 -0.015 0.002 0.016 -0.029 0.035 -0.001 0.006
## Xc61.3 Xc61.4 Xc61.5 Xc62.1 Xc62.2 Xc62.3 Xc62.4 Xc62.5 Xc63.1
## Xcb1v1.l1
## Xcb1v1.l2
## Xcb1v1.l3
## Xcb1v1.l4
## Xcb1v1.l5
## Xcb1v2.l1
## Xcb1v2.l2
## Xcb1v2.l3
## Xcb1v2.l4
## Xcb1v2.l5
## Xcb1v3.l1
## Xcb1v3.l2
## Xcb1v3.l3
## Xcb1v3.l4
## Xcb1v3.l5
## Xcb2v1.l1
## Xcb2v1.l2
## Xcb2v1.l3
## Xcb2v1.l4
## Xcb2v1.l5
## Xcb2v2.l1
## Xcb2v2.l2
## Xcb2v2.l3
## Xcb2v2.l4
## Xcb2v2.l5
## Xcb2v3.l1
## Xcb2v3.l2
## Xcb2v3.l3
## Xcb2v3.l4
## Xcb2v3.l5
## Xcb4v1.l1
## Xcb4v1.l2
## Xcb4v1.l3
## Xcb4v1.l4
## Xcb4v1.l5
## Xcb4v2.l1
## Xcb4v2.l2
## Xcb4v2.l3
## Xcb4v2.l4
## Xcb4v2.l5
## Xcb4v3.l1
## Xcb4v3.l2
## Xcb4v3.l3
## Xcb4v3.l4
## Xcb4v3.l5
## Xcb5v1.l1
## Xcb5v1.l2
## Xcb5v1.l3
## Xcb5v1.l4
## Xcb5v1.l5
## Xcb5v2.l1
## Xcb5v2.l2
## Xcb5v2.l3
## Xcb5v2.l4
## Xcb5v2.l5
## Xcb5v3.l1
## Xcb5v3.l2
## Xcb5v3.l3
## Xcb5v3.l4
## Xcb5v3.l5
## Xcb6v1.l1
## Xcb6v1.l2
## Xcb6v1.l3
## Xcb6v1.l4 -0.077
## Xcb6v1.l5 -0.514 -0.064
## Xcb6v2.l1 0.032 -0.004 -0.052
## Xcb6v2.l2 0.046 0.085 -0.011 -0.591
## Xcb6v2.l3 -0.103 -0.048 0.073 0.191 -0.602
## Xcb6v2.l4 -0.026 -0.164 0.038 -0.246 -0.389 -0.039
## Xcb6v2.l5 0.058 0.009 -0.180 0.223 0.161 -0.456 -0.104
## Xcb6v3.l1 0.195 -0.201 0.147 -0.156 0.072 0.006 0.026 -0.083
## Xcb6v3.l2 -0.461 -0.208 0.126 0.097 -0.161 0.074 0.077 -0.015 -0.605
## Xcb6v3.l3 0.724 -0.055 -0.345 -0.003 0.095 -0.137 -0.045 0.064 0.217
## Xcb6v3.l4 -0.051 0.710 -0.073 -0.007 0.086 -0.040 -0.187 0.032 -0.233
## Xcb6v3.l5 -0.350 -0.047 0.706 -0.055 -0.033 0.093 0.043 -0.203 0.207
## Xs(NumDate)Fx1 0.005 -0.025 0.006 0.001 -0.005 -0.005 0.015 -0.010 -0.003
## Xc63.2 Xc63.3 Xc63.4 Xc63.5
## Xcb1v1.l1
## Xcb1v1.l2
## Xcb1v1.l3
## Xcb1v1.l4
## Xcb1v1.l5
## Xcb1v2.l1
## Xcb1v2.l2
## Xcb1v2.l3
## Xcb1v2.l4
## Xcb1v2.l5
## Xcb1v3.l1
## Xcb1v3.l2
## Xcb1v3.l3
## Xcb1v3.l4
## Xcb1v3.l5
## Xcb2v1.l1
## Xcb2v1.l2
## Xcb2v1.l3
## Xcb2v1.l4
## Xcb2v1.l5
## Xcb2v2.l1
## Xcb2v2.l2
## Xcb2v2.l3
## Xcb2v2.l4
## Xcb2v2.l5
## Xcb2v3.l1
## Xcb2v3.l2
## Xcb2v3.l3
## Xcb2v3.l4
## Xcb2v3.l5
## Xcb4v1.l1
## Xcb4v1.l2
## Xcb4v1.l3
## Xcb4v1.l4
## Xcb4v1.l5
## Xcb4v2.l1
## Xcb4v2.l2
## Xcb4v2.l3
## Xcb4v2.l4
## Xcb4v2.l5
## Xcb4v3.l1
## Xcb4v3.l2
## Xcb4v3.l3
## Xcb4v3.l4
## Xcb4v3.l5
## Xcb5v1.l1
## Xcb5v1.l2
## Xcb5v1.l3
## Xcb5v1.l4
## Xcb5v1.l5
## Xcb5v2.l1
## Xcb5v2.l2
## Xcb5v2.l3
## Xcb5v2.l4
## Xcb5v2.l5
## Xcb5v3.l1
## Xcb5v3.l2
## Xcb5v3.l3
## Xcb5v3.l4
## Xcb5v3.l5
## Xcb6v1.l1
## Xcb6v1.l2
## Xcb6v1.l3
## Xcb6v1.l4
## Xcb6v1.l5
## Xcb6v2.l1
## Xcb6v2.l2
## Xcb6v2.l3
## Xcb6v2.l4
## Xcb6v2.l5
## Xcb6v3.l1
## Xcb6v3.l2
## Xcb6v3.l3 -0.623
## Xcb6v3.l4 -0.359 -0.029
## Xcb6v3.l5 0.180 -0.486 -0.105
## Xs(NumDate)Fx1 0.002 0.001 -0.006 0.005
##
## Standardized Within-Group Residuals:
## Min Q1 Med Q3 Max
## -1.5296928 -0.4521492 -0.2317115 0.1917924 26.1176478
##
## Number of Observations: 21311
## Number of Groups:
## g country_region %in% g
## 1 123

summary(fit1$gam)

##
## Family: Tweedie(1.656)
## Link function: log
##
## Formula:
## IncCaseNumber ~ s(NumDate)
##
## Parametric coefficients:
## Estimate Std. Error t value Pr(>|t|)
## (Intercept) 3.2864 0.2182 15.06 <2e-16 ***
## ---
## Signif. codes: 0 '***' 0.001 '**' 0.01 '*' 0.05 '.' 0.1 ' ' 1
##
## Approximate significance of smooth terms:
## edf Ref.df F p-value
## s(NumDate) 8.85 8.85 1930 <2e-16 ***
## ---
## Signif. codes: 0 '***' 0.001 '**' 0.01 '*' 0.05 '.' 0.1 ' ' 1
##
## R-sq.(adj) = 0.00779
## Scale est. = 9.281 n = 21311

summary(fit2$gam)

##
## Family: Tweedie(1.656)
## Link function: log
##
## Formula:
## IncCaseNumber ~ s(NumDate) + s(retail_and_recreation_percent_change_from_baseline) +
## s(grocery_and_pharmacy_percent_change_from_baseline) + s(transit_stations_percent_change_from_baseline) +
## s(workplaces_percent_change_from_baseline + residential_percent_change_from_baseline)
##
## Parametric coefficients:
## Estimate Std. Error t value Pr(>|t|)
## (Intercept) 3.1366 0.1947 16.11 <2e-16 ***
## ---
## Signif. codes: 0 '***' 0.001 '**' 0.01 '*' 0.05 '.' 0.1 ' ' 1
##
## Approximate significance of smooth terms:
## edf Ref.df F
## s(NumDate) 8.967 8.967 964.80
## s(retail_and_recreation_percent_change_from_baseline) 8.110 8.110 142.59
## s(grocery_and_pharmacy_percent_change_from_baseline) 7.854 7.854 108.20
## s(transit_stations_percent_change_from_baseline) 6.436 6.436 40.26
## s(workplaces_percent_change_from_baseline) 6.663 6.663 52.27
## p-value
## s(NumDate) <2e-16 ***
## s(retail_and_recreation_percent_change_from_baseline) <2e-16 ***
## s(grocery_and_pharmacy_percent_change_from_baseline) <2e-16 ***
## s(transit_stations_percent_change_from_baseline) <2e-16 ***
## s(workplaces_percent_change_from_baseline) <2e-16 ***
## ---
## Signif. codes: 0 '***' 0.001 '**' 0.01 '*' 0.05 '.' 0.1 ' ' 1
##
## R-sq.(adj) = 0.00929
## Scale est. = 8.4852 n = 21311

summary(fitcb$gam)

##
## Family: Tweedie(1.656)
## Link function: log
##
## Formula:
## IncCaseNumber ~ s(NumDate) + cb1 + cb2 + cb4 + cb5 + cb6
##
## Parametric coefficients:
## Estimate Std. Error t value Pr(>|t|)
## (Intercept) 5.02097 0.45929 10.932 < 2e-16 ***
## cb1v1.l1 0.37486 0.37218 1.007 0.313844
## cb1v1.l2 -0.25501 0.36182 -0.705 0.480936
## cb1v1.l3 0.14325 0.34750 0.412 0.680171
## cb1v1.l4 1.78336 0.25651 6.952 3.70e-12 ***
## cb1v1.l5 -1.16022 0.32616 -3.557 0.000376 ***
## cb1v2.l1 0.94467 0.36610 2.580 0.009877 **
## cb1v2.l2 0.64631 0.36002 1.795 0.072638 .
## cb1v2.l3 -0.64301 0.34678 -1.854 0.063720 .
## cb1v2.l4 0.88152 0.24384 3.615 0.000301 ***
## cb1v2.l5 -2.08204 0.30493 -6.828 8.85e-12 ***
## cb1v3.l1 -1.01595 0.48874 -2.079 0.037658 *
## cb1v3.l2 -0.47463 0.47830 -0.992 0.321046
## cb1v3.l3 -1.34291 0.45965 -2.922 0.003486 **
## cb1v3.l4 2.87059 0.33049 8.686 < 2e-16 ***
## cb1v3.l5 -2.24728 0.41121 -5.465 4.68e-08 ***
## cb2v1.l1 -0.19336 0.42569 -0.454 0.649664
## cb2v1.l2 0.11129 0.40312 0.276 0.782491
## cb2v1.l3 0.59179 0.39339 1.504 0.132515
## cb2v1.l4 -1.77323 0.27471 -6.455 1.11e-10 ***
## cb2v1.l5 2.71376 0.33027 8.217 < 2e-16 ***
## cb2v2.l1 -0.42769 0.33003 -1.296 0.195020
## cb2v2.l2 -0.36221 0.31280 -1.158 0.246897
## cb2v2.l3 -0.23565 0.29480 -0.799 0.424100
## cb2v2.l4 0.71847 0.21097 3.405 0.000662 ***
## cb2v2.l5 0.14422 0.25955 0.556 0.578441
## cb2v3.l1 -0.13309 0.51501 -0.258 0.796082
## cb2v3.l2 0.28777 0.47611 0.604 0.545570
## cb2v3.l3 1.65404 0.45209 3.659 0.000254 ***
## cb2v3.l4 -4.60325 0.31509 -14.609 < 2e-16 ***
## cb2v3.l5 4.74375 0.37408 12.681 < 2e-16 ***
## cb4v1.l1 -0.46557 0.44257 -1.052 0.292823
## cb4v1.l2 -0.51557 0.43079 -1.197 0.231397
## cb4v1.l3 -0.33734 0.41717 -0.809 0.418724
## cb4v1.l4 -1.23164 0.29593 -4.162 3.17e-05 ***
## cb4v1.l5 0.28138 0.35039 0.803 0.421959
## cb4v2.l1 -0.01728 0.38360 -0.045 0.964068
## cb4v2.l2 0.26641 0.36884 0.722 0.470114
## cb4v2.l3 0.95394 0.35586 2.681 0.007353 **
## cb4v2.l4 0.14627 0.24478 0.598 0.550154
## cb4v2.l5 1.22186 0.30587 3.995 6.50e-05 ***
## cb4v3.l1 -0.38479 0.58197 -0.661 0.508498
## cb4v3.l2 -0.64022 0.55258 -1.159 0.246636
## cb4v3.l3 -0.58718 0.53262 -1.102 0.270286
## cb4v3.l4 -0.58348 0.37879 -1.540 0.123478
## cb4v3.l5 -0.33732 0.44486 -0.758 0.448301
## cb5v1.l1 -0.55033 0.49525 -1.111 0.266487
## cb5v1.l2 0.43591 0.48621 0.897 0.369971
## cb5v1.l3 0.68376 0.46118 1.483 0.138186
## cb5v1.l4 -1.56423 0.33798 -4.628 3.71e-06 ***
## cb5v1.l5 0.02859 0.40990 0.070 0.944392
## cb5v2.l1 0.36231 0.59023 0.614 0.539324
## cb5v2.l2 -0.35016 0.57029 -0.614 0.539216
## cb5v2.l3 -1.44865 0.54593 -2.654 0.007971 **
## cb5v2.l4 3.12406 0.40288 7.754 9.28e-15 ***
## cb5v2.l5 -0.93245 0.50259 -1.855 0.063566 .
## cb5v3.l1 -0.47546 1.19741 -0.397 0.691316
## cb5v3.l2 1.08099 1.16164 0.931 0.352087
## cb5v3.l3 2.91750 1.11240 2.623 0.008729 **
## cb5v3.l4 -7.78232 0.83566 -9.313 < 2e-16 ***
## cb5v3.l5 2.91029 1.03196 2.820 0.004804 **
## cb6v1.l1 -0.05301 0.45783 -0.116 0.907816
## cb6v1.l2 0.13950 0.44637 0.313 0.754649
## cb6v1.l3 -0.33096 0.42599 -0.777 0.437213
## cb6v1.l4 -0.01081 0.28609 -0.038 0.969845
## cb6v1.l5 0.35809 0.36194 0.989 0.322508
## cb6v2.l1 -0.06736 0.33371 -0.202 0.840040
## cb6v2.l2 0.26218 0.32564 0.805 0.420757
## cb6v2.l3 -0.31023 0.30843 -1.006 0.314507
## cb6v2.l4 0.52968 0.21172 2.502 0.012363 *
## cb6v2.l5 -0.66860 0.28418 -2.353 0.018645 *
## cb6v3.l1 -0.46321 0.43401 -1.067 0.285854
## cb6v3.l2 0.62281 0.42379 1.470 0.141677
## cb6v3.l3 -0.69376 0.40089 -1.731 0.083544 .
## cb6v3.l4 0.01649 0.27641 0.060 0.952438
## cb6v3.l5 -0.01086 0.36454 -0.030 0.976238
## ---
## Signif. codes: 0 '***' 0.001 '**' 0.01 '*' 0.05 '.' 0.1 ' ' 1
##
## Approximate significance of smooth terms:
## edf Ref.df F p-value
## s(NumDate) 8.974 8.974 622.6 <2e-16 ***
## ---
## Signif. codes: 0 '***' 0.001 '**' 0.01 '*' 0.05 '.' 0.1 ' ' 1
##
## R-sq.(adj) = 0.00876
## Scale est. = 8.6135 n = 21311

plot(fit1$lme)


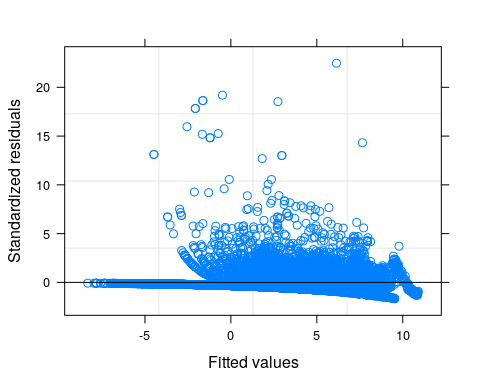


plot(fit1$gam)


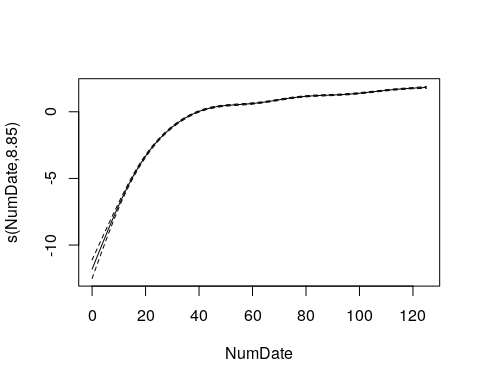


plot(fit2$lme)


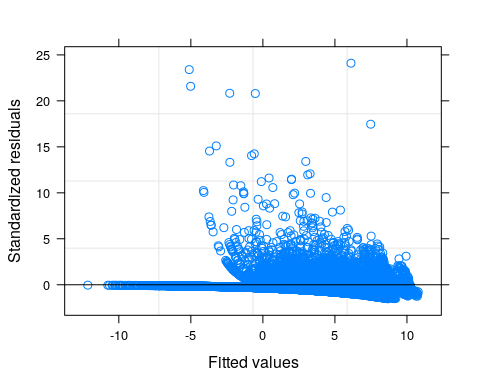


plot(fit2$gam)


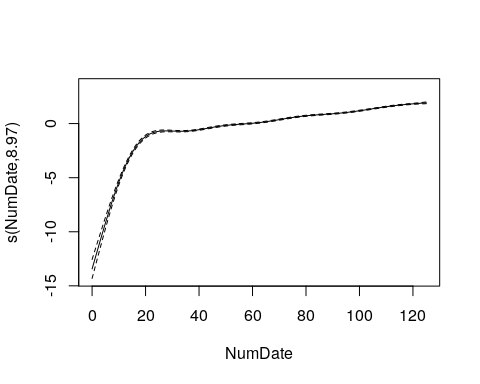

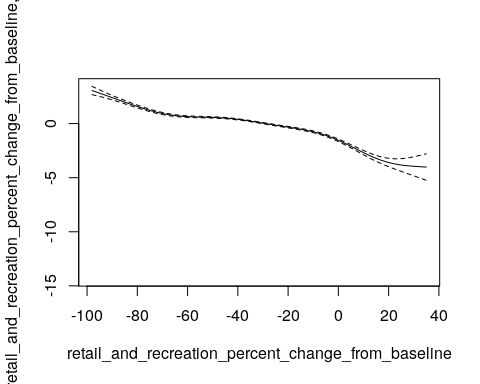

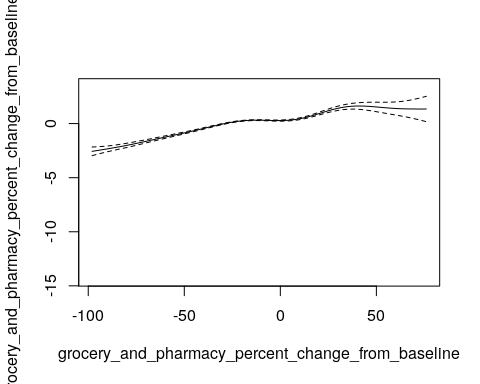

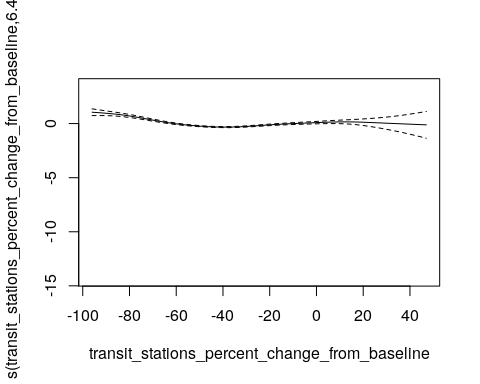

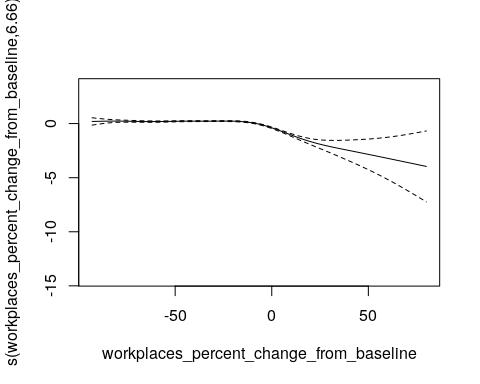


plot(fitcb$lme)


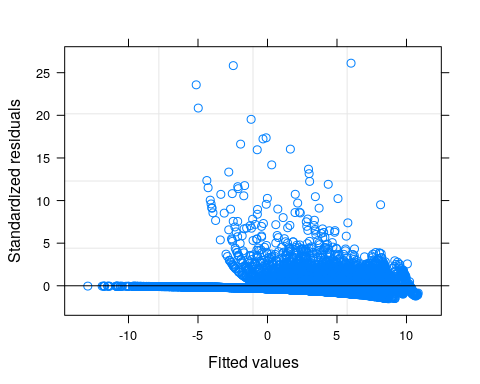


plot(fitcb$gam)


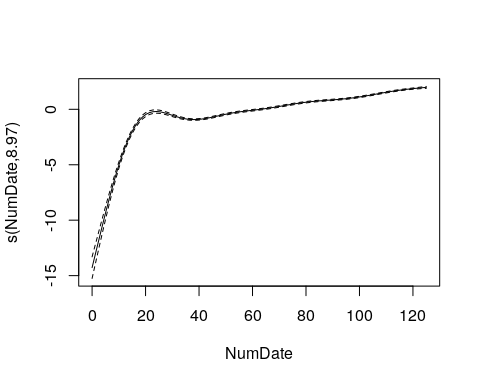


#preparing data for the validation

cb1 <- crossbasis(datav$grocery_and_pharmacy_percent_change_from_baseline , lag=14, argvar=list(fun="bs"),arglag=list(df=5), group=datav$country_region)
cb2 <- crossbasis(datav$retail_and_recreation_percent_change_from_baseline , lag=14, argvar=list(fun="bs"),arglag=list(df=5), group=datav$country_region)
cb4 <- crossbasis(datav$transit_stations_percent_change_from_baseline , lag=14, argvar=list(fun="bs"),arglag=list(df=5), group=datav$country_region)
cb5 <- crossbasis(datav$workplaces_percent_change_from_baseline , lag=14, argvar=list(fun="bs"),arglag=list(df=5), group=datav$country_region)
cb6 <- crossbasis(datav$residential_percent_change_from_baseline , lag=14, argvar=list(fun="bs"),arglag=list(df=5), group=datav$country_region)

dataval<-datav %>%
 group_by(country_region) %>%
 slice(-c(1:14))


pred1<-predict.gam(fit1$gam, dataval, type="response")
pred2<-predict.gam(fit2$gam, dataval, type="response")
predcb<-predict.gam(fitcb$gam, datav, type="response")
predcb<-predcb[!is.na(predcb)]


RMSE <- function(pred, obs){
 sqrt(mean((pred - obs)^2))
}


RMSE(pred1, dataval$IncCaseNumber)

## [1] 6840.163

RMSE(pred2, dataval$IncCaseNumber)

## [1] 6794.985

RMSE(predcb, dataval$IncCaseNumber) #lag distributed is slightly better than contempr.-worst is the without GMD

## [1] 6690.436

dataval$pred1<-pred1
dataval$pred2<-pred2
dataval$predcb<-predcb

rmsepercountry1<-dataval %>%
 group_by(country_region) %>%
 summarise(RMSE(pred1, IncCaseNumber), .groups = 'drop')
summary(rmsepercountry1)

## country_region RMSE(pred1, IncCaseNumber)
## Afghanistan : 1 Min. : 113.2
## Angola : 1 1st Qu.: 240.9
## Antigua and Barbuda: 1 Median : 279.6
## Argentina : 1 Mean : 2111.3
## Australia : 1 3rd Qu.: 728.2
## Austria : 1 Max. :58437.4
## (Other) :117

rmsepercountry2<-dataval %>%
 group_by(country_region) %>%
 summarise(RMSE(pred2, IncCaseNumber), .groups = 'drop')
summary(rmsepercountry2)

## country_region RMSE(pred2, IncCaseNumber)
## Afghanistan : 1 Min. : 33.0
## Angola : 1 1st Qu.: 163.7
## Antigua and Barbuda: 1 Median : 289.9
## Argentina : 1 Mean : 2062.2
## Australia : 1 3rd Qu.: 682.2
## Austria : 1 Max. :57920.9
## (Other) :117

rmsepercountrycb<-dataval %>%
 group_by(country_region) %>%
 summarise(RMSE(predcb, IncCaseNumber), .groups = 'drop')
summary(rmsepercountrycb)

## country_region RMSE(predcb, IncCaseNumber)
## Afghanistan : 1 Min. : 12.9
## Angola : 1 1st Qu.: 217.8
## Antigua and Barbuda: 1 Median : 412.8
## Argentina : 1 Mean : 2083.1
## Australia : 1 3rd Qu.: 732.7
## Austria : 1 Max. :56784.6
## (Other) :117

library(forecast)

## Registered S3 method overwritten by 'quantmod':
## method from
## as.zoo.data.frame zoo

##
## Attaching package: 'forecast'

## The following object is masked from 'package:nlme':
##
## getResponse

dm.test(pred1-dataval$IncCaseNumber, pred2-dataval$IncCaseNumber)

##
## Diebold-Mariano Test
##
## data: pred1 - dataval$IncCaseNumberpred2 - dataval$IncCaseNumber
## DM = 13.42, Forecast horizon = 1, Loss function power = 2, p-value <
## 2.2e-16
## alternative hypothesis: two.sided

dm.test(predcb-dataval$IncCaseNumber, pred2-dataval$IncCaseNumber)

##
## Diebold-Mariano Test
##
## data: predcb - dataval$IncCaseNumberpred2 - dataval$IncCaseNumber
## DM = -13.754, Forecast horizon = 1, Loss function power = 2, p-value <
## 2.2e-16
## alternative hypothesis: two.sided

dm.test(predcb-dataval$IncCaseNumber, pred1-dataval$IncCaseNumber)

##
## Diebold-Mariano Test
##
## data: predcb - dataval$IncCaseNumberpred1 - dataval$IncCaseNumber
## DM = -14.2, Forecast horizon = 1, Loss function power = 2, p-value <
## 2.2e-16
## alternative hypothesis: two.sided
